# Outdoor play in Europe: terminology and state of research, practice, and policy

**DOI:** 10.1093/eurpub/ckag040

**Published:** 2026-03-28

**Authors:** Lærke Mygind, Avril Johnstone, Artan R Kryeziu, Benjamin Billet, Christine Delisle Nyström, Ellen Beate Sandseter, Evelin Mäestu, Getter Marie Lemberg, Gillian Cante, Helen Dodd, Javier Brazo-Sayavera, Juel Jarani, Kamil Maciaszek, Konstantina Rentzou, Mads Bølling, Manon A T Bloemen, Marianne Mannello, Marie Bradwell, Marie Löf, Marjaana Kangas, Mark Leather, Michelle Bergin, Nika Bezjak, Peter Bakalár, Sanja Salaj, Shawnda A Morrison, Signe Siklander, Silvia Veiga-Seijo, Stevo Popovic, Tamás Csányi, Thomas Morgenthaler, Peter Bentsen

**Affiliations:** Center for Clinical Research and Prevention, Copenhagen University Hospital—Bispebjerg and Frederiksberg, The Capital Region, Denmark; MRC/CSO Social and Public Health Sciences Unit, School of Health and Wellbeing, University of Glasgow, Glasgow, United Kingdom; Faculty of Physical Education and Sports, University of Pristina, Pristina, Kosovo; Center of Research, Studies in Physical Education, Sport and Health-CRSPES, Pristina, Kosovo; European Network of Outdoor Sports and National Centre for Outdoor Sports, Ministry of Sports, Vallon-Pont-d’Arc, France; Department of Medicine Huddinge, Karolinska Institutet, Huddinge, Sweden; Department for Physical Education and Health, Queen Maud University College, Trondheim, Norway; Institute of Sport Sciences and Physiotherapy, Faculty of Medicine, University of Tartu, Tartu, Estonia; Institute of Sport Sciences and Physiotherapy, Faculty of Medicine, University of Tartu, Tartu, Estonia; Sport and Social Sciences Laboratory, Health and Adapted Physical Activity Unit, University of Strasbourg, Strasbourg, France; Department of Public Health and Sports Sciences, Exeter Medical School, University of Exeter, Exeter, United Kingdom; Department of Sports and Computer Science, Universidad Pablo de Olavide, Sevilla, Spain; Act4edu Center, Tirana, Albania; GratoSfera Foundation, Gdańsk, Poland; Department of Early Years Learning and Care, University of Ioannina, Ioannina, Greece; Center for Clinical Research and Prevention, Copenhagen University Hospital—Bispebjerg and Frederiksberg, The Capital Region, Denmark; VIA University College, Research Centre for Didactics and Pedagogy, Program on Outdoor Pedagogy, Aarhus, Denmark; Research Group Moving, Growing and Thriving Together, University of Applied Sciences Utrecht, Utrecht, The Netherlands; Play Wales, Park House, Cardiff, United Kingdom; School of Education, Plymouth Marjon University, Plymouth, United Kingdom; Department of Medicine Huddinge, Karolinska Institutet, Huddinge, Sweden; Faculty of Education, University of Lapland, Rovaniemi, Finland; School of Education, Plymouth Marjon University, Plymouth, United Kingdom; School of Clinical Therapies, University College Cork, Cork, Ireland; Faculty of Sport, University of Ljubljana, Ljubljana, Slovenia; Faculty of Sports, University of Prešov, Prešov, Slovakia; Faculty of Physical Culture, Palacký University Olomouc, Olomouc, Czechia; Faculty of Kinesiology, University of Zagreb, Zagreb, Croatia; Faculty of Sport, University of Ljubljana, Ljubljana, Slovenia; Human Potential Translational Research Programme, Yong Loo Lin School of Medicine, National University of Singapore, Singapore, Singapore; Faculty of Education and Psychology, University of Oulu, Oulu, Finland; Health Sciences Department, Faculty of Health Sciences, University of A Coruña, A Coruña, Spain; Division of Occupational Therapy and Arts Therapies, Queen Margaret University, Edinburgh, United Kingdom; Department of Occupational Science and Occupational Therapy, University College Cork, Cork, Ireland; Faculty for Sport and Physical Education, University of Montenegro, Niksic, Montenegro; Western Balkan Sport Innovation Lab, Podgorica, Montenegro; K-Club, Korea University, Seoul, Republic of Korea; Department of Physical Education Theory and Methodology, Hungarian University of Sports Science, Budapest, Hungary; MTA-ELTE Psychomotor Development Research Group, Hungarian Academy of Sciences, Budapest, Hungary; Division of Occupational Therapy and Arts Therapies, Queen Margaret University, Edinburgh, United Kingdom; Department of Occupational Science and Occupational Therapy, University College Cork, Cork, Ireland; Center for Clinical Research and Prevention, Copenhagen University Hospital—Bispebjerg and Frederiksberg, The Capital Region, Denmark; Department of Geosciences and Natural Resource Management, University of Copenhagen, Frederiksberg, Denmark

## Abstract

Outdoor play (OP) is strongly associated with human health, development, and wellbeing, yet modern lifestyles and changing environments increasingly restrict opportunities for OP. The state of OP in Europe is not well understood, as there is limited alignment among researchers, practitioners, and policymakers in defining, researching, or promoting opportunities for OP within communities and across Europe more broadly. This study aims to offer a comprehensive assessment of the European OP system by mapping its core elements—terminology, research, practice, and policy. This mixed-methods study collected data through expert-elicitation from countries across Europe, with particular focus on traditionally underrepresented regions. Although there has recently been more emphasis on the health benefits of OP, conceptual and ontological differences relating to OP continue to challenge any consistent harmonization or cross-country comparison processes. Based on these observations, we formulate actions to strengthen the OP system across Europe, including enhancing monitoring, promoting supportive policy, and leveraging international, cross-disciplinary forums. Coordinated researcher and practitioner networks can identify priorities, support mission-driven projects, and inform Europe-level policies to expand OP opportunities and drive positive, resilient continental health outcomes.

## Introduction

The importance of outdoor play (OP) for human health and wellbeing is well-recognized [1, 2]; however, modern lifestyles and rapidly changing environments are placing increasing restrictions on opportunities for children and adults to engage in OP (e.g. [3, 4]). Reductions in OP due to external factors, including COVID-19 lockdowns, have been linked to lower wellbeing, while higher pre-pandemic OP levels were shown to support mental health [5]. Data from the Active Healthy Kids Global Alliance indicate substantial variation in children’s active play across European countries, reflecting differences in daily play time and access to safe environments [6], highlighting the need for improved access to safe, well-maintained play spaces across the region. Promoting OP aligns with several Sustainable Development Goals (SDGs), such as good health (SDG 3) and equitable access to public spaces (SDGs 10 and 11).

While OP has gained increasing attention in research, policy, and practice, especially over the past decade, much of this work remains fragmented at regional, national, or local levels. In Europe–where cultural, political, and governance contexts vary considerably—OP provision is often shaped by national or regional policies influenced by international frameworks like the Declaration of Human Rights, Article 24 [7], UN Convention on the Rights of the Child [8], and the General Comment 17 from the UN [9]. Despite these global mandates, a 2018 review found only a few European countries had dedicated national play policies—specifically Ireland and the UK—highlighting a significant gap in formal policy support [10].

Currently, there is no consolidated overview of how OP is conceptualized, supported, and implemented across countries. To address this, we conceptualize OP as a dynamic system where research, practice, and policy interact to influence OP opportunities and experiences. Framing OP as a system allows us to analyse how variations in terminology, national policies, and organizational practices define current capabilities while exposing the inequalities that necessitate coordinated action. By synthesizing current research, policy, and practice, this paper identifies shared challenges and highlights barriers to more unified and inclusive approaches. Ultimately, this integrated analysis provides a foundation for cross-national learning and supports the development of coherent strategies to advance equitable OP opportunities for children, adolescents, and adults across the region.

## Methods

This study followed a modified stepwise methodology from Carl *et al.* [11] using a mix of expert-elicited data supplemented with existing Global Matrix 4.0 data.

### Expert identification

European OP experts were identified using a snowball sampling approach, beginning with the AOP10 steering committee [12] and extended through targeted invitations to European participants of the 2023 International Play Association Conference and additional referrals. Eligibility required residence or professional affiliation in Europe, demonstrated expertise in OP through advocacy or peer-reviewed publications, and completion of one or more country-specific OP data forms.

This resulted in participation from six countries: Denmark (M.Bø, P.Be), Norway (E.B.S.), Poland (K.M.), Slovenia (S.A.M., N.B.), Spain (S.V.S., J.B.S.), United Kingdom [i.e. the nation states England and North Ireland (H.D., M.L.) and Scotland (A.J.)]. One author from Slovakia (P.Ba) reached out to the main author after approaching the AOP10 Project leadership. The resultant pool of authors was then asked to provide further points of reference towards potential candidates from non-represented countries. This led to the inclusion of authors from 12 additional countries: Albania (J.J.), Austria (T.M.), Croatia (S.S.), Estonia (E.M., G.M.L.), Finland (M.K., S.S.), France (B.B., G.C.), Greece (K.R.), Hungary (T.C.), Ireland (T.M, M.Be), Montenegro (S.P.), the Netherlands (M.Bl), and Sweden (M.L., C.D.N.). To improve coverage, we also included an expert from Kosovo (A.K.) although not listed on UN’s list of independent countries and an additional representative from United Kingdom [i.e. the nation state Wales (M.M.)].

Two experts expressed their initial interest in contributing to the study (Switzerland, Ukraine), but had to withdraw or did not respond to multiple e-mails and, thus, were withdrawn from the process. All experts provided consent to contribute to this project and to work together constructively in two structured online dialogue sessions.

### Expert-elicited data

A data form was developed based on overarching themes, i.e. research, practice, and policy, identified by Carl *et al.* [11].

We then adapted these themes to align with the foci and timeline of the AOP10 project by:

1. splitting the research theme into researchers (prompt: ‘Name active OP researchers and describe their research focus’) and scientific publications and reports (‘Describe scope of scientific publications and reports on OP stemming from country in question (please include references of seminal publications and publications that relate to gender, race, class, or climate)’),
2. dividing practice into two pre-defined types, i.e., organizations (‘Name and describe organizations (e.g. OP Canada) that support and promote OP’) and networks (‘Name and describe networks (e.g. PLaTO-Net) that support and promote OP’), and
3. splitting policy into policy documents (‘Name and describe national policy documents that support OP’) and funding bodies (‘Name and describe fundings bodies that support OP’).
4. finally, the data form included an open-ended ‘other’ section in which the experts could write any necessary nuances or other supporting information.

The data form was presented to the author group at an introductory meeting and where experts discussed and adapted the form further for clarity. During this meeting, it became clear that the distinct terminology used to conceptualize OP varied greatly across the European countries, and that this might contribute to misunderstandings and mistranslations if not addressed explicitly within the larger investigation. Terminology around OP was, therefore, included as a distinct theme in the data form (‘*Please name and describe terms that are used in country in question that could be conceptualised as or include OP. Please also comment on how PA relates to these terms*’).

One form was completed per country, with some forms completed collaboratively by multiple experts. Several authors contacted local stakeholders for input and validation. It was decided in the authorship group that the individual legislative and cultural settings of the individual nations of the UK (Scotland, England, Northern Ireland, and Wales) required separate analysis, although some UK-wide policies exist. The completed data forms can be found in Appendix S1.

Data were extracted by the first author (L.M.) and validated by a second author (A.J.) who confirmed the extracted data against content from a random sample of the data forms (∼20% of all the completed forms, *n *= 5). Differences were resolved through discussion between L.M. and A.J., and the findings were narratively synthesized by L.M. and verified by all authors for accuracy.

### Analysis

Research topics were categorized according to the themes identified as part of the AOP10 Project [13]. Organizations and policies were categorized according to types listed in Table 1. Country-specific OP translations and conceptualizations were extracted from the data forms and listed in Table 2.

**Table 1.** Types of organizations and policies

Type	Abbr.	Description
Organizations		
Governmental bodies	GB	A governmental body is any nation or government, any state or other political subdivision thereof or any entity, authority, agency, division, or department exercising the legislative, judicial, regulatory, or administrative functions of or pertaining to a government.
Specialist organizations	SO	Specialist organizations or interest organizations are a formally organized association that seeks to influence public policy.
Charity	C	A charity is an organization whose primary objectives are philanthropy and social wellbeing. Charitable organizations may not use any of their funds to profit individual persons or entities.
*Charity, priority area*	*C, PA*	A charity may choose certain priority areas that they will direct support towards.
Regulative bodies	RB	A regulatory body is a public organization or government agency that is responsible for legally regulating aspects of human activity.
Policies		
Legislative	L	Legislation is the process or result of enrolling, enacting, or promulgating laws by a legislature, parliament, or analogous governing body.
Regulative	R	Regulation is the controlling of an activity or process, usually by means of rules.
Delivery	D	Delivery plan is a document outlining detailed plans to support the delivery of a government programme.
Advocacy/strategy	A	Advocacy is an action-orientated document used to facilitate policy making decisions or influence/advocate for specific actions on an issue or problem.

**Table 2.** Country-specific conceptualization of OP

Country	Key concepts
Albania (AL)	+ Literal translation of OP is ‘lojërat në natyrë’—often includes traditional games [‘dy gurë’ (two stones), ‘kukafshehti’ (hide and seek), and ‘sfida ndërmjet fëmijëve’ (children’s battle)], group sports, or free play that often takes place in village squares or open fields + Games outside (‘lojëra jashtë’) + Physical activities in nature (‘aktivitete fizike në natyrë’)—often used to emphasize physical engagement + Games in open air (‘lojëra në mjedis të hapur’) + Entertainment activities in nature (‘veprimtari argëtuese në natyrë’) + Games in the field (‘lojëra në fushë’)
Austria (AT)	+ Playing outdoors (‘Spielen im Freien’ or ‘draußen spielen’) + Free play (‘Freispiel’ or ‘freies Spiel’)—play that is guided by the child, often in preschool settings + PA outdoors (‘Bewegung im Freien’)
Croatia/Hrvatska (HR)	+ Literal translation of OP is ‘igra na otvorenom’—not commonly used, primary focus on children + Outdoor stay (‘boravak na otvorenom’), stay in fresh air (‘boravak na svježem zraku’), outdoor activities (‘aktivnosti na otvorenom’), or activities in nature (‘aktivnosti u prirodi’) more commonly used + Terms ‘učenje u prirodi’ and ‘škola u prirodi’ are used in education + Terms more closely related to PA are PA in nature (‘Tjelesna aktivnost u prirodi’), physical exercise in nature (‘Tjelesno vježbanje u prirodi’ or ‘Tjelovježba u prirodi’), active stay in nature (‘Aktivni boravak u prirodi’), and actively spending time in nature (‘Aktivno provođenje vremena u prirodi’).
Denmark (DK)	+ Literal translation of OP is ‘udeleg’—often involves non-sedentary activities + Nature kindergartens (‘naturbørnehaver’, ‘skovbørnehaver’) + Outdoor life (‘friluftsliv’) + PA with no competitive aim (‘idræt’) + Education outside the classroom (‘udeskole’)—often includes playful learning
Estonia (EE)	+ Literal translation of OP is ‘õues mängimine’—not necessarily active + Playgrounds (‘mänguväljak’) are common and school playgrounds open after school + Outdoor recess (‘õuevahetund’) is a growing trend + Outdoor kindergarten (‘õuelastehoid’ or ‘õuelasteaed’) are not common in Estonia, but are starting to appear
Finland (FI)	+ Literal translation of OP is ‘ulkoleikki’ + Outdoor ECEC (‘ulkopäiväkoti’, ‘luontopäiväkoti’, ‘metsäpäiväkoti’) are common + Adventure education (‘seikkailukasvatus’) incorporates play in outdoor activities + Outdoor playgrounds for children (‘ulkoleikkipuisto’) and adults (‘liikuntaleikkipuisto’) + Recess outdoor facilities at schools and ECEC ‘välituntipiha’ ‘piha’
France (FR)	+ No direct or formal translation + Free OP (‘Jeu libre en extérieur’)—often used in alternative pedagogies taking place in natural environments + Outdoor class (‘École dehors’/ ‘Classe dehors’)—spending part of a school day outdoors + Nature-based education (‘Éducation par la nature’)—outdoor activities to reconnect children with nature + Forest schools (‘Ecole de forêt’)—Inspired by German forest schools but pertain mainly to extra curricular activities
Greece (GR)	+ Literal translation of OP is ‘υπαίθριο παιχνίδι’—typically involves PA and exploration + Play in the yard (‘Παιχνίδι στην αυλή’)—OP at school or home + Free play in outdoor spaces (‘Eλεύθερο παιχνίδι σε εξωτερικούς χώρους’)—emphasizes free nature of play + Play in the neighborhood (‘Παιχνίδι στη γειτονιά’) refers to playing freely with no playground available
Hungary (HU)	+ Literal translation of OP is ‘szabadtéri/kültéri játék’—used to describe any type of play outdoors without adult supervision + Strong tradition for OP in early education and care (ECEC), schools, and family life
Ireland (IE)	+ Literal translation of OP is ‘ag súgradh amuigh’ emphasizes free play or ‘ag imirt amuigh’ which references moreso rules based games and physical activities. + OP—used similarly to PLaTO-Net + Risky play—also used but less frequently + Recreational PA (adults)
Kosovo (KSV)	+ Active OP is not a commonly used term + Recreation activities + Out of school activities and sports
Montenegro (ME)	+ Literal translation of OP is ‘gra na otvorenom’—OP for children and adults + Play in the meadow (‘igra na livadi’)—unstructured, physically active OP in natural settings + Outing/Excursion (‘izlet’) involves families/groups going on day trips to natural areas—often involves OP activities + Outdoor sports (‘sport na otvorenom’)—structured and unstructured PA taking place outdoors
The Netherlands (NL)	+ Literal translation of OP is ‘buitenspelen’ or ‘buitenspelen’—commonly used and ranges from voluntary engagement to guided play + Playing outside the house (‘buitenshuis spelen’) + Going outside (‘naar buiten gaan’) + Risky play (‘risicovol spelen’ or ‘avontuurlijk spelen’)—mostly refers to playing outside
Norway (NO)	+ Literal translation of OP is ‘utelek’—often associated with childhood/adolescence + ‘Friluftsliv’ + Outdoor kindergartens (‘utebarnehage’) focusing on OP and activities are common. + Outdoor school (‘uteskole’) contains some degree of free OP + The law of common access to nature (‘allemannsretten’)
Poland (PL)	+ No direct or formal translation, closest equivalents are play in the fresh air (‘zabawa na świeżym powietrzu’) and outdoor activity (‘aktywność na zewnątrz’) + PA in the fresh air (‘aktywność fizyczna na świeżym powietrzu’) and movement-based OP (‘zabawy ruchowe na dworze’) used in education and health promotion + Play with loose parts (‘zabawa gratami’)
Slovakia (SK)	+ Limited consensus around the meaning of OP + The most common translation of OP is ‘hra vonku’ (singular form) or ‘hry vonku’ (plural form) + More meaningful back-translation of OP is ‘outside play’ or ‘playing outside’ + Outdoor activities ‘outdoorové aktivity’ superordinate term to ‘outside play/playing outside’ in academics + ‘Play in nature’
Slovenia (SI)	+ Literal translation of OP is ‘igra na prostem’ + Playgrounds (‘igrišče’) + Outdoor playgrounds (‘zunanje igrišče’) + Risky play (‘tvegana igra’) + Free play (‘prosta igra’) + Nature play (‘igra v naravi’) + Outdoor education (‘izobraževanje na prostem’) + Forest pedagogy (‘gozdna pedagogika’) + School in nature (‘šola v naravi’) + Leisure activity in nature (‘zunanja rekreacija’) + PA (‘telesna dejavnost’) + Green areas (‘zelene površine’)
Spain/España (ES)	+ Literal translation of OP is ‘juego fuera’—not commonly used + Playing outside home (‘juego al aire libre’ or ‘jugar fuera de casa’) or play on the street (‘jugar en la calle’) more commonly used
Sweden (SW)	+ Literal translation of OP is ‘utelek’ or ‘aktiv utelek’—not commonly used + ‘Friluftsliv’ + General emphasis of being outdoors
United Kingdom (UK)^a^	+ OP, active play, nature play, risky play, playing out—used similarly to PLaTO-Net + OP and learning + Recreational PA (adults) + Children’s street play + Active OP (not commonly used) + Risky/adventurous play

a Including nation states; England, Northern Ireland, Wales, and Scotland.

Drawing on Carl *et al.* [11], the data categorizations underwent comparative document analysis using a transnational four-eyes principle, involving a researcher from another country (A.J.). This involved:

- Reading the material for a random sample of countries for familiarization.
- Narrative assessment—e.g. analysing the focus of researchers, organization, policy types, and the policies’ content.
- Interpretive validation—e.g. the conclusions derived on basis of the analyses.

### Validation

Extracted information on OP policies were cross-referenced with the policies recorded in the Global Matrix 4.0 report cards [6]. Any additional documents sourced from the report cards were added to the results table, and additional policies from countries not represented within the expert group were also added, if available (i.e. Belgium, Bulgaria, Czech Republic, Germany, Lithuania, Portugal, and Serbia).

All experts were invited to actively participate in a concluding online meeting during which representatives of the participating countries discussed the findings of the study in detail (i.e. communicative validation) and identified future directions for OP efforts in Europe.

## Results

This study communicates the expert knowledge of representatives from 20 of 45 (including Kosovo) European countries. No additional OP-relevant information was found through the scan of Global Matrix 4.0 data.

### Outdoor play terminology

Terminology used to describe OP varied considerably across European countries (see Table 2), reflecting linguistic, cultural, and contextual differences. These differences illustrate one component of the OP system: how language and local understanding shape recognition, measurement, and support of OP opportunities. While the term ‘outdoor play’ was commonly used in English-speaking contexts, many non-English-speaking countries employed more locally embedded expressions. In some cases, these terms foregrounded specific locations, such as ‘play in the meadow’ in Montenegro or ‘play in the neighbourhood or yard’ in Greece. In others, the emphasis was placed on being outdoors more generally, without explicit reference to play, as seen in terms such as ‘outdoor stay’ in Croatia or ‘outdoor life’ (‘friluftsliv’) in Scandinavian countries. This broader framing suggests that play may be implicitly understood as an integral component of outdoor presence rather than a separately defined activity. These variations highlight both the richness and complexity of OP practices and the potential for misunderstandings across regions.

Another pattern was the close association between OP and educational practices in several countries. Terms such as ‘education outside the classroom’ (‘udeskole’) in Scandinavia reflect long-standing traditions of integrating outdoor experiences with learning. By contrast, some countries drew clearer conceptual distinctions between play and PA, using specific terminology to denote outdoor PA, as in the Polish context (‘PA in the outdoors’). Together, these variations highlight the diverse ways in which OP is conceptualized and embedded within national and cultural frameworks.

### Research dedicated to OP

Few countries reported research focused primarily on OP (see Table 3 for country-specific OP research themes, organizations, networks, and policies with national remit). Instead, OP was more commonly examined within broader research contexts, including education, health, and urban planning. Research was drawn from multiple disciplines, such as sociology, geography, and public health, and typically positioned OP as a secondary focus or as a contributor to other outcomes, particularly PA. In some countries, including Slovenia, OP was described as under-researched and frequently subsumed under broader concepts such as recreation, limiting the identification of play-specific studies.

**Table 3.** Country-specific OP research themes, organizations, networks, and policies with national remit

Ctry.	Research	Organization focus (type)	Networks	Policies			
				Specific	Health	Education	Other
AL (JJ)	+ H&W + MB + N&E + E&L	+ Education (SO)	None	None	+ PA (2018-2025) (A)	+ Pre-University Education (L)	+ Children (2017-2023) (A) + Environment (2019-2030) (A)
AT (TM)	+ MB + N&E + E&L + EA (risky play)	+ Child-friendly cities (SO) + Youth representation (SO)	+ FitSport Austria	None	+ PA (2024) (A) + PA (2023) (D)	None	None
HR^a^ (SSa)	+ H&W + MB + N&E + E&L + HR&P + EA (risky play)	+ Public health (GB)	+ OMEP Croatia	None	+ Public health (2030) (D)	+ Physical education (2017) (D)	None
DK (PBe, MBø)	+ H&W + MB + N&E + E&L	+ Outdoor life (‘friluftsliv’) (SO) + Nature (GB) + Nature education (SO) + Design (SO) + Non-competitive PA (‘idræt’) (SO)	+ Nature and Children—Denmark + Healthy Cities Network	+ Outdoor life (‘friluftsliv’) (2015)	+ Public health (2018) (D) + Children’s public health (2019) (A)	+ ECEC (2023) (D)	+ Access to nature (L)
EE (EM, GML)	+ MB + E&L	+ PA in schools (SO) + Children’s outdoor life (SO) + PA (SO) + Culture (GB)	+ Participative Budgeting	None	+ PA (A) + PA center (2024–2027) (D) + Public health (A)	+ Health in ECEC (R) + Health in school (R)	None
FI (MK, SSi)	+ MB + E&L + EA (risky play)	+ Child Welfare (SO) + Outdoor life (C) + Environmental Education (SO) + Nature (SO) + PA (SO)	+ Play Day Network + National early childhood network	None	+ PA (A) + PA (2023-2025) (D)	+ ECEC (D)	+ Nature recreation 2023 (D)
FR (BB, GC)	+ H&W + N&E + E&L	+ Science (SO) + Education (SO) + National Parks (SO) + Outdoor school (SO)	+ National Network for Environmental Education + Outdoor School Network + Early childhood association for sustainable development practices + Network for Nature Pedagogies + Let’s go outside France	None	+ PA (2021) (A)	+ ECEC (2021) (D) + School (2020-2025) (D)	+ Child-friendly spaces (2024) (A) + Environment (2021-2030) (D)
GR (KR)	+ E&L + N&E	+ Play (SO)	None	None	None	+ ECEC (D)	None
HU (TC)	+ MB + E&L	+ Green ECEC (SO) + Bird life (SO) + Green schools (SO)	None	None	None	+ ECEC (A) + School (D)	None
IE (TM, MBe)	+ H&W + MB + N&E + E&L + HR&P	+ Children, Equality, Disability, Integration and Youth (GB) + Public health (GB) + Outdoor life (GB) + Childhood (SO) + Early Childhood Ireland (SO) + Play (SO) + Play-friendly cities (SO)	+ Get Ireland Active + Let’s Play Ireland Government Initiatives + Children’s Research Network of Ireland + P4Play	+ Play (A) + Recreation Young People (A) + Review of Play and Recreation (2024) (A) + Outdoor Recreation (2023-2027) (A)	+ PA (2016) (A) + PA (2022) (D) + Public health/obesity (2016-2025) (A, D) + MB (2024) (A)	+ ECEC (A) + ECEC (D) + School design guidelines (2013) (L) + School Design guidelines (2017) (L)	+ Children and Young People (2023-2028) (A) + Youth Plan (A) + Children 2019-2028 (A)
KSV (AK)	None	None	+ Towards the Mountain project	None	None	+ Non-formal education (D)	None
ME (SP)	+ MB + EA (interactions with sex, interactions with climate)	+ National Parks (SO) + Outdoor life (SO) + Youth Outdoor life (SO) + Environment (SO)	+ Montenegro Outdoor Coalition + Montenegro Youth Network + Balkan Green Network + South East European Youth Network + Eco-schools Network Montenegro	None	+ Public health (2016-2030) (D) + Wellbeing and PA (2017-2021) (D) + PA (2018-2021) (D)	+ Environmental Education (2020-2030) (D)	None
NL (MBl)	+ H&W + MB + N&E + E&L + HR&P + EA (neurobiological processes, risky play, inclusive play)	+ OP (SO) + Disability (SO) + Youth (SO) + Inclusion (SO) + Play, leisure, and nature (SO) + Play and PA (SO) + Outdoor Education (SO)	+ International Play Association—Dutch branch + Space for Youth (Platform Ruimte voor de Jeugd) + Inclusive Play Network (Samenspeelnetwerk)	None	+ PA (D, A) (2023-2026) + Public Health (D, A)	None	None
NO (EBS)	+ H&W + MB + N&E + E&L + EA (interactions with sex, interactions with ethnicity)	+ Outdoor life (‘friluftsliv’) (SO) + ECEC (SO) + Science (SO) + Non-competitive PA (SO) + Tourism (SO) + Children’s outdoor life (SO)	+ Norwegian Network for Physical Education in ECEC training + Network for Nature- and Farm Kindergartens + Network for Research on Physical Education and Sport in School	+ Outdoor life (‘friluftsliv’) (A)	+ Public health (A) + PA (2020-2029) (D)	+ ECEC (D) + School (D)	+ Access to nature (L)
PL (KM)	+ H&W + MB + E&L	+ ‘GratoSfera Foundation’ (SO) + ‘Fundacja Dzieci i Natura’ (SO) + ‘Fundacja Czas na Las’ (SO) + ‘Rezerwat Dzikich Dzieci’ (SO) + Environment (GB) + PA (SO) + ‘Fundacja SEMAFOR’ (SO) + Outdoor Education (SO)	+ Project Space for Children + Coalition for Environmental Education + Polish Association for Physical Education (PSWF)	None	+ Public health (2021-2025) (A)	None	+ Rural Development (2014-2020) (A) + Regional Development (2030) (D)
SK (PBa)	+ H&W + MB + E&L	+ Outdoor life (SO) + Experiential education (SO) + Scouting (SO) + Youth PA (SO) + Youth community (SO) + Youth outdoor life (SO) + Innovative education (SO) + Environmental education (SO)	+ The Club of Slovak Tourists + Association of Children’s Forest Clubs + TAKE ME OUT + The Spiral + The Pine Tree	None	+ PA (2022–2026) (A) + After School Education (A)	+ ECEC (A) + Physical education (D) + Outdoor Education (A)	None
SI (SAM, NB)	+ MB + N&E + E&L + EA (risky play)	+ PA (SO) + Outdoor life (SO) + School and after-school (SO)	+ Active Healthy Kids Slovenia + ‘Prezneca project’ + Adventurous Play and Outdoor LEarning + Program Going out for Health (‘Ven za zdravje’)	None	None^a^	None^a^	+ Provision of safe playgrounds and green space (L)
ES^b^ (SVS, JBS)	+ H&W + MB + N&E + E&L + HR&P	+ International Play Association—Spain (SO) + Education and Development Foundation (SO) +Technological Institute for Chidlren’s products & leisure (SO) + Meniños Foundation (SO) + Association of Wellbeing and Development (SO) + Growing Up Playing Foundation (SO) + Fundación Crecer Jugando (SO) + Spanish Association of Toys Manufacturers (SO)	+ Observatory of Children’s Play in Spain	None	None	+ School breaks (A)	None
SE (ML, CDN)	+ H&W + MB + N&E + EA (measuring OP)	+ Outdoor life (‘friluftsliv’) (SO)	+ Children’s built environments + The Swedish network for green school grounds and outdoor learning	+ Outdoor life (‘friluftsliv’) (2012) (A, D)	+ PA (A) + PA (A)	None	+ Access to nature (L)
UK/GB England and Northern Ireland (ML, HD, MB)	+ H&W + MB + N&E + E&L + EA (risky play, socioemotional processes)	+ Play (SO) + OP and Learning (SO) + PA (SO) + Children (SO)	+ Free Play Network + Playday + Dream Networks + Playful Communities + Children’s Play Information Service + British Educational Research Association + Play Commission + Children’s Play Policy Forum + UK Play Safety Forum	None	+ Public health (2021) (A) + Healthy Environments (A)	+ ECEC (D)	+ Rural development (2018) (D)
UK/GB Wales (ML, HD, MB, MM)	+ H&W + MB + N&E + E&L + EA (risky play, socioemotional processes)	+ Play (SO) + OP and Learning (SO) + PA (SO) + Children (SO)	+ Playful Childhoods Wales + Networks listed under England and Northern Ireland	+ Right to play (2011) (L) + Play sufficiency (2012) (R) + Play (A)	+ Child health (A)	+ Whole school approach (A) + School (D)	+ Rural development (2018) (D)
UK/GB Scotland (AJ)	+ H&W + MB + N&E + E&L + HR&P + EA (socioemotional processes)	+ Play (SO) + OP and Learning (SO) + OP and Learning (C) + PA (SO) + Children (SO) + Outdoor life (C, PA) + Education (SO) + ECEC (GB) + Public health + Nature (SO)	+ Scotland’s National OP and Learning Position Statement + Networks listed under England and Northern Ireland	+ Right to play (L) + Play Sufficiency (R) + Play (D) + Nature play (A)	+ PA (A) + PA (D)	+ ECEC (A) + ECEC inspectorate (R)	+ Rural development (2018) (D)

a HR, Croatia/Hrvatska,

b ES, Spain/España.

EA, Emerging Areas; E&L, Education & Learning; H&W, Health & Wellbeing; HR&P, Human Rights & Policy; MB, Movement Behaviours; N&E, Nature & Environment.

Across the represented countries (with the UK counted as one country), five research themes were most frequently reported: movement behaviours (*n *= 17), education and learning (*n *= 17), nature and the environment (*n *= 13), health and wellbeing (*n *= 12), and human rights and policy (*n *= 5). Examples within these themes included children’s PA during outdoor recess, the role of nature in early learning, children’s preferred play environments, comparisons between OP and screen time in relation to mental health, and inclusive play spaces. Emerging areas of interest included sociocultural and climatic correlates, risky play, socioemotional and neurobiological processes, and measurement of OP. These patterns reveal both the system’s current capabilities and areas for expansion.

### Practice

#### Organizations

Most countries reported the presence of specialist organizations with a national remit that sought to promote OP, either as a primary objective or as part of a broader set of aims. In several countries, including the Scandinavian countries, Estonia, and Slovenia, organized associations were described whose exclusive purpose was to promote and protect opportunities for outdoor life (‘friluftsliv’) and outdoor recreation within their countries.

Across countries, OP was rarely the sole focus of organizations. More commonly, it was promoted alongside related aims within specialist organizations concerned with education in broad terms (e.g. Albania, France), learning (e.g. UK), science (e.g. France), outdoor or nature education (e.g. Denmark, Poland), nature preservation (e.g. Finland, France), play- or child-friendly cities (e.g. Austria, Ireland), general or inclusive play (e.g. Greece, Ireland, the Netherlands), PA (e.g. Estonia, Poland, Slovakia), early childhood (e.g. Ireland), youth (e.g. Austria), and child welfare (e.g. Finland). While few organizations focus exclusively on OP, many integrate it into broader initiatives, demonstrating the system’s existing capacity to provide outdoor opportunities.

In several countries, charities and government bodies were also reported to support OP-related initiatives. Charitable organizations focused on outdoor life (e.g. Finland, Scotland) or on OP in connection with learning (e.g. Scotland), although these did not exclusively target OP. With the exception of an outdoor recreation–focused unit within the Irish Department of Rural and Community Development, government involvement was typically embedded within broader remits. A small number of countries reported government bodies responsible for nature or environmental protection that also addressed OP (e.g. Denmark, Poland), while in others OP appeared on the agenda of public health authorities (e.g. Croatia, Ireland).

#### Networks

Across countries, a range of networks were reported that brought together stakeholders from sectors including early childhood education and care (e.g. Finland), outdoor schools (e.g. France), eco-schools (e.g. Montenegro), environmental and outdoor education (e.g. France, Montenegro), PA (e.g. Norway, Slovakia), physical education (e.g. Poland), health (e.g. Slovenia), play (e.g. UK), and play rights (e.g. Ireland), with the shared aim of promoting opportunities for OP.

Most networks operated within national contexts, although some extended beyond country boundaries. For example, the Danish Healthy Cities Network, which forms part of WHO’s European Healthy Cities Network, promotes public health through initiatives that include outdoor life and nature. At a regional level, the Balkan Green Network supports outdoor education and sustainability across the Western Balkans, while the Southeast European Youth Network engages young people in non-formal education, including OP, through experiential learning activities across several Southeast European countries.

### Policy

Most countries reported having no policy specifically targeting OP. Only four countries—Denmark, Ireland, Sweden, and the UK—reported the presence of policies with an explicit focus on OP [14–17].

Within the UK, Scotland and Wales reported legislation that prescribed children’s play opportunities, including OP, alongside regulatory mechanisms to support implementation. In Wales, the Play Sufficiency Duty (2012) requires local authorities to assess and secure sufficient play opportunities for children and provides a detailed policy framework that is referenced by UK-wide advocacy organizations [14]. Since its introduction, Play Sufficiency Assessments have been reviewed in 2013, 2016, 2019, and 2022 [18], and the statutory guidance was refreshed in 2025 [19].

In Sweden, the Ministry of Climate and Enterprise, together with the Environmental Protection Agency and other governmental bodies, formulated national goals and actions to support opportunities for outdoor life (‘friluftsliv’) for both children and adults, including the aim that all residents should have access to nature [20]. Responsibility for implementation was assigned to a designated governmental body, and progress towards these goals was reviewed in 2015, 2019, and 2023 [17].

#### Health

Many countries reported the inclusion of OP within broader policy recommendations related to PA (e.g. Albania, Austria), public health (e.g. Estonia), and obesity prevention (e.g. Ireland). In most cases, OP was promoted as a means of supporting PA or health, with some countries, including Ireland and Norway, also reporting actionable delivery plans. For example, Norway’s national PA strategy identifies outdoor life (‘friluftsliv’) as a central approach, emphasizing access to outdoor environments and facilities and promoting outdoor activities across schools, kindergartens, and after-school settings [21].

#### Education

Many countries reported the inclusion of OP within education frameworks, particularly in early childhood education and care (e.g. Finland, Greece) and in both formal (e.g. France, Hungary) and informal schooling (e.g. Kosovo). A smaller number of policies highlighted OP in PE (e.g. Croatia) or environmental education (e.g. Montenegro). While most policies were advisory in nature, some included requirements or oversight mechanisms: for example, Estonia mandates outdoor activities through preschool health regulations, and Scotland has an agency responsible for ensuring play sufficiency in early childhood settings.

#### Other

Additional policies referencing OP focused on ensuring access to environments supportive of OP (e.g. Albania, UK), promoting public access to nature (e.g. ‘allemannsretten’ in some Scandinavian countries), and enhancing environmental planning to increase the provision of safe, play-friendly neighbourhoods (e.g. France).

## Discussion

This study provides a first comprehensive overview of OP research, practice, and policy across Europe. We found a striking diversity in how OP is understood, studied, and supported. Terminology differs widely across countries, reflecting cultural and contextual nuances in the conceptualization of OP. Research specifically focused on OP, as defined by PLaTO-Net [22], remains limited, with most studies examining it indirectly within broader themes such as play, recreation, PA, health, or education. Despite this, evidence is growing in areas including movement behaviours, learning, nature, wellbeing, and human rights. Practice is driven by a patchwork of specialist organizations across education, nature, play, and child welfare, which promote OP alongside other priorities. Policy attention is similarly uneven: few countries have policies dedicated solely to OP, yet it frequently appears within health, education, and planning frameworks. Together, these patterns suggest that while OP is widely recognized as valuable, it often remains embedded within broader agendas rather than treated as a distinct focus. Overall, terminological diversity, uneven research attention, fragmented practice, and variable policy support define the system’s current capabilities. These patterns highlight opportunities to strengthen the system and provide a foundation for coordinated action to enhance children’s OP opportunities across Europe.

### Conceptual challenges

Most European countries have long and diverse traditions of OP, yet few explicitly conceptualize these practices under that label. Instead, a variety of culturally and contextually embedded terms is used to describe activities that align with the PLaTO-Net definition [22]. This diversity underscores the ongoing need for terminological clarity—if not full consensus—which motivated the PLaTO-Net harmonization process. The present study unpacks this linguistic and cultural variation, highlighting where misunderstandings or oversights may arise.

A central debate in defining OP concerns the emphasis on play as voluntary and intrinsically motivated, which can exclude educational forms of play. Across Europe, OP is often linked to both informal and formal learning, reflecting two broad perspectives: (i) play as a tool for learning (e.g. play-based or guided play) [23], (ii) play as a learning process in itself, a perspective prevalent in countries with an ‘ethos of play’ where play transcends formal education [23–25]. Rather than privileging one view, it is important to recognize these diverse interpretations and cultural contexts to support inclusive research, policy, and practice.

We found distinct groupings of OP terminology. One cluster describes free play occurring outdoors, another refers to geographically delineated play (e.g. park play, street play, play in the meadows), often referencing landscapes particular to each country. A third cluster encompasses non-specific outdoor activities implicitly recognized as intrinsically motivated and rewarding—such as ‘friluftsliv’, outdoor recreation, or simply ‘being outside’—consistent with the definition of play adopted by Lee *et al.* [22]. Other groupings may exist, but these categories offer a preliminary topology of OP across Europe.

### Strengths and limitations

This study integrates multiple perspectives across Europe and employs of a complimentary mixed-methods approach. Nonetheless, the several limitations should be noted. First, most country-specific reports were compiled by only one to three contributors, so findings reflect individual expertise and experience. We attempted to mitigate this using Global Matrix 4.0 data, although these data may be subject to similar expert-elicitation biases. Second, despite extensive recruitment efforts, 17 countries were not represented. Finally, the review focused on national- and European-level research, practice, and policy, and did not capture initiatives limited to local jurisdictions. Viewed through a systems lens, these findings illustrate the current state of OP as shaped by the interactions of research, practice, and policy. They also point to opportunities to enhance the system’s capabilities: harmonizing terminology, developing standardized assessment tools, expanding research to address underexplored areas such as equity, climate, and social capital, and supporting policies that are actionable, monitored, and inclusive. This perspective lays the groundwork for conceptualizing OP as a dynamic, interconnected system whose effectiveness depends on the coordinated strengthening of all components.

### Implications for research, practice, and policy

This study highlights several opportunities to strengthen the European OP system. First, there is a clear need to harmonize terminology and concepts across research, practice, and policy to enable better comparative studies and international collaboration. Developing standardized, contextually adaptive assessment tools could help unify diverse approaches and consolidate OP as a distinct research area and priority for practice and policy across Europe. Second, European research on OP often addresses isolated outcomes, with limited exploration of broader connections to health, climate, environmental issues, and equity. Inequalities in OP, including the impact of colonial legacies on marginalized groups, remain under-researched. Future studies should address these gaps and examine themes such as social capital, community engagement, and ‘one health’ perspectives to better support equitable policies and inclusive practice. Third, policy attention to OP remains limited. Few countries have OP-specific policies, and while many health and education policies support OP, they are often non-binding and may not lead to meaningful action. Exemplars, such as the Welsh and Scottish models, combine legislation, oversight, guidance, evaluation, and advocacy to create a more capable and coherent OP system. While such models may inspire other jurisdictions, systematic monitoring and evaluation remain scarce, and governance and contextual differences across Europe suggest that flexible, partnership-based strategies may be more effective than one-size-fits-all solutions. Finally, the development of international, cross-disciplinary researcher and practitioner forums could enhance system coordination by:

1. Identifying priority research areas;
2. Conducting national-level studies using harmonized, standardized methods;
3. Facilitating comparative EU-level research in underexplored areas;
4. Informing Europe-wide policies and initiatives; and
5. Raising broader awareness of OP’s importance across Europe.

Such coordinated efforts could enable funding bodies to support more impactful, mission-driven projects, ultimately strengthening the research-practice-policy system and enhancing OP’s contributions to children’s health, learning, equity, and connection to nature.

## Supplementary Material

ckag040_Supplementary_Data

## Data Availability

Expert-elicited raw files are enclosed in Appendix S1. Key Points Terminological and cultural differences shape the outdoor play (OP) system, highlighting the need for harmonization and clearer, inclusive definitions. OP is often addressed indirectly through broader themes like recreation, education, or health, limiting its recognition as a standalone right. Few countries have OP-specific policies, and most are non-binding, constraining the system’s ability to support play. Diverse organizations promote OP, but coordination across sectors is limited. Public health policy should prioritize OP by integrating it into binding frameworks that recognize its role in promoting physical, mental, and social wellbeing.
