# Supplementary material for: Outdoor play in Europe: terminology and state of research, practice, and policy
Source: Eur J Public Health. 2026 Mar 28;36(2):ckag040. doi: 10.1093/eurpub/ckag040 (PMC13032894; doi:10.1093/eurpub/ckag040)
Supplement: ckag040_Supplementary_Data [file ckag040_supplementary_data.pdf]

Country: Albania

Name of assessor: Juel Jarani (Act4Edu Center)

Date of assessment: 13.11.2024

Data form for outdoor play research, practice, and policy in Europe

This data form on outdoor play (OP) is to be completed in reference to the past 10 years and the country whom the assessor is representing.

OP is defined as a form of play that takes place outdoors [1]. Play is defined as voluntary engagement in an activity that is fun and/or rewarding and usually driven by intrinsic motivation. Please observe that this can refer to both children and adults. This document should not exceed two pages in total.

TERMINOLOGY

The term OP might not be directly or perfectly translated to the language/languages spoken in the country in question. Please name and describe terms that are used in country in question that could be conceptualised as or include outdoor play. Please also comment on how physical activity relates to these terms.

In Albania, "outdoor play" (lojërat në natyrë) typically refers to children engaging in physical activities and games outside, whether in parks, playgrounds, or schoolyards. This form of play is widely valued for its role in promoting physical health, social interaction, and cognitive development. Outdoor play in Albania often includes traditional games, group sports, or free play, and is a key aspect of childhood that supports the overall growth and well-being of children.

Outdoor play in Albania has a rich history rooted in community and tradition. Historically, Albanian children engaged in a variety of traditional games passed down through generations, such as **"dy gurë"** (two stones), **"kukafshehti"** (hide and seek), and **"lufta e kalamajve"** (children's battle), often played in village squares or open fields. These games, which required little to no equipment, fostered creativity, teamwork, and social skills.

During the socialist era (1945-1991), the Albanian government emphasized collective sports and physical education. Schools and youth organizations encouraged outdoor activities as a way to build health, discipline, and solidarity. Playgrounds and sports fields were developed in urban areas, and children had structured times for outdoor play, often monitored by teachers and community leaders.

In recent decades, urbanization and modernization have influenced outdoor play in Albania, especially in cities, where open spaces are sometimes limited. However, schools and communities still promote outdoor play, and traditional games are being preserved through cultural events and educational programs. Efforts are ongoing to ensure children have safe, accessible spaces for outdoor play, recognizing its importance for physical health, social skills, and cultural continuity.

[1] Lee, Eun-Young, Louise de Lannoy, Lucy Li, Maria Isabel Amando de Barros, Peter Bentsen, Mariana Brussoni, Lindsay Crompton, et al. "Play, Learn, and Teach Outdoors—Network (PLaTO-Net): Terminology, Taxonomy, and Ontology." *International Journal of Behavioral Nutrition and Physical Activity* 19, no. 1 (June 15, 2022): 66. https://doi.org/10.1186/s12966-022-01294-0.

In Albania, several terms and expressions are commonly used to refer to *outdoor play*, each emphasizing different aspects of playing outdoors:

1. **Lojëra jashtë** – A general term meaning "games outside."
2. **Aktivitete fizike në natyrë** – Translates to "physical activities in nature," often used to emphasize physical engagement.
3. **Lojëra në ajër të hapur** – Means "games in open air," highlighting the outdoor environment.
4. **Veprimtari argëtuese në natyrë** – Translates to "entertainment activities in nature," focusing on the fun aspect of outdoor play.
5. **Lojëra në fushë** – Literally "games in the field," often used in school or community settings to refer to organized games or sports outside.

These terms are all familiar ways to describe *outdoor play* in Albania, with slight variations that fit different contexts, from informal free play to structured physical activities.

RESEARCH

Name active OP researchers and describe their research focus.

Juel Jarani (children, health, physical activity, education. Genta Nicaj (children, activities, play, sport, Adri Kasmi (kids, playgrounds, outdoor activities, Anisa Nurja (kids, sport, activities physical, strength).

Describe scope of scientific publications and reports on OP stemming from country in question (please include references of seminal publications and publications that relate to gender, race, class, or climate).

Anne Purdon (2016): A comparison of free time activity choices of third culture kids in Albania and children in the UK, Education 3-13, DOI: 10.1080/03004279.2016.1218523 To link to this article: http://dx.doi.org/10.1080/03004279.2016.1218523

PRACTICE

Name and describe organizations (e.g., Outdoor Play Canada) that support and promote OP.

 Save the Children Albania – Though not an individual researcher, the organization has collaborated with local researchers and educators on projects like *Shkollat për Shëndetin* (Schools for Health), which emphasizes the role of outdoor play in promoting children's health in schools.

▯ **ACT for Education Center (Qendra ACT4EDU)** – An educational organization in Albania that collaborates with experts to develop and implement programs on physical activity and outdoor play for children.

Name and describe networks (e.g., PLaTO-Net) that support and promote OP.

NA

POLICY

Name and describe national policy documents that support OP.

**National Strategy for Children (2017-2023)**<br>This strategy is a key document outlining the Albanian government's commitment to child welfare and development. It emphasizes the importance of creating environments where children can play, learn, and grow. The strategy includes measures to improve access to quality education, healthcare, and recreational opportunities, with an emphasis on outdoor play and physical activity.<br><br>**Law No. 69/2012 on Pre-University Education in the Republic of Albania**<br>This law governs the education system in Albania and includes provisions on physical education and outdoor activities for children and youth. The law acknowledges the importance of outdoor play and physical exercise as part of the school curriculum.<br><br>**National Strategy for the Promotion of Physical Activity (2018-2025)**<br>This national strategy aims to increase physical activity among all age groups, with a specific focus on children and youth. It acknowledges outdoor play as a key factor in promoting active lifestyles from an early age.<br><br>**The National Strategy for the Environment (2019-2030)**<br>This strategy focuses on environmental sustainability and aims to improve the quality of life for citizens, including children, by enhancing green spaces and public parks.

Name and describe fundings bodies that support OP.

**1. Save the Children Albania**<br>**2. United Nations Children's Fund (UNICEF)**<br>**3. European Union (EU) – EU Delegation to Albania**<br>**4. World Bank**<br>**5. Norwegian Embassy in Albania**

OTHER

Country: Austria

Name of assessor: Thomas Morgenthaler

Date of assessment: 15. November 2024

Data form for outdoor play research, practice, and policy in Europe

This data form on outdoor play (OP) is to be completed in reference to the past 10 years and the country whom the assessor is representing.

OP is defined as a form of play that takes place outdoors [1]. Play is defined as voluntary engagement in an activity that is fun and/or rewarding and usually driven by intrinsic motivation. Please observe that this can refer to both children and adults. This document should not exceed two pages in total.

TERMINOLOGY

The term OP might not be directly or perfectly translated to the language/languages spoken in the country in question. Please name and describe terms that are used in country in question that could be conceptualised as or include outdoor play. Please also comment on how physical activity relates to these terms.

Spiel (Play, noue)<br>*Spielen* (playing, the act of play,)<br>*Spielen im Frein* or draußen spielen (playing outdoors)<br>Freispiel (free play, that is guided by the child(ren) more commonly used in pre-school setting)<br>*Bewegung* (physical activity)<br>*Bewegung im Freien* (being physical activive outdoors)<br>Bewegungsaktiv (active physical activity)

RESEARCH

Name active OP researchers and describe their research focus.

Only one researcher was identified that has actively focused on outdoor play in an Austrian context:<br>Dr. Rosa Diketmüller (University of Vienna, one project on outdoor play in preschool setting focus on gender difference in preference and physical activity)<br><br>These researcher do not focus on outdoor play but are active and their research topics partially also includes outdoor play:<br>Dr Sabine Hennig (Paris-Lodron-Universität Salzburg, Children and youth-friendly cities, participation of children in urban environments, open green spaces,<br><br>Raphaela Kogler (University of Vienna, participation of children in urban environments, visual and arts-based methods, Children friendly cities)

[1] Lee, Eun-Young, Louise de Lannoy, Lucy Li, Maria Isabel Amando de Barros, Peter Bentsen, Mariana Brussoni, Lindsay Crompton, et al. “Play, Learn, and Teach Outdoors—Network (PLaTO-Net): Terminology, Taxonomy, and Ontology.” *International Journal of Behavioral Nutrition and Physical Activity* 19, no. 1 (June 15, 2022): 66. https://doi.org/10.1186/s12966-022-01294-0.

Describe scope of scientific publications and reports on OP stemming from country in question (please include references of seminal publications and publications that relate to gender, race, class, or climate).

Two research studies where identified which examines outdoor play in school yards with a focus on gener and physical activity

1. Diketmüller, R., Berghold, B., Förster, B., Frommhund, E., Witzeling, J., Studer, H., Gungl, B., Hemmelmeier-Händel, B., & Schirl, S. (2007). *Schulfreiräume und Geschlechterverhältnisse [School yard and Geneder]*. University of Vienna & tilia-büro für landschaftsplanung. https://www.eduresearch.at/fileadmin/DAM/Gegenstandsportale/Gender_und_Bildung/Dateien/ABSCHLUSSBERICHT.pdf
2. Diketmüller, R., & Gungl, B. (2017). Kindergarten—Freiräume für Mädchen und Buben [Kindergarten—Open spaces for girls and boys]. *Playground@Landscape*, *2*, 28–36.

Conference paper on free play opportuntites in preschool setting (indoor and outdoor setting)

1. Majcen, J., Steinmann, R., Taslimi, N., & Mittlböck, K. (2020). Spielraum Freispiel. Mehr als eine Spielerei? *Open Online Journal for Research and Education*.

Studies which examines youth experiences in participatory practices in design of urban environments:

1. Hennig, S., Vogler, R., Waltl, D., & Schötz, T. (2024). Zur Situation und Verbesserung von Partizipation junger Menschen in der Stadtplanung. *Standort*. https://doi.org/10.1007/s00548-024-00936-8

Studies and book chapters which focus on children's viewpoints on urban environments and participatory practices of creating spaces for and with children in urban environments

1. Kogler, R. (2018). Kinderräume erkunden: Partizipative Stadtforschung und -planung mit Kinder. *Informationen Zur Raumentwicklung*, *2*.
2. Kogler, R. (2019). Räume für Kinder – Räume der Kinder: Typologien urbaner Kinderräume. *Stadtentwicklung*, 11–14.
3. Kogler, R., & Lindinger, K. (2022). Kinder und Ihre Städte: Lebensräume zwischen gebauter und sozialer Welt. In *Kinderrechte in Deutschland: Interdisziplinäre Perspektiven auf Errungenschaften und Herausforderungen kinderrechtlicher Arbeit in Deuschland* (pp. 139–150). kopaed.

Austrian physical activity guideline

Austrian guidelines for physical activity recommend unstructured play, "free play" in outdoor environments for children under 10 years:

1. Ring-Dimitriou, S., Windsperger, K., Felder-Puig, R., Kayer, B., Zeuschner, V., & Lercher, P. (2020). Österreichische Bewegungsempfehlungen für Frauen während der Schwangerschaft und danach, für Kinder im Kindergartenalter und für Kinder und Jugendliche [Austrian Physical Activity Recommendations for Women during and after Pregnancy, Children of Kindergarten age, Children, and Adolescents]. *Das Gesundheitswesen*, *82*(S 03), S177–S183. https://doi.org/10.1055/a-1191-4060

Projects of participatory designing school yards in one of Austrian counties:

1. Wolf, R., & Haubenhofer, D. (2018). *„Jetzt ist für alle etwas dabei" Endbericht zur Begleitstudie*

Förderaktion Spielplätze und Schulhöfe in Bewegung. Niederösterreichische Landesregierung & Hochschule für Agrar- und Umweltpädagogik. https://www.naturimgarten.at/files/content/4.%20GARTENWISSEN/Studien%2C%20Umfragen%20und%20Diplomarbeiten/STUDIE%20SPIELPLAETZE%20UND%20SCHULHOEFE%20IN%20BEWEGUNG.pdf

Studies on children's perspectives and their needs mention outdoor play:

1. Zartler, U., Kromer, I., & Zuccato-Doutlik, L. M. (2018). *Was alle Kinder brauchen! Sichtweisen von Mädchen und Buben zu einem kindergerechten Warenkorb [What children need! Perspectives from girls and boys for a child-friendly shopping basket]*. University of Vienna & Austrian National Youth Council. https://bjv.at/wp-content/uploads/2018/05/studie_was-alle-kinder-brauchen.pdf
   - A recommendation from the children was to create attractive spaces for play and leisure free of charge in public open spaces, including public spaces, parks, playgrounds and nature and with play equipment
   - Furthermore, playing, especially with peers, was described to be linked to children's experience of happiness

PRACTICE

Name and describe organizations (e.g., Outdoor Play Canada) that support and promote OP.

Nationalwide:

- Kinderfreudliche Gemeinde (child-friendly cities and communities imitative)

https://unicef.at/mitmachen/kinderfreundliche-gemeinden/

Kinderrecht netzwerk [Network for children rights], but no advocacy work on outdoor play

Bundes Jungend Vertretung [Federal youth representation], has currently a poster campaign on children's rights what includes one poster on article 31 the right to play.

Name and describe networks (e.g., PLaTO-Net) that support and promote OP.

National

- No national associations are known which actively advocates for outdoor play.
- FitSport Austria a national network which coordinates and offers opportunities to by physical active, what includes some outdoor play however it is organizes and do not offer free play

Regional some examples from cities:

City of Graz offers play opportunities such as closing of streets and summer opportunities that focus on play through a mobile bus: https://www.graz.at/cms/beitrag/10323087/7752318/Spielstrassen_Kaiserfeldgasse_und_Prankergasse.htmlhttps://www.graz.at/cms/beitrag/10230110/7752318/Spielmobile_Spiel_und_Spass_in_Parks.html

PLAY streets in Vienna: The city of Vienna closes certain streets down and creates play streets which stay permanent during some summer weeks, some of this closed street also provides play materials such as loos parts drawing, balls, and some of this play streets offer water play for hot summer months. Free of charge

Supervised play opportunities in some city parks are offered in Vienna. Free of charge

POLICY

Name and describe national policy documents that support OP.

No national policy document was identified which directly refers to outdoor play. Currently Austria has no play policy documents on national or regional level. Below I list some policy documents which refere partly to outdoor play.

Other National policy documents

- BMKÖDS, & BMSGPK. (2024). *Nationaler Aktionsplan Bewegung 2024 [Austrian's national action plan for physical activity]*. Bundesministerium für Kunst, Kultur, öffentlichen Dienst und Sport [Ministry of the Arts, Culture, the Civil Service and Sport] & Bundesministerium für Soziales, Gesundheit, Pflege und Konsumentenschutz [Federal Ministry of Social Affairs, Health, Care and Consumer Protection]. https://www.bmkoes.gv.at/dam/jcr:54f1f9cc-e62d-4d8f-aa1d-075b4dafa44f/NapB_Publikation_2024.pdf
  - Physical activity guidelines from Austria which mention outdoor play, but it is not a main focus, this plan endorse more structured and adult controlled activates, what
  - For example, following goals are described
    - Aim 3: create activities
    - Aim 8: Improve the framework conditions for an increased physical activity-promoting orientation of educational institutions
    - Aim 11: increase of active mobility: Home to school and out-of-school activities via foot, scooter, bike, skateboard, Principles: Network of bikeable and walkable streets, the proximity of relevant spaces for active mobility
    - Aim 12: Physical activity in Nature and Landscape
    - Aim13: Provide all population groups with spaces for exercise, **play** and sport, or make these accessible and usable, and take the exercise aspect into account in all types of buildings and facilities a

- BMSGPK. (2024). *Gesundheitsförderungsstrategie im Rahmen der Zielsteuerung Gesundheit*. Bundesministerium für Soziales, Gesundheit, Pflege und Konsumentenschutz.
  - Austrian health promotion strategy does not directly refer to outdoor play, but one strategy is to increase healthy living conditions and way of life with a focus on children and youth. This strategy talks about the relevance of priorities being physically active as well as nutrition.

- Ministry of Social Affairs, Health, Care and Consumer Protection. (2023). *Nationaler Aktionsplan zur Umsetzung der Europäischen Garantie für Kinder [European child guarantee: Austia's national action plan]*. https://www.sozialministerium.at/Themen/Soziales/Soziale-

Themen/Europäische-Garantie-für-Kinder.html

- Austrian action plan for Children's guarantee describes Austria's current and future action plan to ensure aspects of health and includes physical activity. Part of this action plan gives some understating of actions relevant to outdoor play, outdoor play opportunities in the environment (living and public spaces), as well as being active. Important is that these are not the main focus of this document it covers much more, and it does not directly refer to outdoor play.

Regional policy documents or guidleins

Vienna City Administration. (2020). *Die Wiener Kinder und Jugendstrategie 2020 – 2025 [The Vienne children and youth strategy 2020—2025]*. Magistrat der Stadt Wien. https://junges.wien.gv.at/wp-content/uploads/sites/48/2020/09/The-Vienna-Children-and-Youth-Strategy.pdf

The **Children and Youth Strategy** of the city of Vienna outlines actions the city aims to achieve by 2025. These actions were shaped by incorporating children's perspectives through consultation processes with NGOs, city institutions, and experts. The following actions are directly related to outdoor play:

- **Expand opportunities for outdoor play and physical activity** in public spaces.
- **Increase "play along the way" opportunities** in public spaces.
- **Address temperature reduction measures** to improve comfort in outdoor areas.
- **Provide diverse seating options** that are available in various heights and feature colorful designs.
- **Plant more trees that offer fruit and herbs** in public spaces.
- **Involve children in the development of new play opportunities.**
- **Ensure play areas are publicly accessible.**
- **Install additional lighting** at playgrounds and multi-use games areas (MUGAs).
- **Provide ball rental stations** for easy access to sports equipment.
- **Create more thematic, water, and adventure playgrounds** and introduce motor skill courses for physical activity.
- **Add inclusive play equipment** that is usable for children with disabilities.

Name and describe fundings bodies that support OP.

None where identified.

OTHER

Country: CROATIA

Name of assessor: SANJA ŠALAJ

Date of assessment: 12th November, 2024.

Data form for outdoor play research, practice, and policy in Europe

This data form on outdoor play (OP) is to be completed in reference to the past 10 years and the country whom the assessor is representing.

OP is defined as a form of play that takes place outdoors [1]. Play is defined as voluntary engagement in an activity that is fun and/or rewarding and usually driven by intrinsic motivation. Please observe that this can refer to both children and adults. This document should not exceed two pages in total.

TERMINOLOGY

The term OP might not be directly or perfectly translated to the language/languages spoken in the country in question. Please name and describe terms that are used in country in question that could be conceptualised as or include outdoor play. Please also comment on how physical activity relates to these terms.

Direct translation of outdoor play in Croatian language is "igra na otvorenom". It is used mostly to describe activities (static and active) of children (not really for adults) outside of the house in nature or playgrounds. It is not the most frequent term used. Other terms used for OP in Croatian language are "boravak na otvorenom" (outdoor stay), "boravak na svježem zraku" (stay in fresh air) used mainly for children being outside and playing regardless of season or weather that is thought to be beneficial for overall development and immune system. "Aktivnosti na otvorenom" (outdoor activities) or "Aktivnosti u prirodi" (activities in nature) are terms for different activities of children or adults performed for fun and recreation in outside environment. They include but are not restricted to physical activities. All terms are used to describe the enjoyment of being outside in fresh air and in exploring nature. In education system terms "učenje u prirodi" and "škola u prirodi" are used as well, with addition of learning through exploring.<br><br>More related to physical activity terms that are used in Croatian language are "Tjelesna aktivnost u prirodi" (physical activity in nature), "Tjelesno vježbanje u prirodi" (Physical exercise in nature), "Tjelovježba u prirodi" (Physical exercise in nature), "Aktivni boravak u prirodi" (Active stay in nature), "Aktivno provođenje vremena u prirodi" (Actively spending time in nature).

RESEARCH

Name active OP researchers and describe their research focus.

Edita Rogulj (children, outdoor education), Adrijana Višnjić Jevtić (children, education, risky play), Sandra Kadum (children, outdoor education), Petra Poljak (Katavić)(outdoor education), Ivana Visković (children, play, risky play), Dunja Anđić (children, education, outdoor), Marija Karačić (education, children, play), Višnja Rajić (children, education, play, children rights), Kornelija Mrnjaus (children rights, play), Hildegarde Auf-Franić and associates (arhitecture, planning), Danijel Jurakić (physical activity, active

[1] Lee, Eun-Young, Louise de Lannoy, Lucy Li, Maria Isabel Amando de Barros, Peter Bentsen, Mariana Brussoni, Lindsay Crompton, et al. "Play, Learn, and Teach Outdoors—Network (PLaTO-Net): Terminology, Taxonomy, and Ontology." *International Journal of Behavioral Nutrition and Physical Activity* 19, no. 1 (June 15, 2022): 66. https://doi.org/10.1186/s12966-022-01294-0.

play), Sanja Šalaj (children, active play, physical activity), Maroje Sorić (physical activity, health), Sanja Musić Milanović (public health), Maja Lang Morović (public health)

Describe scope of scientific publications and reports on OP stemming from country in question (please include references of seminal publications and publications that relate to gender, race, class, or climate).

There is not much OP research in Croatia. Most of the research is focused on the attitudes of teachers and educators in early and primary school education towards play and risky play and being outside. In the professional works, they are focused on the design of work in the educational system with children in the open space.
Another group of researchers (experts) studies the external environment from an architectural and agronomic perspective, where they are also involved in the environmental planning of kindergartens and children's playgrounds in Croatia.
Other researchers are from the field of public health and physical activity and investigate physical activity and inactivity in the context of health, and observe movement in general and movement outdoors (free active play), where play outside is mostly associated with low-intensity physical activity. Most of the research involves children.
Research on OP amount in various age groups, type of OP preffered, gender/climate/regional differences, correlates or influence of OP on development/skills/health is lacking.

Selection of references in english language:

1. Pedišić, Ž., Strika, M., Matolić, T., Sorić, M., Šalaj, S., Dujić, I., Rakovac, M., Radičević, B., Podnar, H., Greblo Jurakić, Z., Jerković, M., Radašević, H., Čvrljak, J., Petračić, T., Musić Milanović, S., Lang Morović, M., Krtalić, S., Milić, M., Papić, A., Momčinović, N., Mišigoj-Duraković, M., Heimer, S., & Jurakić, D. (2023). Physical Activity of Children and Adolescents in Croatia: A Global Matrix 4.0 Systematic Review of Its Prevalence and Associated Personal, Social, Environmental, and Policy Factors. Journal of Physical Activity and Health, 20(6), 487-499. https://doi.org/10.1123/jpah.2022-0500
2. Musić Milanović, S., Križan, H., Pavičić, L., Lang Morović, M. & Baić, M. (2021) Physical activity risk behaviour in school-aged children is associated with overweight and maternal characteristics. Kinesiology : international journal of fundamental and applied kinesiology, 53 (2), 309-317 doi:10.26582/k.53.2.14.
3. Lang Morović, M., Križan, H., Bukal, D., Musić Milanović, S. (2019). Healthy living, volunteers in parks: opportunity for health and physical activity in the community throughout the lifespan // Book of abstracts of the Congress „Healthy & Active Children 2019, 651, 2.
4. Visković, I., Sunko, E., Mendeš, B. (2019). Children's Play—The Educator's Opinion. *Education Sciences* 9(4):266. https://doi.org/10.3390/educsci9040266
5. Kadum, S:, Šegon, E. (2023). Spending Time Outdoors: The Educator and the Child. Human research and rehabilitation, 13 (2023), 2; 292-301. doi: 10.21554/hrr.092312
6. Sandseter, E. B. H., Cordovil, R., Hagen, T. L., & Lopes, F. (2019). Barriers for Outdoor Play in Early Childhood Education and Care (ECEC) Institutions: Perception of Risk in Children's Play among European Parents and ECEC Practitioners. Child Care in Practice, 26(2), 111–129. https://doi.org/10.1080/13575279.2019.1685461 (CROATIAN PARENTS IN SAMPLE)
7. Karačić, M., Kadum, S., & Vukašinović, A. (2024). Importance of children's play: the Croatian experience of future ECEC teachers. European Early Childhood Education Research Journal, 1–14. https://doi.org/10.1080/1350293X.2024.2359550
8. Katavić, P (2019). Educators organization and planning for outdoor play in kindergarten // CARN-ALARA 2019 - Imagine Tomorrow: Practitioner Learning for the Future, 17-19 October 2019, Split,

Croatia - Book of Abstracts / Bognar, Gazibara, Simel Pranjić (Eds). Osijek: Filozofski fakultet Sveučilišta Josipa Jurja Strossmayera u Osijeku, 2019. pp. 114-115
9. Višnjić Jevtić, A., Sadownik, A. R., & Halavuk, A. (2021). Early Childhood Education teachers' attitudes towards risky play as developed through teacher education and impeded by safety procedures. A report from Croatia. Journal of Adventure Education and Outdoor Learning, 22(2), 135–147. https://doi.org/10.1080/14729679.2021.1894952

PRACTICE

Name and describe organizations (e.g., Outdoor Play Canada) that support and promote OP.

Croatian Institute of Public Health is the central public health institution in the Republic of Croatia. Through scientific research and health policies it is oriented to disease prevention and health promotion. Within the National program Living Healthy, this institution focuses on promoting outdoor play of children in playgrounds and parks around Croatia (activity Volunteers in Park).

Name and describe networks (e.g., PLaTO-Net) that support and promote OP.

OMEP Croatia is a component of the international Organization Mondiale pour l'Education Préscolaire. The goal of OMEP Croatia is to promote and ensure optimal conditions for all children, their development, learning and happy growing up in families, institutions and the community. At the conferences they organize and in the published proceedings, they promote the right to play for children and OP for the benefit of the child's health and emotional, social, cognitive and physical development.

POLICY

Name and describe national policy documents that support OP.

In the National Development Strategy of the Republic of Croatia until 2030, the national program Live Healthy (Živjeti zdravo) is described. National program is implemented by the Croatian Institute of Public Health, which aims to encourage a healthy and active lifestyle. Part of the National Living Healthy program is the activity Health and the Environment: Volunteers in the Park (Volonteri u parku), which promotes a healthy lifestyle, prosocial behavior and preservation of the environment among children, young people and adults in the community. The mentioned project involves the promotion and organization of intergenerational socializing events outdoor, in parks and children's playgrounds throughout Croatia.

Planning of OP is a part of national documents of Ministry of Education (2017): „Curriculum for the subject of Physical education for primary schools and high schools in the Republic of Croatia" and „National document of the Physical and health area of the curriculum".

Name and describe fundings bodies that support OP.

-

OTHER

-

Country: Denmark

Name of assessor: Mads, Peter, and Laerke

Date of assessment:

Data form for outdoor play research, practice, and policy in Europe

This data form on outdoor play (OP) is to be completed in reference to the past 10 years and the country whom the assessor is representing.

OP is defined as a form of play that takes place outdoors [1]. Play is defined as voluntary engagement in an activity that is fun and/or rewarding and usually driven by intrinsic motivation. Please observe that this can refer to both children and adults. This document should not exceed two pages in total.

TERMINOLOGY

The term OP might not be directly or perfectly translated to the language/languages spoken in the country in question. Please name and describe terms that are used in country in question that could be conceptualised as or include outdoor play. Please also comment on how physical activity relates to these terms.

The literal translation of outdoor play is 'udeleg' in Danish which conceptualizes any type of play outdoors, including guided play where an adult might initiate or scaffold the play for children. It often, but not necessarily, involves non-sedentary activities. Denmark has a strong tradition for outdoor play in early childhood care. Many outdoor or nature kindergartens ('naturbørnehaver', 'skovbørnehaver', 'naturbørnehaver') and outdoor pedagogics ('udepædgogik') include a lot of (guided and free) outdoor play. Danish kindergartens generally have a strong tradition for valuing play (including play) for the sake of play in itself and for the whole child, but also can sometimes be used more instrumentally (with and without adult-led scaffolding) as a means to support the children's development and school readiness.<br><br>The term 'friluftsliv' (outdoor life) likewise denotes voluntary and intrinsically motivated behaviors that take place outdoors and are fun and/or rewarding in some way. In this manner, there seems to be a considerable overlap with the definition of outdoor play – however, friluftsliv always occurs in natural environments, specifically – the extent of their 'naturalness' is continuously debated. It often, but not necessarily, involves non-sedentary activities.<br><br>In Denmark, there is a distinction between physical activity with no competitive aim ('idræt') and physical activity with a competitive aim (sport). Here, it would seem that 'idræt' that takes place outdoors ('udendørs' or 'ude') would align more closely with the concept of outdoor play.<br><br>Denmark has a rich tradition for providing fun and exciting publicly available outdoor playgrounds ('legepladser'). Amongst these exist sub types such as nature playgrounds ('naturlegepladser') and loose parts/trash playgrounds 'skrammellegepladser', both of which have a plentitude of (risky) play affordances.<br><br>We exclude the term education outside the classroom ('udeskole') because this mostly, in a Danish context, has curricular aims.

[1] Lee, Eun-Young, Louise de Lannoy, Lucy Li, Maria Isabel Amando de Barros, Peter Bentsen, Mariana Brussoni, Lindsay Crompton, et al. "Play, Learn, and Teach Outdoors—Network (PLaTO-Net): Terminology, Taxonomy, and Ontology." *International Journal of Behavioral Nutrition and Physical Activity* 19, no. 1 (June 15, 2022): 66. https://doi.org/10.1186/s12966-022-01294-0.

RESEARCH

Name active OP researchers and describe their research focus.

Lærke Mygind (children, friluftsliv, health, learning, and development), Peter Bentsen (children, friluftsliv, health, learning, and development), Mads Bølling (children, friluftsliv, health, learning, and development), Thea Toft Amholt (teenagers, playgrounds), Jasper Schipperijn (playgrounds, green space, friluftsliv, physical activity), Ina Specht (outdoor kindergartens, child development and health), Jan Arvidsen (children's places and use of outdoors, friluftsliv), Søren Andkjær (friluftsliv, health, learning), Erik Mygind (friluftsliv, health, learning, development), Ole Lund (outdoor play), Peder Agger (nature, parks, ethics, friluftsliv), Niels Ejbye-Ernst (nature kindergartens, pedagogy, development), Dorte Stokholm (nature kindergartens, pedagogy, development), Birgitte Theilman (nature kindergartens, pedagogy, development)

Describe scope of scientific publications and reports on OP stemming from country in question (please include references of seminal publications and publications that relate to gender, race, class, or climate).

Mygind L, Hartmeyer R, Kjeldsted E, Mygind E, Bentsen P. Viden om friluftslivs effekter på sundhed – resultater fra en systematisk forskningsoversigt. København: Friluftsrådet, 2018. (cited by Danish Health Authority)

Friluftslivsrapporter
Kom med ud
Baselineundersoegelse

PRACTICE

Name and describe organizations (e.g., Outdoor Play Canada) that support and promote OP.

Friluftsrådet (The Danish Outdoor Council) - an umbrella organization inspiring towards more outdoor life through projects, public affairs, labeling schemes and other campaigns.

Naturstyrelsen (The Danish Nature Agency) - an agency in "Ministeriet for Grøn Trepart" that takes care of governmental nature areas and creates new nature all around the country.

Naturvejledere - Naturvejledning Danmark. "Skaber forståelse og indsigt i dansk natur. Har forskellige projekter, fx "Krible Krable" i samarbejde med DR Ramasjang der opfordrer dagsinstitutioner, skoler, SFO'er og børnefamilier til at komme ud i naturen og på smådyrsjagt

Gerlev Center for leg og bevægelse - viden og erfaring indenfor leg og bevægelse. Formidler til virksomheder. Skoler, institutioner og arbejdspladser. Arbejder politisk for at udbrede legende og glædesfyldt bevægelse. (på hjemmesiden står ikke noget om udenfor/udendørs)

Name and describe networks (e.g., PLaTO-Net) that support and promote OP.

Center for Children and Nature

Sund By Netværket

POLICY

Name and describe national policy documents that support OP.

Forebyggelsespakkerne (the Prevention Packages) 2018 by the Danish Health Authority – use of nature and friluftsliv for mental health promotion.

Børn og unges sundhed og trivsel, Anbefalinger til kommuner (Children and Youth's Health and Wellbeing, recommendations for municipalities) 2019 by the Danish Health Authority – use of nature and friluftsliv for mental health promotion.

Sundhedsstyrelsen – godt at vaere ude, det gode foraeldreskab

Den Styrkede Pædagogiske Læreplan (The pedagogical curriculum) 2023 - one of the six curriculum themes focuses on "nature, outdoor life and natural phenomena" and play is foundational across all themes.

Allemandseje? – strand, groenne arealer (dog ogsaa privat skov), offentlige parker og skove, baggaarde

Name and describe fundings bodies that support OP.

Friluftsraadet
15. juni
Legofonden?
Just Human – More Play puljen. Supports construction of playgrounds.

OTHER

Country: England and Wales and Northern Ireland

Name of assessor: Mark Leather

Date of assessment: 1st November 2024

Data form for outdoor play research, practice, and policy in Europe

This data form on outdoor play (OP) is to be completed in reference to the past 10 years and the country whom the assessor is representing.

OP is defined as a form of play that takes place outdoors [1]. Play is defined as voluntary engagement in an activity that is fun and/or rewarding and usually driven by intrinsic motivation. Please observe that this can refer to both children and adults. This document should not exceed two pages in total.

TERMINOLOGY

The term OP might not be directly or perfectly translated to the language/languages spoken in the country in question. Please name and describe terms that are used in country in question that could be conceptualised as or include outdoor play. Please also comment on how physical activity relates to these terms.

Outdoor play is term that is used widely in the UK. There is sometimes a tendency to equate outdoor play with play in nature but it includes any kind of outdoor play; there is significant work in England (and across the UK) focused on children's street play for example. Active outdoor play is not so commonly used, although it is assumed that outdoor play is related to physical activity but physically active outdoor play and sedentary outdoor play are rarely distinguished in policy or practice. Risky play and adventurous play are used interchangeably and are accepted as linked to outdoor play, primarily because concerns about risk are a clear barrier to outdoor play.

RESEARCH

Name active OP researchers and describe their research focus.

**Many researchers in England are interested in play. It is rare for a researcher to focus on outdoor play specifically. Instead researchers tend to focus on play in education, outdoor learning, play in public spaces/use of space, play in a more general sense which includes outdoor play.**<br><br>**Dr Lorna Arnott**<br>Affiliation: University of Strathclyde (Scotland, but collaborates widely in England)<br>Specialization: Digital play, early childhood, and innovative play practices. While her primary focus is not exclusively on outdoor play, her research covers a broad spectrum of play-based learning.<br><br>**Professor Helen Bilton**<br>Affiliation: University of Reading

[1] Lee, Eun-Young, Louise de Lannoy, Lucy Li, Maria Isabel Amando de Barros, Peter Bentsen, Mariana Brussoni, Lindsay Crompton, et al. "Play, Learn, and Teach Outdoors—Network (PLaTO-Net): Terminology, Taxonomy, and Ontology." *International Journal of Behavioral Nutrition and Physical Activity* 19, no. 1 (June 15, 2022): 66. https://doi.org/10.1186/s12966-022-01294-0.

Specialization: Outdoor play, early childhood education, and the role of outdoor spaces in child development. She has written extensively on the importance of outdoor learning and the educational potential of outdoor play environments.

Professor Pat Broadhead
Affiliation: Leeds Beckett University
Specialization: Play and pedagogy, children’s social and emotional development through play, and the role of play in early years education.

Professor Helen Dodd
Affiliation: University of Exeter
Specialization: Child mental health, focusing on how adventurous play can help prevent mental health issues in children. Dodd's work suggests that adventurous play, which involves activities where children experience some level of fear or uncertainty, can promote emotional resilience and reduce symptoms of anxiety and depression.

Tim Gill
Affiliation: Independent scholar, honorary research fellow at the University of Exeter
Specialization: Risk and play, outdoor environments, and children's interactions with public spaces. Although not based full-time at a university, he collaborates frequently with academics and policy makers on play and risk in outdoor settings.

Dr Jan White
Affiliation: Independent consultant but works closely with many UK universities and research groups.
Specialization: Early childhood outdoor play. Her research focuses on the benefits of outdoor play for young children’s development and wellbeing. She has also published multiple resources on designing outdoor spaces for play.

Professor Elizabeth Wood
Affiliation: University of Sheffield
Specialization: Play, pedagogy, and curriculum in early childhood education, and she has contributed significantly to understanding how play influences children's learning and development. Elizabeth Wood's work is internationally recognized, particularly in exploring how children integrate digital and traditional forms of play and how these interactions inform pedagogy and educational practices.

Prof Alison Stenning
Affiliation: University of Newcastle
Specialization: focused on how play on streets reshapes our everyday and emotional geographies. Particularly how play articulates with our everyday relationships, material and emotional, to streets and the people on them, and how play has the potential to support relationships.

Dr Wendy Russell
Affiliation: Independent researcher and visiting fellow University of Gloucester
Specialization: research focuses on children’s play, in particular on the politics of space and on policy.

Prof Peter Kraftl
Affiliation: University of Birmingham
Specialization: Geographies of children’s space, urban design to support healthy development including play.

PEDAL Centre, University of Cambridge – focuses on play in education, not outdoor play specifically, but with some interest in outdoor play in this context.

Describe scope of scientific publications and reports on OP stemming from country in question (please include references of seminal publications and publications that relate to gender, race, class, or climate).

As outlined above, scientific publications rarely focus on outdoor play specifically. Papers focus on play in education, forest schools as a route to providing outdoor play, trends in and correlates of children's play (including outdoor play), spaces for play.

Baines, E., & Blatchford, P. (2023). The decline in breaktimes and lunchtimes in primary and secondary schools in England: Results from three national surveys spanning 25 years. *British Educational Research Journal*, *49*(5), 925-946.

Dodd, H. F., Nesbit, R. J., & FitzGibbon, L. (2023). Child's play: examining the association between time spent playing and child mental health. *Child Psychiatry & Human Development*, *54*(6), 1678-1686.

Dodd, H. F., FitzGibbon, L., Watson, B. E., & Nesbit, R. J. (2021). Children's play and independent mobility in 2020: results from the British Children's Play Survey. *International journal of environmental research and public health*, *18*(8), 4334.

Garden, A., & Downes, G. (2023). A systematic review of forest schools literature in England. *Education 3-13*, *51*(2), 320-336.

Gessiou, G., & Mart, M. (2023). Outdoor play and learning practices from a comparative case study perspective. *Journal of Childhood, Education & Society*, *4*(3), 338-353.

Gill, T. (2021). *Urban playground: How child-friendly planning and design can save cities*. Riba Publishing.

Goodhall, N., & Atkinson, C. (2020). An exploratory case study: Children's perceptions of play access in two schools (England and Wales). *Educational & Child Psychology*, *37*(4), 37-52.

Hattingh, L. (2024). Time to play, time to think: meaningful moments in the forest. *European Early Childhood Education Research Journal*, *32*(1), 22-33.

Hesketh, K. R., & Dodd, H. F. (2023). More play and fewer screens: a way to improve preschoolers' mental health? Cross-sectional findings from the British Preschool-children's Play Survey. *The Lancet*, *402*, S49.

Josephidou, J. (2020). A gendered contribution to play? Perceptions of Early Childhood Education and Care (ECEC) practitioners in England on how their gender influences their approaches to play. *Early years*, *40*(1), 95-108.

Kemp, N., & Josephidou, J. (2023). Creating spaces called hope: the critical leadership role of owner/managers in developing outdoor pedagogies for infants and toddlers. *Early Years*, *43*(3), 641-655.

Leather, M., Harper, N., & Obee, P. (2021). A pedagogy of play. Reasons to be playful in post-secondary education. *Journal of Experiential Education, 44*(3) 208–226.

Mackinder, M. (2024). A bird's eye view: comparing young children's play in Forest School in England with Forest Kindergarten in Denmark. *Education 3-13*, *52*(5), 718-735.

Nikiforidou, Z., & Jones, J. (2023). Preschoolers' intuitive probabilistic thinking during outdoor play. *Statistics Education Research Journal*, *22*(2), 2-2.

Parker, R., & Al-Maiyah, S. (2022). Developing an integrated approach to the evaluation of outdoor play settings: rethinking the position of play value. *Children's Geographies*, *20*(1), 1-23.

Rixon, A., Lomax, H., & O'Dell, L. (2019). Childhoods past and present: Anxiety and idyll in reminiscences of childhood outdoor play and contemporary parenting practices. *Children's geographies*, *17*(5), 618-629.

Rixon, A., Lomax, H., & O'Dell, L. (2019). Childhoods past and present: Anxiety and idyll in reminiscences of childhood outdoor play and contemporary parenting practices. *Children's geographies*, *17*(5), 618-629.
Russell W, Stenning A. Kerbs and curbs, desire and damage: an affirmative account of children's play and being well during the COVID-19 pandemic. *Social & Cultural Geography* 2023, **24**(3-4), 680-698.
Smith, T. A., Pitt, H., & Dunkley, R. A. (Eds.). (2022). *Unfamiliar landscapes: young people and diverse outdoor experiences*. Palgrave Macmillan.

PRACTICE

Name and describe organizations (e.g., Outdoor Play Canada) that support and promote OP.

Most of these organisations advocate for children's play or represent stakeholders within the play sector. There are fewer examples where organisations are actively delivering play at a national level. Instead play is delivered at a local level through schools, public play spaces, playschemes, holiday and before/after school clubs etc.

International Play Association – England branch https://ipaengland.org/
The International Play Association England (IPA England) is a leading member of a global child rights advocacy network dedicated to promoting a child's right to play. We recognise the importance of ensuring that children are supported in their fundamental right to play freely and openly without fear of judgement or criticism. This is especially important in the UK, where we believe there is a lack of understanding around the significance of play and its impact on children's overall well-being.

Play England https://www.playengland.org.uk/
Play England's vision is for England to be a country where everybody can fully enjoy their right to play throughout their childhood and teenage years, as set out in the UN Convention on the Rights of the Child Article 31 and the Charter for Children's Play.

Play Wales https://play.wales/
Play Wales is the national organisation for children's play. It is a charity that provides advice, support and guidance for all those in Wales who have a concern or responsibility for any environment where children and young people might play. Play Wales upholds children's right to play and believes that freely chosen play is critically important in the healthy development of all children and young people. All children are entitled to quality play provision within their communities and Play Wales works strategically to achieve this goal on their behalf.

PlayBoard Northern Ireland https://www.playboard.org/
PlayBoard is the lead agency for the promotion and development of play in Northern Ireland. The organisation works strategically to lobby and advocate for the child's right to play.

OPAL – Outdoor Play and Learning https://outdoorplayandlearning.org.uk/
Our vision is that every child in every school has an amazing hour of high-quality play every day – with no exceptions. If one child is not enjoying playtimes, then things still need improving.
We want every school to plan for, resource and evaluate the quality of their play provision as if it were an important human right, essential to all aspects of children's development and a source of joy and happiness that every child can access because it is all of these things.

SkillsActive http://skillsactive.com/
SkillsActive is the sector skills council for active leisure and learning, and can advise on training and employment opportunities. www.skillsactive.com

The Childrens Alliance https://childrensalliance.org.uk/
Better Starts, Brighter Futures. Giving children the best start in life – Most people don't realise how much a child's first five years will impact their physical and mental wellbeing for life. We want to change that. *The Power Of Play: Building A Creative Britain*

Federation of Sports and Play Associations https://sportsandplay.com/
FSPA is the national trade body representing manufacturers, wholesalers and distributors in the sports and play industry.

Playwork Foundation - https://playwork.foundation/
Represents playworker in England.

Association of Play Industries (API) - https://www.api-play.org/
The Association of Play Industries (API) is the lead trade association for the UK play sector. Its member companies are the UK's leading providers of outdoor playground equipment; indoor play equipment and safety surfacing for schools, local authorities, parish councils, leisure attractions, holiday parks, housing developments, hospitality venues and commercial enterprises.

Name and describe networks (e.g., PLaTO-Net) that support and promote OP.

Free Play Network http://www.freeplaynetwork.org.uk/index.html
An independent voice for play that advocates for the right of children to play and for play to be free for all. They also offer advice on designing inclusive play spaces in schools, public spaces, and parks.

Play Day https://www.playday.org.uk/
Playday is a campaign that highlights the importance of play in children's lives and focuses on a different issue each year. Playday is coordinated by Play England, Play Scotland, Play Wales, and PlayBoard Northern Ireland.

Playful Childhoods Wales https://playfulchildhoods.wales/
Playful Childhoods is a Play Wales campaign. Our mission. To help adults give children a happy, healthy childhood through play. Every day.

Dream Networks https://dreamnetworks.co.uk/
An organization that designs inclusive play spaces for children in collaboration with schools, businesses, and community groups.

Playful Communities
The Playful Communities website provides information, advice and resources for individuals, local community groups, third sector organisations and others who are developing play provision in their local neighbourhood.

Children's Play Information Service (CPIS)
A service that can help people find their nearest local play network or association. CPIS is is a resource that provides information on play, including factsheets and reading lists for students. CPIS is part of the

National Children's Bureau Library and Information Service and is funded by the Department for Culture, Media and Sport (DCMS) and the Big Lottery Fund.

British Educational Research Association
Special Interest Group: Nature, Outdoor Learning and Play
https://www.bera.ac.uk/community/nature-outdoor-learning-and-play

Play Commission
https://www.centreforyounglives.org.uk/play-commission

POLICY

Name and describe national policy documents that support OP.

Department for Education (DfE) - Early Years Foundation Stage (EYFS) Framework

- The EYFS framework outlines standards for the learning, development, and care of children from birth to age five. It emphasises the importance of outdoor play as a key part of a child's learning and development.
- The 2021 update of the EYFS framework includes specific references to the benefits of outdoor activities for physical development and encourages early years providers to make good use of outdoor spaces.

https://www.gov.uk/government/publications/early-years-foundation-stage-framework--2

Public Health England (PHE) – "Improving children and young people's mental health and wellbeing: A whole school and college approach" (2021)

- This guidance document highlights the role of physical activity, including outdoor play, in promoting children's mental health and wellbeing. It encourages schools to provide regular opportunities for outdoor play as part of a holistic approach to mental health.

https://assets.publishing.service.gov.uk/media/614cc965d3bf7f718518029c/Promoting_children_and_young_people_s_mental_health_and_wellbeing.pdf

Department for Environment, Food & Rural Affairs (DEFRA) – "25 Year Environment Plan" (2018)

- DEFRA's 25 Year Environment Plan includes strategies to connect people with the environment, particularly through initiatives promoting outdoor play and learning in natural spaces. It supports creating opportunities for children and young people to engage with nature, recognizing the positive impacts on their health and education.

https://assets.publishing.service.gov.uk/media/65fd713d65ca2f00117da89e/CD1.H_HM_Government_A_Green_Future_Our_25_Year_Plan_to_Improve_the_Environment.pdf

Wales: Play Sufficiency Duty (2012)

- While not specific to England, this devolved strategy (Play Sufficiency Duty in Wales) provides a detailed policy framework that are referenced by UK-wide advocacy groups. The Play Sufficiency Duty requires local authorities in Wales to assess and secure sufficient play opportunities for children.

https://www.gov.wales/sites/default/files/publications/2019-07/wales-a-play-friendly-country.pdf

Name and describe fundings bodies that support OP.

The National Lottery Community Fund (UK-wide)

- This fund provides grants for community-led projects that bring people together and improve community cohesion. It operates under different names across the UK: the National Lottery Community Fund England, the National Lottery Community Fund Wales, and the National Lottery Community Fund Northern Ireland.
- The fund supports projects that create or improve spaces for outdoor play, promote children's well-being through outdoor activities, and increase access to nature. Examples include playground renovations, forest school programs, and community gardens.

https://www.tnlcommunityfund.org.uk/

The People's Postcode Lottery (UK-wide)

- The People's Postcode Lottery distributes funds raised by lottery players to a variety of charities and community groups across the UK, including those focused on environmental sustainability, community cohesion, and youth development.
- Funds projects that enhance children's experiences in the outdoors, support outdoor education, and develop community-based outdoor play initiatives. This can include creating natural play spaces or supporting outdoor learning programs.

https://www.postcodelottery.co.uk/

Sport England

- A government body that provides funding to increase participation in sport and physical activities across England. While its main focus is on sports, Sport England supports initiatives that encourage active outdoor play, especially for young people.
- Funds projects that encourage physical activity and sport, including outdoor recreation. This includes creating or refurbishing outdoor play areas and encouraging informal play that leads to an active lifestyle.

https://www.sportengland.org/

Natural Resources Wales (Wales-specific)

- This public body is responsible for managing natural resources sustainably in Wales. It funds projects that enhance access to nature and promote outdoor activities, including play.
- Supports outdoor play and educational projects that engage communities with the environment. This includes funding for forest schools, outdoor adventure programs, and the development of play spaces in natural settings.

https://naturalresources.wales/?lang=en

Community Foundation Northern Ireland

- This independent foundation provides funding to support community development and well-being across Northern Ireland. It supports a range of initiatives that promote physical and mental health, community engagement, and youth development.
- Funds community-driven projects that create safe and accessible outdoor play spaces, promote outdoor learning, and encourage physical activity among children and young people.

https://communityfoundationni.org/

Children in Need (UK-wide)

- A charity that provides grants to projects helping children and young people facing disadvantage.
- Funds projects that use outdoor play and activities to promote physical and mental well-being. This can include adventure play, outdoor learning, and therapeutic play for vulnerable children.

https://www.bbcchildreninneed.co.uk/

OTHER

Country: Estonia

Name of assessor: Evelin Mäestu & Getter Marie Lemberg

Date of assessment: 1th of November, 2024

Data form for outdoor play research, practice, and policy in Europe

This data form on outdoor play (OP) is to be completed in reference to the past 10 years and the country whom the assessor is representing.

OP is defined as a form of play that takes place outdoors [1]*. Play is defined as voluntary engagement in an activity that is fun and/or rewarding and usually driven by intrinsic motivation. Please observe that this can refer to both children and adults. This document should not exceed two pages in total.*

TERMINOLOGY

The term OP might not be directly or perfectly translated to the language/languages spoken in the country in question. Please name and describe terms that are used in country in question that could be conceptualised as or include outdoor play. Please also comment on how physical activity relates to these terms.

The translation of outdoor play in Estonia is "õues mängimine" which conceptualizes any type of play taking place outdoors, including forests, playgrounds, recreational centres and other outdoor environments. Outdoor play usually refers to active activities, but free play does not really control what activities children do, so the activities can also be more sedentary activities. In kindergarten children in Estonia usually go outside at least once a day (usually before lunch), but they often go outside in the afternoon as well. So-called "outdoor kindergartens" ("õuelastehoid" "õuelasteaed") are not popular in Estonia, but some have been created recently.<br><br>There are many playgrounds ("mänguväljak") in Estonia, especially in larger cities, where children can play and be active outdoors.<br><br>Schools have begun to pay more attention to outdoor recess ("õuevahetund"), based on which many schools have renovated their schoolyards to provide students with more play and activity opportunities during outdoor recess. Schools have also changed their school day structure to include a longer at least 30min outdoor recess in their daily schedule. For the most part, schoolyards are also open after school hours and children can play there at any time. At the same time, kindergarten playgrounds are not open for everybody to use in Estonia.

RESEARCH Name active OP researchers and describe their research focus.

Getter Marie Lemberg, Evelin Mäestu – outdoor recess, active schoolyards, outdoor play

[1] Lee, Eun-Young, Louise de Lannoy, Lucy Li, Maria Isabel Amando de Barros, Peter Bentsen, Mariana Brussoni, Lindsay Crompton, et al. "Play, Learn, and Teach Outdoors—Network (PLaTO-Net): Terminology, Taxonomy, and Ontology." *International Journal of Behavioral Nutrition and Physical Activity* 19, no. 1 (June 15, 2022): 66. https://doi.org/10.1186/s12966-022-01294-0.

Describe scope of scientific publications and reports on OP stemming from country in question (please include references of seminal publications and publications that relate to gender, race, class, or climate).

Lemberg, G.M.; Riso, E.-M.; Fjørtoft, I.; Kjønniksen, L.; Kull, M.; Mäestu, E. School Children's Physical Activity and Preferred Activities during Outdoor Recess in Estonia: Using Accelerometers, Recess Observation, and Schoolyard Mapping. Children 2023, 10, 702. https://doi.org/10.3390/children10040702

Masing, M. Supporting factors and barriers of work in an outdoor day-care centre according to its teachers. Master thesis. Tartu, 2017. https://dspace.ut.ee/server/api/core/bitstreams/23c6f923-9cc1-4551-ba9b-600c6312d2a9/content

PRACTICE Name and describe organizations (e.g., Outdoor Play Canada) that support and promote OP.

Schools in Motion ("Liikuma Kutsuv Kool") - science-driven education innovation program in Estonia. Schools in Motion is implementing a "whole-school approach", i.e creating possibilities to be physically active throughout the school day, including recess, lessons, school travel, physical education lessons, extra-curricular activities, school events and teachers' activities, both indoors and outdoors. One of the program's main focus recently has been outdoor recess and active play during outdoor recess.

NGO Kids Outdoors ("MTÜ Lapsed Õue") – NGO Kids Outdoors is a community of families who are enthusiastic about nature, outdoor learning, autonomous and unstructured free play and sustainability (reuse, upcycling, gardening). Adventure playgrounds, which are spaces for youth designed to provide various opportunities for building, creating, communicating, moving, and playing, offer kids the challenges they need. Such spaces might be integrated into schoolyards, youth and community centres, but can also be built temporarily for festivals and other events. An adventure playground ('kolahoov' in Estonian) is a playscape made by children for themselves. Its essential elements are autonomous free play and loose parts. Adventure playgrounds support creativity, initiative, autonomy, and social skills.

The Competence Centre for Physical Activity ("Liikumisharrastuse kompetentsikeskus") – include initiating and supporting development and research-based intervention programs, collecting and introducing best practices from the world, empowering and assessing the organisations operating in the field, and developing and managing the training system designated for the field. The mission of this organization is to comprehensively increase the physical activity of Estonians. One of its main focuses recently has also been encouraging more activities outdoors. A recent project of the Competence Centre for Physical Activity included providing various basketball courts and soccer fields all across the country with boxes with balls that everyone can use for free when playing at the specific court or a field.

Ministry of Culture ("Kultuuriministeerium") – continuously supports comprehensive physical activity and outdoor movement

Name and describe networks (e.g., PLaTO-Net) that support and promote OP.

Participative Budgeting ("Kaasav eelarve") – program that gives citizens opportunities to participate in budget designing process in multiple cities and municipalities. Every year citizens can submit ideas that should be done within the city or the municipality. Every year more and more ideas are related to improving outdoor play and outdoor activities opportunities for citizens. For example, many schoolyards and playgrounds have been renovated from "participative budgeting".

POLICY Name and describe national policy documents that support OP.

Ministry of Culture – The concept of physical activity? ("Liikumisharrastuse kontseptsioon") – One of the goals of "The concept of physical activity" is to promote physical activity among everybody, by doing that Ministry of Culture is supporting construction and renovation of various infrastructure related to encouraging and supporting physical activity, including infrastructure that promotes outdoor play and activities.

Ministry of Culture and the Competence Centre for Physical Activity - Development plan of the competence centre for physical activity 2024–2027. The main goal of the development plan is to encourage physical activity and create projects and infrastructure that supports increased physical activity, including outdoor play and activities.

Ministry of Social Affairs – "Health protection requirements for health promotion and agenda in preschool institutions" provides that in favorable weather conditions, as many children's activities as possible must take place outdoors. In addition, the child must spend 1-2 times outside every day, depending on the weather conditions.

Ministry of Social Affairs – "Health and safety regulations for school schedule and organisation" states that extended school day program („pikapäevarühm") activities should take place for at least an hour after the school day and depending on the weather conditions outdoors or indoors.

National Health Insurance provider ("Tervisekassa") - carrying out various announcements and campaigns, eg. "Õpime õuemänge!" (We learn outdoor games),

Name and describe fundings bodies that support OP.

Different Ministries fund building and renovating infrastructure (for example schoolyards, playgrounds etc), however there are no specific funding bodies that support OP

OTHER

Country: Finland

Name of assessor: Marjaana Kangas & Signe Siklander

Date of assessment: 24th of October, 2024

Data form for outdoor play research, practice, and policy in Europe

This data form on outdoor play (OP) is to be completed in reference to the past 10 years and the country whom the assessor is representing.

OP is defined as a form of play that takes place outdoors [1]. Play is defined as voluntary engagement in an activity that is fun and/or rewarding and usually driven by intrinsic motivation. Please observe that this can refer to both children and adults. This document should not exceed two pages in total.

TERMINOLOGY

The term OP might not be directly or perfectly translated to the language/languages spoken in the country in question. Please name and describe terms that are used in country in question that could be conceptualised as or include outdoor play. Please also comment on how physical activity relates to these terms.

The literal translation of outdoor play is 'ulkoleikki' in Finnish which conceptualizes any type of play taking place outdoors, including forests, play parks, recreational centres and other outdoor environments. Several scientific studies on outdoor play have been conducted in Finland, particularly from the perspectives of early childhood education, sports sciences, and environmental education.

Finland has a strong tradition for outdoor play in early childhood education (ECEC) and primary education, and many outdoor or nature kindergartens ('ulkopäiväkoti', 'luontopäiväkoti') are actively running. For instance, *luontopäiväkoti* programs in Finland often align with the philosophy of forest ECE centres, where children spend a large portion of their day outdoors, exploring and interacting with nature as part of their development and learning process. In addition, adventure education ('seikkailukasvatus') used in primary and applied in higher education in Finland often incorporates elements of play in outdoor activities.

In Finland, outdoor play is afforded by many municipalities that maintain a wide range of outdoor playgrounds ('ulkoleikkipuisto') and nature-based play areas. Finland is known for integrating outdoor play into children's daily lives, with playgrounds serving as key spaces for promoting physical activity and well-being. In addition, physical activity and wellbeing is also promoted by providing outdoor playgrounds ('liikuntaleikkipuisto') for adults and elderly people.

RESEARCH Name active OP researchers and describe their research focus.

Suvi Määttä, Reetta Lehto, Ray Carola, Hanna Konttinen, Nina Sajaniemi, Maijaliisa Erkkola, Eva Roos: physically active outdoor play in preschool context
Signe Siklander (previously Pirkko Siklander and Pirkko Hyvönen): children's outdoor play and risky play, affordances for play, outdoor play in education
Marjaana Kangas: outdoor play, outdoor playful learning
Ilkka Ratinen: adventure education and outdoor learning
Pia Sjöholm, Gunilla Eklund, Petra Fagerlund: outdoor education and play

[1] Lee, Eun-Young, Louise de Lannoy, Lucy Li, Maria Isabel Amando de Barros, Peter Bentsen, Mariana Brussoni, Lindsay Crompton, et al. "Play, Learn, and Teach Outdoors—Network (PLaTO-Net): Terminology, Taxonomy, and Ontology." *International Journal of Behavioral Nutrition and Physical Activity* 19, no. 1 (June 15, 2022): 66. https://doi.org/10.1186/s12966-022-01294-0.

Describe scope of scientific publications and reports on OP stemming from country in question (please include references of seminal publications and publications that relate to gender, race, class, or climate).

Gilbertson, K., Bates, T., Siklander, P., & Ewert, A. (2022). *Outdoor Education: Methods and Strategies*, 2nd edition. USA: Human Kinetics.
Hyvönen, P. & Kangas, M. (2007). From bogey mountains to funny houses: Children's desires for play environment. *Australian Journal of Early Childhood* (AJEC), 32(3), 39–47. https://doi.org/10.1177/183693910703200307
Hyvönen, P. (2008). Teachers' perceptions of boys' and girls' shared activities in the school context: towards a theory of collaborative play. *Teachers and Teaching: Theory and Practice,* 14(5–6), 391–409. https://doi.org/10.1080/13540600802571312
Hyvönen, P. (2011). Play in the school context? The perspectives of Finnish teachers. *Australian Journal of Teacher Education (AJTE).* 36 Iss. 8, Article 5. Available at: http://ro.ecu.edu.au/ajte/vol36/iss8/5
Sjöblom, P., Eklund, G., & Fagerlund, P. (2021). Student teachers' views on outdoor education as a teaching method–two cases from Finland and Norway. Journal of Adventure Education and Outdoor Learning, 23(3), 286–300. https://doi.org/10.1080/14729679.2021.2011338

PRACTICE Name and describe organizations (e.g., Outdoor Play Canada) that support and promote OP.

Mannerheim League for Child Welfare ('Mannerheimin Lastensuojeluliitto') plays a role in promoting the well-being of children through outdoor play and physical activity.
The Outdoor Association of Finland ('Suomen Latu') – This organization promotes outdoor activities, well-being, and physical activity in natural environments.
The Finnish Association for Environmental Education ('Ympäristökasvatusjärjestö FEE Suomi') works to increase environmental awareness and education, often through nature-based learning and activities. It runs the Green Flag ('Vihreä lippu') program, which promotes environmental education and outdoor learning, often involving playful engagement with the environment.
The Finnish Nature League (Luontoliitto) focuses on environmental conservation and nature education, organizes outdoor activities that teach young people about the environment through direct interaction with nature. They emphasize the importance of outdoor play and learning for environmental stewardship.
The National Sports Council's ('Valtion liikuntaneuvosto') primary role is to promote physical activity and sports, also emphasizing outdoor activities in nature as part of a healthy lifestyle. The council provides recommendations to government bodies on promoting physical activity through outdoor and nature-based environments.
Some sustainable and nature-based programs are running:
'Metsämörri' is a program, created by the Finnish Outdoor Association, is a widely used outdoor learning method in Finnish early childhood education. It combines storytelling, environmental education, and outdoor play, where children explore forests and nature.
The Green Flag ('Vihreä lippu') program is part of the international Eco-Schools initiative, focusing on sustainability and environmental education in schools and early childhood education settings. Outdoor play activities in the Green Flag program often involve hands-on experiences like gardening, recycling games, and creative activities that reuse materials, which help children develop a connection to nature while learning sustainable practices.

Name and describe networks (e.g., PLaTO-Net) that support and promote OP.

Play Day Network ('Leikkipäivä') – This network encourages families and communities to organize local events promoting free play, including outdoor play in natural environments. It is supported by Mannerheimin Lastensuojeluliitto (MLL), Suomen Latu, and several other organizations to enhance the significance of play for child development. https://leikkipaiva.fi/

National early childhood network (‘Kansallinen varhaiskasvatusverkosto’). This network focuses on early childhood education and often includes aspects of outdoor play and nature-based activities in its developmental goals for children.

POLICY Name and describe national policy documents that support OP.

Finland has a long tradition of incorporating outdoor activities into school life, with school days structured to include regular breaks for outdoor play in the school yard. This unstructured play during recess (‘ulkovälitunti’) is a key element in supporting children's well-being and development, reflecting the Finnish education system's holistic approach to learning. In addition, outdoor playgrounds and nature-based play areas are often used for curriculum-based learning to create environments that promote children's play, physical activity, and well-being.
The National Core Curriculum for Early Childhood Education and Care (ECEC) emphasizes outdoor play as a crucial part of daily activities in early childhood settings. In basic education, schools are encouraged to use the outdoors as a learning environment, fostering connections to nature and promoting physical activity.
Local Municipalities are often key funders for playgrounds, parks, and outdoor activity areas.

Name and describe fundings bodies that support OP.

The following funding bodies have actively supported projects involving OP:
Academy of Finland (‘Suomen Akatemia’) funds research projects that explore children’s play in natural environments, focusing on developmental psychology, environmental education, and public health, which include studies and initiatives on outdoor play and learning.
Ministry of the Environment (‘Ympäristöministeriö’) supports projects that promote outdoor and nature-based activities, particularly those aiming to foster sustainable practices and a connection to nature among children and youth.
Sitra – Finland's innovation fund, occasionally funds projects promoting sustainable lifestyles and environmental education, which can include nature-based play and outdoor activities for children and youth.
The Outdoor Association of Finland (‘Suomen Latu’) provides partial funding and extensive support for local initiatives that promote outdoor play and recreation in nature, often in collaboration with municipalities and other organizations.
The Foundation of Maj and Tor Nessling funds focusing on environmental education and interaction with nature, supporting research on how outdoor environments benefit learning and well-being, especially among children.
Kone Foundation (‘Koneen Säätiö’) has supported interdisciplinary and innovative projects that explore the connection between nature and well-being. Some funded projects involve outdoor learning environments and promote playful interaction with natural spaces.
Veikkaus, a government-run gaming company, channels part of its profits into public interest activities, including sports, youth activities, and environmental projects, which sometimes include outdoor play and activities for children and families.

OTHER

Country:

Name of assessor:

Date of assessment:

Data form for outdoor play research, practice, and policy in Europe

This data form on outdoor play (OP) is to be completed in reference to the past 10 years and the country whom the assessor is representing.

OP is defined as a form of play that takes place outdoors [1]. Play is defined as voluntary engagement in an activity that is fun and/or rewarding and usually driven by intrinsic motivation. Please observe that this can refer to both children and adults. This document should not exceed two pages in total.

TERMINOLOGY

The term OP might not be directly or perfectly translated to the language/languages spoken in the country in question. Please name and describe terms that are used in country in question that could be conceptualised as or include outdoor play. Please also comment on how physical activity relates to these terms.

In France, the term *Outdoor Play (OP)* does not have a direct or formal translation. However, several related terms and concepts are used:

- **Jeu libre en extérieur** ("Free outdoor play"): This concept encourages children to explore their natural environment without structured activities or direct supervision. It is commonly used in alternative pedagogies (Montessori, Steiner).
- **École dehors** ("Outdoor school"): An educational movement growing in popularity, especially in rural areas. Children spend part of their school day outdoors, learning through nature-based activities.
- **Éducation par la nature** ("Nature-based education"): This term is used by environmental education associations such as the CPIE (Permanent Centers for Environmental Initiatives), aiming to reconnect children with nature through outdoor activities.
- **Ecole de forêt: This concept is inspired from German forest schools however pertains mainly to extra curricula activities for children during the week or on school holidays.**

Physical activity is often linked to these concepts, although it remains distinct from formal sports. Outdoor play is encouraged in schools and recreational centers to promote children's physical and mental well-being.

RESEARCH

Name active OP researchers and describe their research focus.

RESEARCH

Active OP Researchers and Research Focus:

[1] Lee, Eun-Young, Louise de Lannoy, Lucy Li, Maria Isabel Amando de Barros, Peter Bentsen, Mariana Brussoni, Lindsay Crompton, et al. "Play, Learn, and Teach Outdoors—Network (PLaTO-Net): Terminology, Taxonomy, and Ontology." *International Journal of Behavioral Nutrition and Physical Activity* 19, no. 1 (June 15, 2022): 66. https://doi.org/10.1186/s12966-022-01294-0.

- **Gillian Cante (University of Strasbourg)**: Studies the impact of free and sensory outdoor play on children's health and well-being – Her research
- Marine Jacq (University of Bretagne-Occidentale): Studies forest schools educational programmes and outdoor teaching methods
- **Nina Kleinsz (Les voies de la forêt)**: Researches nature-based education and the development of forest schools.
- Laura Nicholas (University of Paris-East Créteil): Professor (Associate) of Educational Sciences and fonder and chair of Sologna Forest School
- **Mathieu Point and Ziad Dabaja**: Co-authors of an article on the types and impacts of nature-based free play for children.
- Valérie Roy (University of Paris- 8): Director of Paris outdoor (plein air) daycare. Studies the benefits of nature for toddlers.Christophe Schnitzler (University of Strasbourg) Professor (Associate) of sport science and educational psychology at Haute Ecole Pédagogique Lausanne
- Aurelie Zwang (University of Montpellier) Professor in Education and museology relating to the environment and ecological sciences: Works in the area of educational frameworks and conditions of encounters between subject and environment aswell as collaborative research in environmental education between actors and researchers (methods, modeling, postures)

Describe scope of scientific publications and reports on OP stemming from country in question (please include references of seminal publications and publications that relate to gender, race, class, or climate).

Attalie, M., Saint-Martin, J.(2017). Outdoor physical education in French schools during the twentieth century. *Journal of Adventure Education and Outdoor Learning*. 3 avril 2017. Vol. 17, n° 2pp. 148-160. DOI 10.1080/14729679.2016.1242082.

Cante, G. (2023). "Free Outdoor Play: Challenges and Perspectives for Environmental Education in France." Revue française d'éducation.

Point, M., & Dabaja, Z. (2022). "Nature-Based Play in France: Definitions and Impacts." Journal of Environmental Education.

National Report by O.N.E.F. (2021) on the development of natural play areas in urban parks in France.

Schnitzler, C., & Saint Martin, J. (2021). Éduquer aux Activités de Pleine Nature en France: un défi pour l'EPS du XXIe siècle?. *eJRIEPS. Ejournal de la recherche sur l'intervention en éducation physique et sport*, (49).

Schnitzler, C., Engstu, H., & Wassner, M. (2020). Unravelling threshold concepts in outdoor education. In *Threshold Concepts in Physical Education* (pp. 98-111). Routledge.

Urlacher-Schaal, A., Vors, O., Bouyat, M., Cante, G., & Schnitzler, C. (2023). Developing ethics and pro-environmental behaviors in PE: A mixed-methods research on an 8-month intervention study. *Staps*, *141*(3), 35-54.

PRACTICE

Name and describe organizations (e.g., Outdoor Play Canada) that support and promote OP.

Le FRENE, France (National Network for Environmental Education) formerly known as Réseau École et Nature (R.E.N.) was started in 1983 and provides environmental education to all ages Description: A network of regional associations promoting environmental education through outdoor activities. FRENE France supports local initiatives to integrate nature-based play into educational programs.

Les Petits Débrouillards (The Little Scientists)

- **Description**: A national popular education association that offers outdoor scientific workshops. They use play and experimentation to spark curiosity in children through outdoor activities.<br>**La Ligue de l'Enseignement (The Teaching League)**<br>- **Description**: An organization supporting environmental education and outdoor activities in recreational centers and schools. The League encourages the use of outdoor spaces for learning and play.<br>**Fédération des Parcs Naturels Régionaux de France (Federation of Regional Natural Parks)**<br>- **Description**: These parks offer educational programs and activities to encourage outdoor play and nature exploration. Initiatives include educational outings and community events centered on outdoor play.<br>-<br>**Found**<br>**Association Classe dehors (Outdoor School Association)**<br>- **Description**: An association dedicated to promoting outdoor school practices. It provides training for teachers and offers pedagogical resources to integrate outdoor play into school curricula.<br>- **Link**: https://classe-dehors.org/<br>- education.<br>**Fondation Nature & Découvertes**<br>**Description**: The foundation provides financial support for projects that promote children's access to nature, including initiatives focused on outdoor play and environmental education.<br><br>Fondation Terra Symbiosis<br>- Description: The foundation provides financial support for projects that promote children's access to nature, including initiatives focused on outdoor play

Name and describe networks (e.g., PLaTO-Net) that support and promote OP.

**Classe Dehors (Outdoor School Network)**<br>**Description**: A network of practitioners and educators promoting outdoor learning. The network organizes meetings, trainings, and shares best practices for utilizing natural spaces for play and learning. Is supported in part by the French Ministry of national education<br>classe-dehors.org : Bienvenue<br><br>Label Vie (Early childhood association for sustainable development practices): A network of early childcare professionals dedicated to bringing about awareness on ecological practices within daycare structures with training and conferences.<br>L'association - Label Vie<br><br>**Réseau Pédagogies par la Nature (RPPN) A network of forest schools and outdoor pre-schools**<br>https://www.reseau-pedagogie-nature.org/<br><br>Tous Dehors France (network of educational, early childhood and environmental professionals) promotes policies for outdoor contact with nature, supports other networks to share best practices<br>**https://www.tousdehors.fr/?NosActions**

POLICY

Name and describe national policy documents that support OP.

No significant policy found. The policies are very indirectly approaching the topic.

Quality charter In early childhood specifying the necessity for young children to be in contact with nature Principal N° 6 – "Direct contact with nature is essential for my development" (Arrêté 23 septembre, 2021)

HCFEA report on outspaces for children "Quelle nature pour les enfants dans les espaces public et la nature ? » 17 octobre 2024.

National Strategy for Protected Areas (2021-2030):

"The strategy aims to enhance young people's access to protected natural areas by integrating specific educational programs and facilitating outdoor activities for children."
Reference: Ministry of Ecological Transition (2021).
National Action Plan for Sport and Sustainable Development (PNASDD):

"The plan encourages the development of local initiatives to promote outdoor sports activities for children, with a strong focus on sustainability and environmental responsibility."
Reference: Ministry of Sports (2021).
National Strategy for Childhood and Adolescence (2020-2025):

"It is crucial to promote children's access to outdoor spaces and activities within an educational framework to enhance their physical and mental well-being."
Reference: Ministry of National Education and Youth (2020).

Name and describe fundings bodies that support OP.

Ministry of National Education and Youth
The ministry provides grants and support for educational projects that integrate outdoor learning and nature-based activities. Funding is often allocated through programs focused on environmental education and innovative teaching practices, including the *"École dehors"* (outdoor school) movement.

High council for Families, Children and Seniors (Haut Conseil de la famille, de l'enfance et de l'âge - HCFEA)
Hold public debates and provide public authorities with prospective and transversal expertise on issues linked to family and childhood, advancing age, the adaptation of society to aging and well-treatment, in a intergenerational approach.

2. Agence de la Transition Écologique (ADEME)
ADEME is the French Agency for Ecological Transition, offering financial support for projects that promote sustainable practices, including the development of natural play areas and green spaces in urban environments. They focus on projects that encourage children's access to nature and outdoor activities as part of broader ecological goals.

3. Fondations
Nature & Découvertes
The Nature & Découvertes Foundation provides grants for projects that reconnect children with nature. They support initiatives focused on outdoor play, environmental education, and the development of

natural play areas, often funding innovative projects that bring children closer to the natural environment.<br>Fondation Terra Symbiosis – provides grants to educational organizations and educational professionals who run programs allowing children to discover nature though play and learning experiences.

OTHER

Country: Greece

Name of assessor: Dr. Konstantina Rentzou, Assistant Professor, Department of Early Years Learning and Care, University of Ioannina, krentzou@uoi.gr

Date of assessment: October 14, 2024

Data form for outdoor play research, practice, and policy in Europe

This data form on outdoor play (OP) is to be completed in reference to the past 10 years and the country whom the assessor is representing.

OP is defined as a form of play that takes place outdoors [1]. Play is defined as voluntary engagement in an activity that is fun and/or rewarding and usually driven by intrinsic motivation. Please observe that this can refer to both children and adults. This document should not exceed two pages in total.

TERMINOLOGY

The term OP might not be directly or perfectly translated to the language/languages spoken in the country in question. Please name and describe terms that are used in country in question that could be conceptualised as or include outdoor play. Please also comment on how physical activity relates to these terms.

1. **Υπαίθριο παιχνίδι (outdoor play) =** Outdoor play (υπαίθριο παιχνίδι) is a common term used in Greece in order to describe play that takes place outdoors, in open air places. Usually when we refer to outdoor play (υπαίθριο παιχνίδι) we refer to a play that involves lots of physical activity and exploration.
2. **Παιχνίδι στην αυλή (play in the yard)=** it refers to outdoor play. Usually the outdoor space of a school is call yard (αυλή) and the term refers to children playing either in the yard of the school or in the yard of their home. It has equivalent connotation with the term υπαίθριο παιχνίδι and it involves physical activity and exploration.
3. **Ελεύθερο παιχνίδι σε εξωτερικούς χώρους (free play in outdoor spaces).** The term also refers to outdoor play. The emphasis is given to the free nature of play. Yet, the previous two terms also involve free play, although in some instances play can be organized by the educators. Free play in outdoor spaces involves lots of physical activity and exploration.
4. **Παιχνίδι σε υπαίθρια περιβάλλοντα (play in outdoor environments)**: This term is a combination of terms 1 and 2 and it refers to outdoor play in outdoor environments. It is usually used by educators to refer to play in outdoor learning environments.
5. **Παιχνίδι στη γειτονιά/στο δρόμο (play in the neighborhood / at the street): During** the previous decades, limited playgrounds were available. Children used to play freely in the neighborhood / at the street. This term refer to free play outdoors. It involved imagination (children used to use natural materials for their play), exploration and physical activity.

RESEARCH

Name active OP researchers and describe their research focus.

[1] Lee, Eun-Young, Louise de Lannoy, Lucy Li, Maria Isabel Amando de Barros, Peter Bentsen, Mariana Brussoni, Lindsay Crompton, et al. "Play, Learn, and Teach Outdoors—Network (PLaTO-Net): Terminology, Taxonomy, and Ontology." *International Journal of Behavioral Nutrition and Physical Activity* 19, no. 1 (June 15, 2022): 66. https://doi.org/10.1186/s12966-022-01294-0.

1. Dr. Gessiou, Georgia (https://www.researchgate.net/profile/Georgia-Gessiou-2) Her research focuses on the design and enhancement of outdoor learning and play (OPL) environments in Greece, with a strong emphasis on participatory design approaches. She has worked extensively to transform early years' outdoor spaces into resource-conscious environments that foster children's autonomy, cognitive development, and social-emotional well-being. Through comparative studies involving Greece, England, Türkiye and Norway, Gessiou highlights the diverse cultural contexts influencing outdoor play practices, advocating for its integration into the national curriculum. Her empirical work with Greek kindergarten teachers explores their strategies for promoting unstructured outdoor play, offering insights into how reflective pedagogical practices can enhance the educational potential of outdoor spaces. Her participatory action research has led to the co-design of outdoor environments with school communities, making them more inclusive and responsive to children's developmental needs. Gessiou's work calls for a re-evaluation of the undervaluation of outdoor play in Greek education, demonstrating its critical role in fostering creativity, resilience, and hands-on learning. Her contributions continue to shape the discourse around outdoor learning and inspire educators to integrate outdoor environments into early childhood education.<br>2. Professor Kouthouris Charilaos (https://www.researchgate.net/profile/Charilaos-Kouthouris). Professor Kouthouris' work focuses on Service Quality, Consumer Behavior, Sports Marketing, Recreation, Leisure Studies, Outdoor Activities, Outdoor Education, Sport Tourism and Leisure Management. He has published some research (https://scholar.google.com/citations?user=oIotk24AAAAJ&hl=el) on teaching outdoor adventure activities in preschools, on the effects of "Greening schoolyards", on environmental awareness, learning, physical, social and mental health of primary school students, on correlations between participation of children in outdoor activities and the effects on environmental education and environmental behavior and on how participation in outdoor learning education and participation in outdoor recreational sports activity intervention programs affects children's attitudes towards group work.<br>3. Dr. Zafeiroudi Aglaia (https://www.researchgate.net/profile/Aglaia-Zafeiroudi) Dr. Zafeiroudi's research focuses on recreational activities, dance and outdoor education. She has published some research (https://scholar.google.com/scholar?start=20&q=zafeiroudi+a&hl=el&as_sdt=0,5) on outdoor recreation activities of children across ages and its relation with environmental education and environmental responsible behavior. Of interest to the project might be the literature review of outdoor play practice in Greece (https://macrojournal.org/index.php/jse/article/view/252/234)

Describe scope of scientific publications and reports on OP stemming from country in question (please include references of seminal publications and publications that relate to gender, race, class, or climate).

A number of studies (mainly Master dissertations and PhD theses) that have been conducted in Greece aim to explore the association between outdoor play and outdoor activities and environmental education / children's environmental education (see work mentioned in the previous section).<br>In addition, there is another strand of studies (again mainly Master dissertations and PhD theses) that aim to explore children's play in the outdoor environment, mainly using an architectural / design lens, as they explore the effects of the organization and design of playgrounds and other urban spaces in children's play (e.g.<br>1. Botsoglou, K. (2010). Outdoor play spaces and the child: Quality, Security, Pedagogical applications" Athens: Gutenberg (In Greek)<br>2. Germanos, D. (2015). «The Place as factor of the pedagogical quality of space». In Germanos, D. Liapi, M. (eds), Places for Learning Experiences. Think, Make, Change. Digital Proceedings of the Symposium with International Participation, Thessaloniki, 09-10 January 2015. Athens: Greek National Documentation Centre, 46-55, http://epublishing.ekt.gr/el/12239.

3. Germanos, D. Pedagogical transformation of the school space: An approach to upgrading the educational environment through changing the child's relationship with the space. In Αρχιτεκτονικές & εκπαιδευτικές παρεμβάσεις για τη δημιουργία τόπων μάθησης στο Νηπιαγωγείο [Architectural & educational interventions to create learning places in kindergarten]; Gourgiotou, E., Ouggrinis, K., Eds.; Δισίγμα: Thessaloniki, Greece, 2015, pp 45-58 (In Greek)

Finally, another strand of research emphasizes children's autonomy in outdoor environments and in the forest (e.g. https://orcid.org/0000-0002-4610-8418) as well as risky play (e.g. https://doi.org/10.12681/dial.10512) and the reasons why children in Greece are not outdoors (https://paizontas.gr/wp-content/uploads/2021/02/01_article.pdf)

PRACTICE

Name and describe organizations (e.g., Outdoor Play Canada) that support and promote OP.

1. **Paizontas.gr (https://paizontas.gr/)** PLAYING is a non profit organization for the promotion of play in childhood and the advocacy of children's right to play, supporting their motor, cognitive, emotional, social development and their physical and mental health. Their vision is to contribute to the health of all children through free and non guided play. PLAYING designs, implements and contributes to programs that promote child play; they offer training and talks to pre-school teachers, teachers and parents about for the value of play; they provide consultation regarding the indoor and outdoor design of play areas alongside with personnel training.

Name and describe networks (e.g., PLaTO-Net) that support and promote OP.

I am not aware of any networks that support and promote OP

POLICY

Name and describe national policy documents that support OP.

To the best of my knowledge there are not national policy documents that support OP. There are some references to OP in the national curricula especially those addressed to lower educational level.
In addition, the new framework for preschool education of children 0-4 in Greece, has been developed under the Project Kipseli (https://www.anavathmisi.gr/n-4837-2021-%CF%80%CF%81%CF%8C%CE%B3%CF%81%CE%B1%CE%BC%CE%BC%CE%B1-%CE%BA%CF%85%CF%88%CE%AD%CE%BB%CE%B7-%CE%BA%CE%B1%CE%B9-%CF%83%CE%B7%CE%BC%CE%B1%CE%BD%CF%84%CE%B9%CE%BA%CE%AD%CF%82-%CE%B4/) that has been developed with the aim to improve the quality of ECEC offered in child care and infant/childcare center, stresses the importance of the OP for preschool-aged children. Specifically, in this framework, that is currently piloted in some regions of Greece, OP is diversified from play in general and from playing indoors and is referred to as a main component of the proposed pedagogical program. In the chapter that is related to OP, there is reference to the significance of OP, to the current status of OP in Greece (extensive reference in made to the limited time children spent outdoors and the lack of outdoor spaces) and suggestion are formulated for early childhood educators in order to offer more opportunities for OP to children. As supporting materials for parents and educators are suggested the 2 manuals and the videos that have been developed by the organization PAIZONTAS – PLAYING (https://paizontas.gr/?page_id=3108 AND https://paizontas.gr/?page_id=3149

Name and describe fundings bodies that support OP.

I am not aware of any funding bodies at the national/local level that support OP

OTHER

At the university of Thesally, from the Laboratory of Applied Leisure Science: Nature, Sports, Arts and Educational Program is offered with the title "Outdoor Education The outdoor Agogue (education) in education – life skills, environment, physical recreation" (https://learning.uth.gr/outdoor-education_h-%ce%b1%ce%b3%cf%89%ce%b3%ce%ae-%cf%85%cf%80%ce%b1%ce%af%ce%b8%cf%81%ce%bf%cf%85-%cf%83%cf%84%ce%b7%ce%bd-%ce%b5%ce%ba%cf%80%ce%b1%ce%af%ce%b4%ce%b5%cf%85%cf%83%ce%b7-%ce%b4%ce%b5/) The program is addressed to educators working in all educational levels. This training aims to give the opportunity to all participating students to become competent to plan from a simple to a complex Outdoor Education program and to attempt to adequately and safely guide groups of students or other individuals in educational activities in nature , in recreational parks, in suitably designed urban areas free of construction.

Country: Hungary

Name of assessor: Tamas

Date of assessment: 15th Nov. 2024

Data form for outdoor play research, practice, and policy in Europe

This data form on outdoor play (OP) is to be completed in reference to the past 10 years and the country whom the assessor is representing.

OP is defined as a form of play that takes place outdoors [1]. Play is defined as voluntary engagement in an activity that is fun and/or rewarding and usually driven by intrinsic motivation. Please observe that this can refer to both children and adults. This document should not exceed two pages in total.

TERMINOLOGY

The term OP might not be directly or perfectly translated to the language/languages spoken in the country in question. Please name and describe terms that are used in country in question that could be conceptualised as or include outdoor play. Please also comment on how physical activity relates to these terms.

The literal translation of outdoor play is “szabadtéri / kültéri játék” in Hungarian which means any type of play outdoors (included: sport related activities; and backyard games) played by the children with or without adult supervision. Hungary has a long tradition of playing outside in early childhood education, even primary schools offer opportunities for students almost every day. Play and movement must be part of the early childhood education in every day. These activities could be adult-initiated or children-initiated activities.<br><br>For the families, outdoor play with the young children is an important everyday activity in municipality playgrounds. In the last 15 years many well constructed outdoor playing area have been built inside kindergartens and in municipality parks.

RESEARCH

Name active OP researchers and describe their research focus.

Tamás Csányi (early childhood physical education, motor learning, and development), but I am not really an “outdoor play researcher”. Based on my scientific research network in early childhood and school education, I do not know a Hungarian researcher, who is investigating specifically this topic.

Describe scope of scientific publications and reports on OP stemming from the country in question (please include references of seminal publications and publications that relate to gender, race, class, or climate).

Unfortunately, very few empirical scientific data are available from our country. Early childhood and school national curriculum policies highlight the importance of outdoor play (e.g. national kindergarten program policy; Hungarian National Core Curriculum). Almost every pedagogical textbook includes the different forms of play, including outdoor play in the field of early childhood education.

[1] Lee, Eun-Young, Louise de Lannoy, Lucy Li, Maria Isabel Amando de Barros, Peter Bentsen, Mariana Brussoni, Lindsay Crompton, et al. “Play, Learn, and Teach Outdoors—Network (PLaTO-Net): Terminology, Taxonomy, and Ontology.” *International Journal of Behavioral Nutrition and Physical Activity* 19, no. 1 (June 15, 2022): 66. https://doi.org/10.1186/s12966-022-01294-0.

Effect of environmental changes on the physical activity of preschool children measured with an accelerometer during free play time. HUNGARIAN SPORT SCIENCE REVIEW 13 : 2 (50) p. 28-28. , 1 p. (2012)

PRACTICE

Name and describe organizations (e.g., Outdoor Play Canada) that support and promote OP.

Green Kindergartens Network (Hungary) : https://zoldovoda.hu/
BirdLife Hungary: https://mme.hu/en/az-mme-az-alapszabaly-szerint
Ökoschools Hungary:
https://www.oktatas.hu/kozneveles/pedagogiai_szakmai_szolgaltatasok/fenntarthatosagra_neveles/okoiskolak_Magyarorszagon

Name and describe networks (e.g., PLaTO-Net) that support and promote OP.

POLICY

Name and describe national policy documents that support OP.

Early childhood and school national curriculum policies highlight the importance of outdoor play (e.g. national kindergarten program policy; Hungarian National Core Curriculum). Almost every pedagogical textbook includes the different forms of play, including outdoor play in the field of early childhood education.

Name and describe fundings bodies that support OP.

-

OTHER

Country: **Ireland**

Name of assessor: Thomas Morgenthaler & Michelle Bergin

Date of assessment: **11/11/2024**

Data form for outdoor play research, practice, and policy in Europe

Research questions:

- **What is the state of outdoor play practice, research, and policy in European countries and in Europe as a whole?**
- **What opportunities exist for furthering outdoor play practice, research, and policy in the European region?**

This data form on outdoor play (OP) is to be completed in reference to the past 10 years and the country whom the assessor is representing.

OP is defined as a form of play that takes place outdoors [1]*. Play is defined as voluntary engagement in an activity that is fun and/or rewarding and usually driven by intrinsic motivation. Please observe that this can refer to both children and adults. This document should not exceed two pages in total.*

TERMINOLOGY

The term OP might not be directly or perfectly translated to the language/languages spoken in the country in question. Please name and describe terms that are used in country in question that could be conceptualised as or include outdoor play. Please also comment on how physical activity relates to these terms.

Ireland adopts similar terms to those in Canada with active play and outdoor play commonly used. Risky play is also used, although is not used as frequently in Irish Policy, Research or Practice.<br><br>Outdoor play (or active play) is seen as a type of physical activity distinct of sport, active transport, PE etc. For adults, the term recreational physical activity would be more common, however, it is often difficult to differentiate leisure or recreational physical activity to sport.

RESEARCH

Name active OP researchers and describe their research focus.

University College Cork<br><br>1. Dr. Helen Lynch (occupational science and occupational therapy as it relates to children's rights to play, inclusive outdoor play, Universal Design of playgrounds, participatory play space development and design, and how professions like occupational therapist understand and use play in practice)<br><br>2. Thomas Morgenthaler is a PhD student occupational science and occupational therapy as it relates to children's rights to play, inclusive outdoor play, environmental characteristic that enable and enhance outdoor play, evaluation of playground’s play value. Thomas is also affiliated with Queen Margret University Edinburg Scotland.

[1] Lee, Eun-Young, Louise de Lannoy, Lucy Li, Maria Isabel Amando de Barros, Peter Bentsen, Mariana Brussoni, Lindsay Crompton, et al. “Play, Learn, and Teach Outdoors—Network (PLaTO-Net): Terminology, Taxonomy, and Ontology.” *International Journal of Behavioral Nutrition and Physical Activity* 19, no. 1 (June 15, 2022): 66. https://doi.org/10.1186/s12966-022-01294-0.

3. Rianne Janson is a PhD student in Occupational Science with a research focus on participatory play space development and design. Rianne is also affiliated with Luleå University of Technology in Sweden
4. Dr Michelle Bergin research is about outdoor play in school yards and play of Irish traveller. Michelle is currently a post doc at UCC.
5. Dr Ines Wenger completed her PhD 2023 on the topic of inclusive play spaces from the perspectives of children and playground provider. Ines is currently affiliated with a university in Switzerland but was affiliated with UCC and Luleå University of Technology in Sweden during her PhD studies.

The Crann centre (Rehabilitation clinic, Cork)

6. Dr Alice Moore completed her PhD 2023 at UCC on the topic of Universal Design of public play spaces. Dr. Moore is currently research lead at rehabilitation clinic in cork

Mary Immaculate College University of Limerick

7. Dr. Suzanne Egan, background is psychology, the impact of various activities on early development, such as reading, screen-time and outdoor play

8. Dr Jennifer Pope (environmental influence on outdoor play)

Dublin City University

9. Dr Carol Barron's research interests include children's play and games, physical activity play and childhood obesity and participatory research methodologies with children., local housing and spaces where children play
10. Christina O'Keeffe: PhD student, children's understanding of play, autisitc play, teachers use of play

Technological University Dublin

11. Fiona Armstrong (understanding the benefits of engaging in unstructured play incorporating risk for the developing child)

Hibernia College

12. Dr *Margaret Kernan* (outdoor play in early childhood centers, History of playground in Irealnd, Affordances)

There are also some research unites which contribute to outdoor play and physical activity research:

1. Children's Research Network of Ireland have a special interest group that focuses on Outdoor play, Leisure and Activity. Focus is on research and practice
2. Irish Physical Activity Research Collaboration-i-parc.ie
3. Physical Education, Physical Activity and Youth Sport (PEPAYS) Ireland Research Centre-https://www.ul.ie/ehs/pess/pepays-ireland/about
4. *Adventurous Play & Outdoor Learning Project – DCU STEM Department Involved-*
5. *UNESCO Chair in Inclusive PE Sport Recreation and Fitness, Munster Technological Un, Tralee, Co Kerry, Ireland – Also linked with Health & Leisures Studies Department*
6. *Annual 'Revolutionising Education Through Play' Conference at MIC Limerick : https://www.mic.ul.ie/news/2024/mic-to-shine-a-light-on-the-power-of-play-with-major-international-education-conference*
7. P4Play research group was an European fundet project with 3 aditional concortium paprtners across Europe which focused on outdoor play.

Describe scope of scientific publications and reports on OP stemming from country in question (please include references of seminal publications and publications that relate to gender, race, class, or climate).

Currently no national survey provides evidence of how much time Irish children are spending in outdoor play per day but there are other national research projects that give some understanding of outdoor play in Ireland:

1. Growing up in Irland https://www.growingup.gov.ie/about-growing-up-in-ireland/ : Publications include data on physical activity; children's top three pastimes- including play outside and play with friends; parents' perspectives of safety and play outside. Example Reports & Fact Sheets
   https://www.growingup.gov.ie/pubs/BKMNEXT154.pdf
   https://www.growingup.gov.ie/pubs/SUSTAT11.pdf
   https://www.growingup.gov.ie/pubs/E3-Presentation-Egan-Pope.pdf
   Studies drawing on GUI Data relevant to physical activity
   https://www.growingup.gov.ie/information-for-researchers/all-publications-using-growing-up-in-ireland-data/page/3/?orderby=url&order=asc
   https://www.artscouncil.ie/uploadedFiles/Arts-and-cultural-participtiion-GUI.pdf
2. The Irish Longitudinal Study on Ageing (TILDA) is a large-scale, nationally representative, longitudinal study on ageing in Ireland, so this study does not focu on children but it has publication on outdoor activity of other age groups: https://www.tcd.ie/search/q/?tcdsearchq=leisure&tcdsearchcollection=site&tcdsearchsitetitle=The+Irish+Longitudinal+Study+on+Ageing+%28TILDA%29&tcdsearchsitesearch=http%3A%2F%2Ftilda.tcd.ie

Physical activity focus – focus on all of Ireland, report on physical activity

1. Carlin, A., Connolly, S., Redpath, T., Belton, S., Coppinger, T., Cunningham, C., Donnelly, A., Dowd, K., Harrington, D., Murtagh, E., Ng, K., O'Brien, W., Rodriguez, L., Woods, C., McAvoy, H., & Murphy, M. (2024). Results from Ireland North and South's 2022 report card on physical activity for children and adolescents. *Journal of Exercise Science & Fitness*, *22*(1), 66–72.https://doi.org/10.1016/j.jesf.2023.12.003
2. Woods, C., Ng, K., Britton, U., McClelland, J. F., O'Keeffe, B., Sheikhi, A., McFlynn, P., Murphy, M., Goss, H., Behan, S., Philpott, C., Lester, D., Adamakis, M., Costa, J., Coppinger, T., Connolly, S., Belton, S., & O'Brien, W. (2023). *Children's Sport Participation and Physical Activity Study 2022*. Physical Activity for Health Research Centre, Department of Physical Education and Sport Sciences, University of Limerick, Limerick, Ireland, Sport Ireland Children's Sport and Healthy Ireland, Dublin, Ireland and Sport Northern Ireland, Belfast, Northern Ireland. https://doi.org/10.34961/RESEARCHREPOSITORY-UL.23609157
3. *Irish Health Behaviour in School-aged Children (HBSC) Study : https://www.universityofgalway.ie/hbsc/*

Publications which do not have a national reach are listed and clustered below

Educational context:

Research papers on the topic of school yard play

1. Bergin, M., Boyle, B., Lilja, M., & Prellwitz, M. (2024a). Exploring with children, play in Irish primary schoolyards. *International Journal of Play*, *13*(2), 157–172. https://doi.org/10.1080/21594937.2024.2355443
2. Bergin, M., Boyle, B., Lilja, M., & Prellwitz, M. (2024b). Irish Schoolyards: Teacher's Experiences

of Their Practices and Children's Play"It's Not as Straight Forward as We Think". *Journal of Occupational Therapy, Schools, & Early Intervention*, *17*(2), 259–278. https://doi.org/10.1080/19411243.2023.2192201
3. O'Keeffe, C., & McNally, S. (2024). 'It's one of your main things in life like': How children's conceptualisations of play can inform educational practice. *Education 3-13*, 1–14. https://doi.org/10.1080/03004279.2024.2405638
4. Kernan, M., & Devine, D. (2010). Being confined within? Constructions of the good childhood and outdoor play in early childhood education and care settings in Ireland. *Children & Society*, *24*(5), 371-385. **https://doi.org/10.1111/j.1099-0860.2009.00249.x**

Forest primary schools (not a primary focus on play, but play is part of findings):

1. Whelan, J., & Kelly, O. (2023). Experiential, relational, playful pedagogy in Irish primary schools – possibilities offered by Forest School. *Irish Educational Studies*, 1–25. https://doi.org/10.1080/03323315.2023.2280224
2. Whelan, J., & Kelly, O. (2024). Towards flourishing: The understanding and experience of the staff in one Irish primary school of Forest School. *Journal of Adventure Education and Outdoor Learning*, 1–19. https://doi.org/10.1080/14729679.2024.2404653

Outdoor Play & Learning

1. Abbott, K., & Flynn, S. (2024). Outdoor education, interaction and reflection: a study of Irish outdoor ECEC. *Irish Educational Studies*, *43*(3), 371-391.
2. O'Keeffe, C., & McNally, S. (2020). Perspectives of early childhood teachers in Ireland on the role of play during the pandemic. *PsyArXiv. August*, *6*.
3. Kernan, M., Casey, M., & Dowdall, M. (2023). Embracing Changes Outdoors for Children Under 3, Pilot Study of a Community of Research and Practice: Evaluation Report.

Local Neighborhoods

Neighborhood Environment influence on play including social and physical environment:

1. Barron, C. (2013). Physical activity play in local housing estates and child wellness in Ireland. *International Journal of Play*, *2*(3), 220–236. Scopus. https://doi.org/10.1080/21594937.2013.861262
2. Egan, S. M., & Pope, J. (2024). Streets ahead: Neighborhood safety and active outdoor play in early childhood using a nationally representative sample of 5-year-olds. *Child Development*, cdev.14132. https://doi.org/10.1111/cdev.14132
3. Pope, J., Egan, S. M., & Hilliard, E. (2021). The Great Outdoors: A Bioecological Systems Approach to Outdoor Play. In A. Leavy & M. Nohily (Eds.), *Perspectives on Childhood*. Cambridge Scholars Publishing.
4. Lynch, H., Moore, A., Jackson, J., Fitzsimons, J., Cotel, K., Carroll, M., Morgenthaler, T., Mula, A., Jansens, R., Loudoun, F. M., Wenger, I., Bergin, M., Viega Seijo, S., & Vinscon, S. (2021). *Cork freedome of the city summery of children and young peoples submissions: Drawings and written pieces Summer 2021*. University College Cork, Ireland. https://consult.corkcity.ie/ga/system/files/materials/5184/5871/CORK%20FREEDOM%20OF%20THE%20CITY%20SUMMARY%20OF%20CHILDREN%20AND%20YOUNG%20PEOPLES%20SUBMISSIONS_0.pdf

Play of children with disabilities

General:

1. Barron, C., Beckett, A., Coussens, M., Desoete, A., Cannon Jones, N., Lynch, H., Prellwitz, M.,

Fenney Salkeld, D., & De Gruyter Open. (2017). *Barriers to play for children and young persons*.

Children with autism outdoor play in schools and neighborhood

1. Blake, A., Sexton, J., Lynch, H., Moore, A., & Coughlan, M. (2018). An exploration of the outdoor play experiances of preschool children with autim spectrum disorder in an Irish preschool setting. *Today's Children Tomorrow's Parents: An Interdisciplinary Journal*, *47–48*, 101–116.
2. Coughlan, M., & Lynch, H. (2024). "Can I Play Too?" A Qualitative Study of Outdoor Play and Participation Among Autistic Preschoolers. *The American Journal of Occupational Therapy*, *78*(4), 7804185130. https://doi.org/10.5014/ajot.2024.050732
3. Fahy, S., Delicâte, N., & Lynch, H. (2021). Now, being, occupational: Outdoor play and children with autism. *Journal of Occupational Science*, *28*(1), 114–132. https://doi.org/10.1080/14427591.2020.1816207

Children and the Natural Environment and play:

1. Kilkelly, U., Lynch, H., Moore, M., O'Connell, A., & Field, S. (2016). *Children and the Outdoors: Contact with the outdoors and natural heritage among children aged 5 to 12: Current trends, benefits, barriers and research requirements*. Ireland: Heritage Council. http://rgdoi.net/10.13140/RG.2.1.3083.3523
2. Britton, E., Carlin, C., & Kindermann, G. (2020). *Connecting with Nature for Health and Wellbing*. NUI Galway & Environmental Protection Agency. https://www.epa.ie/publications/research/environment--health/JS---NEAR-Toolkit-FINAL-V1.6-1Oct20.pdf (Not particular about outdoor play but reference to outdoor play and play spaces to play in nature or with biodiversity)

Irish Traveller Children's play and outdoor play

1. Bergin, M., Boyle, B., Liljia, M., & Prellwitz, M. (2023). Irish Traveller Children's play: A scoping review. *Journal of Child and Family Studies*, *32*, 3860–3875. https://doi.org/10.1007/s10826-023-02695-w

Direct Provision & Migrant children

1. Dunbar, R., Burke, L., Candon, N., Reid, M., Crivits, S., Wrenn, S., & Shilova. (2020). *DIRECT PROVISION'S IMPACT ON CHILDREN: A HUMAN RIGHTS ANALYSIS- A Submission to the Minister for Children, Disability, Equality, Integration and Youth*. NUI Galway. https://www.universityofgalway.ie/media/irishcentreforhumanrights/files/reports/Direct-Provision-Report_-ICHR_Final-23.09.pdf(References to Play Rights provision)
2. Darmody, M., & Smyth, E. (2017). Out-of-School Social Activities among Immigrant-Origin Children Living in Ireland. *The Economic and Social Review*, *48*(4), 419–439.
3. Coughlan, B., Doherty, E., O'Neill, C., & McGuire, B. (2014). Minority Status, Social Welfare Status and their Association with Child Participation in Sporting, Cultural and Community Activities. *THE ECONOMIC AND SOCIAL REVIEW*, *45*(1), 65–85.

Risky play in the neighborhood:

1. Hinchion, S., McAuliffe, E., & Lynch, H. (2021). Fraught with frights or full of fun: Perspectives of risky play among six-to-eight-year olds. *European Early Childhood Education Research Journal*, *29*(5), 696–714. https://doi.org/10.1080/1350293X.2021.1968460

Patterns of play:

1. Lynch, H. (2009). Patterns of activity of Irish children aged five to eight years: City living in Ireland

today. *Journal of Occupational Science*, *16*(1), 44–49. https://doi.org/10.1080/14427591.2009.9686641

Play and well being:

1. Moore, A., & Lynch, H. (2018). Understanding a child's conceptualisation of well-being through an exploration of happiness: The centrality of play, people and place. *Journal of Occupational Science*, *25*(1), 124–141. https://doi.org/10.1080/14427591.2017.1377105

Playground and Play space research

Participatory practices in playground development and design:

1. Jansens, R., Prellwitz, M., Olofsson, A., & Lynch, H. (2023). The Representation of Children's Participation in Guidelines for Planning and Designing Public Playspaces: A Scoping Review with "Best Fit" Framework Synthesis. *International Journal of Environmental Research and Public Health*, *20*(10), 5823. https://doi.org/10.3390/ijerph20105823
2. Lynch, H., Jansens, R., & Prellwitz, M. (2025). Having a Say in Places to Play: Children with Disabilities, Voice and Participation. In K. Bishop & K. Dimoulias (Eds.), *The Routledge Handbook on the Influence of Built Environments on Diverse Childhoods*. Routledge. https://doi.org/10.4324/9781003284406

Inclusive Play Space Design, Universal Design

1. Lynch, H., Moore, A., Edwards, C., & Horgan, L. (2018). *Community Parks and Playgrounds Community Parks and Playgrounds: Intergenerational Participation through Universal Design Final Report For The Centre for Excellence in Universal Design at the National Disability Authority*. National Disability Authority. http://rgdoi.net/10.13140/RG.2.2.22422.60486
2. Lynch, H., Moore, A., Edwards, C., & Horgan, L. (2020). Advancing play participation for all: The challenge of addressing play diversity and inclusion in community parks and playgrounds. *British Journal of Occupational Therapy*, *83*(2), 107–117. https://doi.org/10.1177/0308022619881936
3. Moore, A., Boyle, B., & Lynch, H. (2023). Designing for inclusion in public playgrounds: A scoping review of definitions, and utilization of universal design. *Disability and Rehabilitation: Assistive Technology*, *21*(3), 422–441. https://doi.org/10.1080/17483107.2021.2022788
4. Moore, A., & Lynch, H. (2015). Accessibility and usability of playground environments for children under 12: A scoping review. *Scandinavian Journal of Occupational Therapy*, *22*(5), 331–344. https://doi.org/10.3109/11038128.2015.1049549
5. Moore, A., Lynch, H., & Boyle, B. (2020). Can universal design support outdoor play, social participation, and inclusion in public playgrounds? A scoping review. *Disability and Rehabilitation*, *44*(13), 3304–3325. https://doi.org/10.1080/09638288.2020.1858353
6. Moore, A., Lynch, H., & Boyle, B. (2022). A national study of playground professionals universal design implementation practices. *Landscape Research*, *47*(5), 611–627. https://doi.org/10.1080/01426397.2022.2058478

Environmental qualities of play spaces (socio-spatial, physical , social, atmothphere)

1. Morgenthaler, T., Lynch, H., Loebach, J., Pentland, D., & Schulze, C. (2024). Using the theory of affordance to understand environment–play transactions: Environmental taxonomy of outdoor play space features—A scoping review. *American Journal of Occupational Therapy*, *78*(4), 7804185120. https://doi.org/10.5014/ajot.2024.050606
2. Morgenthaler, T., Schulze, C., Pentland, D., & Lynch, H. (2023). Environmental qualities that enhance outdoor play in community playgrounds from the perspective of children with and without disabilities: A scoping review. *International Journal of Environmental Research and Public Health*, *20*(3), 1763. https://doi.org/10.3390/ijerph20031763

3. Wenger, I., Kantartzis, S., Lynch, H., Schulze, C., & Jackson, J. (2024). Making secret hiding places: An occupation of childhood. *Journal of Occupational Science*, *31*(1), 118–131. https://doi.org/10.1080/14427591.2023.2240815
4. Wenger, I., Lynch, H., Prellwitz, M., & Schulze, C. (2024). Children's experiences of playground characteristics that contribute to play value and inclusion: Insights from a meta-ethnography. *Journal of Occupational Science*, *31*(3), 405–432. https://doi.org/10.1080/14427591.2023.2248135

Play Policy research papers or book chapters:

1. Lynch, H., Moore, A., & Prellwitz, M. (2018). From Policy to Play Provision: Universal Design and the Challenges of Inclusive Play. *Children, Youth and Environments*, *28*(2), 12. https://doi.org/10.7721/chilyoutenvi.28.2.0012
2. Armstrong, F., & Gaul, D. (2023). Review of the international play policies and their contribution to supporting a child's right to play. *Children & Society*, *37*(6), 2179–2195. https://doi.org/10.1111/chso.12773
3. Barron, C. (2022). "Are they going to do anything with this info"? Implementing children's perspectives on play and recreation policies. In D. Horgan & D. Kennan (Eds.), *Child and Youth Participation in Policy, Practice and Research* (pp. 57–70). Routledge. https://doi.org/10.4324/9781003099529

Historical development of playground

1. Kernan, M. (2005). Developing Citizenship Through Supervised Play: The Civics Institute of Ireland Playgrounds, 1933-75. History of Education, 34(6), 675–687. https://doi.org/10.1080/00467600500313955

COVID 19 and Outdoor play:

Of children with disabilities:

1. Bulgarelli, D., Bianquin, N., Barron, C., & Emmett, M.-J. (2023). Outdoor play of children with and without disabilities. Insights from the Covid-19 pandemic in Ireland and Italy. *European Journal of Special Needs Education*, *38*(3), 334–348. https://doi.org/10.1080/08856257.2022.2089508

Professional attitudes towards play (not only outdoor play), use of play in practice:

1. Lynch, H., Prellwitz, M., Schulze, C., & Moore, A. H. (2017). The state of play in children's occupational therapy: A comparison between Ireland, Sweden and Switzerland. *British Journal of Occupational Therapy*, *81*(1), 42–50. https://doi.org/10.1177/0308022617733256
2. Brown, T., & Lynch, H. (2023). Children's play–work occupation continuum: Play-based occupational therapy, play therapy and playwork. *Canadian Journal of Occupational Therapy*, *90*(3), 249–256.

PRACTICE

Name and describe organizations (e.g., Outdoor Play Canada) that support and promote OP.

Organisations with National Remit:

8. The Government of Ireland has a number of departments that aim to promote active and outdoor play, including The Department of Children, Equality, Disability, Integration and Youth, and The Department of Health.
9. Department of Rural and Community Development - Outdoor Recreation Unit – Outdoor Recreation Strategy & Infrastructure Scheme https://www.gov.ie/en/organisation/department-of-rural-and-community-development/
10. Department of Health – Healthy Ireland: https://www.getirelandactive.ie/ including The Get Ireland Active National Database - interactive activity, sport and recreation hub.
11. Department of Children & Youth Affairs- https://www.gov.ie/en/campaigns/lets-play-ireland/
N.B: Department of Children and Youth Affairs.
(DCYA). Participation, Play and Recreation and Culture Unit. As part of Young Ireland Policy Strategy Implementation
12. Better Start Ireland is a national initiative established by the Department of Children, Equality, Disability, Integration and Youth. Better Start promotes quality and inclusion in Early Learning and Care (ELC) settings for children from birth to six years of age in Ireland.
13. Early Childhood Ireland is the leading membership organisation in the early years sector, focused on our compelling vision of putting the child at the centre of our work.
14. *Lets play Ireland. National play day and resources but more funding body*
15. *A playful city. not-for-profit that works to create more playful, healthy and inclusive places by providing end-to-end consultation, placemaking and design services.*
16. *Barnardos: have a training in outdoor play*
17. An Ireland Branch of the International Play association is currently forming which should cater to all of Ireland

Organisations with Local Remit:

In addition to the national organisations listed above, there are also local organisations from the public, private and charity sectors who are promoting outdoor play. These organisations are integral to delivering the policies and agendas set out by the Government. Examples.

18. *Cork Child Friendly City initiative*
19. *Let's play cork: developed a series of play resources and equipment which are free to use and are available, organises events with play focus in the community and free, works with the city council and other praxis partner*

Local Children & Young Persons Services Committees: Examples of Supports via workshops and conferences

1. **2021 Outdoor Play Conference**https://www.cypsc.ie/active-and-healthy-0-6-years-subgroup/outdoor-play.3324.html

- Playground Design Workshop: https://monaghan.ie/monaghan-county-council-playground-design-workshop/

- Childcare Committee: Outdoor Play Workshops: Example: https://www.kkccc.ie/event/outdoor-play-learning-workshop/

Name and describe networks (e.g., PLaTO-Net) that support and promote OP.

No national networks exist solely focusing on outdoor play but several initiatives support play and outdoor play:

- CYPSC's Get Ireland Active
- Let's Play Ireland Government Initiatives
- Irelands Branch of the International Play association which is currently forming

POLICY

Name and describe national policy documents that support OP.

1. Healthy Ireland Policy https://www.gov.ie/en/policy-information/706608-healthy-ireland-policies/ incorporates: National Physical Activity Plan (2016)- Implementation review in 2022 & National Obesity Policy & Action Plan (2016-2025) (physical activity identified as important factor)
2. First 5, Strategy for Babies, Young Children and their Families, 2019-202846- resources associated with the Active Play Everyday initiative
3. National Creative Youth Plan : https://www.creativeireland.gov.ie/en/blog/creative-youth-plan-2023-2027/
4. Health Services Ireland and Department for Health. (2024). Every Move Counts – National Physical Activity and Sedentary Behaviour Guidelines for Ireland. https://about.hse.ie/news/every-move-counts-new-guidelines-aim-to-encourage-people-of-ireland-to-sit-less-and-get-more-active/. Play is references as a way of increasing physical activity levels.
5. Government of Ireland. (2024). Policy Framework for Children and Young People 2023 2028. https://assets.gov.ie/232106/7d9ef26f-aeed-49f3-af95-f6fbc1fa6b61.pdf. This framework strives to place children and young people at the centre of policy, while also addressing the most pressing issues for children and young people, both now and over the lifetime of the framework. N.B. Play & Recreation Highlighted as key are with specific Actions
6. Government of Ireland. (2023).Embracing Ireland's Outdoors: National Outdoor Recreation Strategy 2023-2027 https://www.gov.ie/en/policy-information/43eee-embracing-irelands-outdoors-national-outdoor-recreation-strategy-2023-2027/. This aims to facilitate the

sustainable development and management of outdoor recreation and increased participation for health and economic benefits.

7. Tusla's Early Years Inspectorate. (2023). When the Roof is the Sky: Guidance for the registration and inspection of early years services operating outdoors. https://www.tusla.ie/news/new-outdoor-play-guidance-for-early-years-services/. This guidance provides early years services with additional guidance when operating or proposing to register a childcare service which operates outdoors.

National play polices:

8. Department of Health and Children. (2004). *Ready, Steady, Play! A National Play Policy*. Dublin: The Stationery Office. https://assets.gov.ie/24440/03bb09b94dec4bf4b6b43d617ff8cb58.pdf. Ireland's Play Strategy aims to ensure all children have access to a minimum standard of play and recreation facilities.
9. Office of the Minister for Children. (2007). *Teenspace: National Recreation Policy for Young People'*. The Stationery Office. https://assets.gov.ie/24432/f417e788ba97413f925c74d2c4b5b275.pdf Youth focus and recreation focus for youth
10. Irisch report from a policy and practice standpoint:

https://www.gov.ie/en/publication/28704-play-and-recreation-reviews-and-good-practice-examples/

A recent National Review of Play and Recreation Policies in Ireland in 2024 was commissioned by the Youth Reform, Strategy and Participation Unit of the Department of Children, Equality, Disability, Integration and Youth (Barron, C., Buckley, N. 2024). The review defines outdoor play as: "Play options executed, opportunities created, or actions taken by children when their curiosity and sense of wonderment are triggered in their natural outdoor world." (Dietze and Kashin, 2018)
This review identified that:

- **The responsibility for play provision is often shared between different departments in city and county councils.**
- **Outdoor play is addressed within all 31 Local Authority Development Plans (LADPs) and 26 play and or policies have been developed across the 31 city and county councils with a strong emphasis on playgrounds, consultation processes, community relationships and the child population of the various councils. Play awareness, accessibility and inclusivity, play infrastructure, collaboration, play for health, play design, natural spaces, governance and operations, staff training and development, and safety were common dimensions of play policies.**
- **An increase of 6.2 % in playground provision in Ireland was identified over the last 6 years with 960 playgrounds and 291 MUGA's & skateparks in 2024 . In 2018, the ratio of playgrounds to children aged 0-11 years nationally was 1:903; in 2024 the ratio is 1:820 and in 2018, the ratio of MUGAs and skate parks was 1:1,964, which improved to 1:1,711 in 2024.**
- **The reduction in play officers from 12 in 2018 to 3 in 2024 may be due to sports partnership roles.**
- **Sustained funding schemes such as the capital grant funding scheme for play and recreation provide local authorities financial assistance to develop new or refurbish existing play and recreation facilities and spaces.**

- **Play and recreation amenities targeting adolescents require more development due to the lower provision and increased population figures.**
- **Examples of good practice are documented in separate reports.**

NGO reports that address the need for outdoor play:

For inclusive play opportunities:

11. Moloney, C., de Bhailís, C., Kennan, D., Kealy, C., Quinlivan, S., Flynn, E., & Phiri, J. (2021). *Mind the Gap: Barriers to the realisation of the rights of children with disabilities in Ireland*. Ombudsman for Children's Office and Centre for Disability Law and Policy at National University College Galway. https://www.oco.ie/app/uploads/2021/03/MindTheGap_OCO_NUIG_Disability_Report.pdf

Local play policies:

Several counties have developed play regional play policies. These are usually written for a specific time frame accordingly to national and regional policy and legislation. Here some examples which are currently active policy documents:

12. Dublin City Council. (2022). *Everywhere, any day, you can Play! Dublin City Play strategy 2022-2027*. Dublin City Council Publication. https://www.dublincity.ie/sites/default/files/2022-10/Play%20Strategy%202022.pdf
13. Dún Laoghaire-Rathdown County Council. (2023). *Dún Laoghaire-Rathdown County Council Play Policy 2023-2028*. https://www.dlrcoco.ie/sites/dlrcoco/files/2023-12/dlr_Play%20Policy_ENG_SCREEN%20%284%29.pdf
14. Fingal County Council. (2022). *Space for Play—A Play Policy for Fingal*. https://www.fingal.ie/news/space-play-play-policy-fingal#:~:text=With%20Space%20for%20Play%20we,that%20we%20are%20creating%20recreation
15. Kildare County Council. (2018). *Kildare Play Strategy 2018-2028*. https://kildarecoco.ie/YourCouncil/Publications/Community/Kildare%20Play%20Strategy%2020182028.pdf
16.

Local Playground Design Strategies: Examples:

17. Leitrim Playground Strategy : https://www.leitrim.ie/council/services/community-development/playgrounds/playground-strategy-2021-2026.pdf
18. Wexford Playground Strategy: https://www.wexfordcoco.ie/sites/default/files/content/Community/Playground%20Strategy%202017-2022%20-%20adopted%2008.05.2017.pdf
19. Tipperary Playground Policy: https://www.tipperarycoco.ie/sites/default/files/2022-07/Tipperary%20Playground%20Policy.pdf

Local Outdoor Recreation Policies: Examples

20. Donegal Outdoor Recreation Policy: https://www.donegalcoco.ie/media/donegalcountyc/community/pdfs/oris/Donegal%20Outdoor%20Recreation%20Strategy%202023-2029%20-%20English%20and%20Irish.pdf
21. Wicklow: https://www.wicklow.ie/Living/Services/Community/Sport-Recreation/Play-Policy
22. Gallway Active Play Strategies: https://www.cypsc.ie/_fileupload/Documents/Resources/Galway/Active%20Play%20for%200%20%E2%80%93%203%20year%20olds%20in%20Galway%20City.pdf

Educational:

Play & Education Ireland:

23. National primary school rules (Primary Circular 11/95 'Time in School') Must provide a 'minimum' of 40 minutes daily (out of a total school day of 5 hours 40 minutes) of recreational breaktime.Department of Education. (1965). *Rules for National Schools*. https://www.into.ie/media-centre/circulars/rules-for-national-schools-1965/ & Department of Education. (2003). *Supervision For Mid-Morning and Lunchtime Breaks Primary Circula 18/03* https://circulars.gov.ie/pdf/circular/education/2003/PC18.pdf

Playful Learning /Play Based Learning in schools

Embedded in Aistear Early Childhood Curriculum Framework & Standard 6 of Síolta: National Quality Framework for Early Childhood Education:

24. https://www.aistearsiolta.ie/en/creating-and-using-the-learning-environment/aistear-and-siolta-documents/guidelines-for-good-practice-learning-and-developing-through-play-pp-56-57-and-103-106-.pdf
25. https://www.aistearsiolta.ie/en/play/resources-for-sharing/risky-play-birth-six-years-.pdf
26. **https://ncca.ie/en/primary/outdoor-learning/**
27. https://ncca.ie/en/primary/early-years-of-primary/play-in-the-early-years-of-primary/
28. https://www.aistearsiolta.ie/en/play/overview/pillar-overview-learning-through-play.pdf

School Technical Design Guidelines

29. National primary school design guidelines specify that a typical 16 classroom school with internal space of 2185 m2 ***should*** allow for outdoor yard space of 1170 m2 (ball court area of 585 m^2) and a junior soft play area of 430 m2 ((TGD-022, Primary school design guidelines, 2013).
30. Recent technical design guidelines further recommend schools adopt child-led, context based and universal design approaches to provide flexible, diverse outdoor spaces including for play (DOE, 2017). gov - Technical Guidance Documents/School Design Guides (www.gov.ie)

School Initiatives:

31. Active School Flag | More Schools, More Active, More Often
32. Green-Schools | Working Together For A Sustainable Future (greenschoolsireland.org)

Name and describe fundings bodies that support OP.

Funding to support the delivery of outdoor play is largely supported by the Government of Ireland. Some examples:

- Irish research council
- Heritage council
- EU funding for play research was received by UCC for P4Play project
- For Outdoor Recreation Infrastructure Scheme (ORIS) https://www.localgov.ie/grants-and-funding/outdoor-recreation-infrastructure-scheme-oris#:~:text=Grants%20and%20Funding-,Outdoor%20Recreation%20Infrastructure%20Scheme%20(ORIS),enhance%20existing%20outdoor%20recreational%20facilities

- DCYA: Annual Capital Grant Scheme for Play and Recreation -https://www.gov.ie/en/press-release/c5816-minister-ogorman-announces-funding-to-local-authorities-for-the-refurbishment-and-development-of-playgrounds-and-play-areas/
- Sports Capital Programme: https://www.gov.ie/en/service/d13385-sports-capital-programme/?referrer=https://www.dttas.ie/sport/english/sports-capital-programme
- https://ageandopportunity.ie/active/age-and-opportunity-active-national-grant-scheme/#desktop
- https://www.localgov.ie/grants-and-funding: Examples- Clár, Leader, Community Recognition Fund, Urban & Rural Renewal.
- The Playing Outside Grant 2021- €5.5 million for ECEC and SAC services as part of their Summer of Play initiative (DCEDIY 2021) to make changes and adaptations to transitional and outdoor spaces through the purchasing of outdoor equipment and materials, as well as security and ventilation (DECDIY 2021).
- gov - Sports Capital Programme (www.gov.ie)
- Per Cent for Art Scheme | Funding | Commissioning | Public Art
- gov - Play and Recreation Schemes (www.gov.ie)
- gov - Our Rural Future: Minister Humphreys announces €6.2 million in funding for schools, community and sports facilities nationwide (www.gov.ie)

OTHER

A govermental organisation was established 2006, but closed 2008 “National Play Resource Centre”

Country: Kosovo

Name of assessor: Artan Kryeziu

Date of assessment: 29/10/2024

Data form for outdoor play research, practice, and policy in Europe

This data form on outdoor play (OP) is to be completed in reference to the past 10 years and the country whom the assessor is representing.

OP is defined as a form of play that takes place outdoors [1]. Play is defined as voluntary engagement in an activity that is fun and/or rewarding and usually driven by intrinsic motivation. Please observe that this can refer to both children and adults. This document should not exceed two pages in total.

TERMINOLOGY

The term OP might not be directly or perfectly translated to the language/languages spoken in the country in question. Please name and describe terms that are used in country in question that could be conceptualised as or include outdoor play. Please also comment on how physical activity relates to these terms.

Note. Active outdoor play in not a common and friendly used terminology in Kosovo. Mostly we refer to recreation activities, out school activities or sport activities.

RESEARCH

Name active OP researchers and describe their research focus.

The effects of curriculum change programs in physical education according with the hours of teaching on the fitness level of children

Abstract

Introduction: the experts focused to improve the school curriculum of Physical Education, which is essential in the development of physical fitness in this age group of children. The aim: the purpose of this study is to determine if the program with more educational duration from Physical Education among children in three cities from different regions and the differences between them according to physical fitness. Material and methods: The study sample of 100 children were from children are from Pristina who follow Physical Education with 90 minutes of lessons, then 100 children from Presevo who follow Physical Education with 90 minutes of lessons, plus 45 minutes of sports activity as an elective subject, and 100 children from Struga who attend Physical Education with 135 minutes of lessons. All are evaluated with EUROFIT. Results: Based on the results of the basic statistical parameters in this study, we see that the children who followed the Physical Education curriculum of 135 minutes showed better results in expository strength, repetitive strength, and balance. As wellas based on the univariate analysis of variance (ANOVA) for the three groups, we have significant statistical differences in the indicators of expository strength, repetitive strength, and balance with significant values at the 0.000 level, therefore even through according to the post -host of the Schaffer model, differences 0.05 have been presented in the indicators of physical fitness. Conclusion: the children who have followed the program for135 days within a week,

[1] Lee, Eun-Young, Louise de Lannoy, Lucy Li, Maria Isabel Amando de Barros, Peter Bentsen, Mariana Brussoni, Lindsay Crompton, et al. "Play, Learn, and Teach Outdoors—Network (PLaTO-Net): Terminology, Taxonomy, and Ontology." *International Journal of Behavioral Nutrition and Physical Activity* 19, no. 1 (June 15, 2022): 66. https://doi.org/10.1186/s12966-022-01294-0.

the results are in favor of the children in the indicators of explosive strength, repetition strength and balance, as well as differences between children from three cities from different regions have been presented.

Describe scope of scientific publications and reports on OP stemming from country in question (please include references of seminal publications and publications that relate to gender, race, class, or climate).

Artan Kryeziu, Nagip Lenjani

Journal of Physical Education and Sport 23(2): 545-554.

http://www.efsupit.ro/images/stories/februarie2023/Art%2068.pdf

PRACTICE

Name and describe organizations (e.g., Outdoor Play Canada) that support and promote OP.

Organization in Kosovo at local level (public authority)
Parks Germia

Aim

Marking and development of the platform of the recreational trails network of Gërmia is a project and strategy which is based on the concept of sustainable mountain tourism development, with the aim of shaping health walking trails, mountain recreational, educational and tourist activities. It is a project based on field research and finding the simplest options for defining existing paths, placing signage, categorizing them and increasing safety throughout the protected area and beyond. Preserving the environment and encouraging citizens to use Gërmia as a health culture remains the main goal of the project and the goal of the Municipality of Pristina. During the implementation of the project, no new path was opened; but in coordination with the Directorate of Parks of the Municipality of Pristina and the sports community, the current amount of paths has been networked and articulated by categorizing, naming and recording them in the final thematic map of Gërmia. 11 mountain trails with a total length of 55 km have been defined and marked; the digital map of the trails has been created and the website of Gërmia has been launched. The paths are oriented according to the configuration of the terrain in order to include most of the mountainous territory of Gërmia and Lake Badovci within the network as well as the connection with the villages of Butovc, Suteskë, Novosellë, Mramor and Grashticë.

Name and describe networks (e.g., PLaTO-Net) that support and promote OP.

Reference- https://germiapark.net/

Organization in Kosovo at local level (public authority)
Rugova Outdoors
Aim
The town has extraordinary history, rich cultural heritage, great natural potentials, and always offering something new and original. Located in northwest of Kosovo and directly underneath of Albanian Alps, Peja has a longstanding history in tourism and offers balanced mix of rich history and opportunity for ongoing development of touristic offerings. For all those seeking adventures whether in unspoiled nature or just leisure, Peja provides complete services and facilities to offer comfortable holidays or short

excursions. If you are looking for ideal accommodations, authentic traditional gourmet food with all its delicacies, cultural heritage, great nightlife and attractive programs in nature for trekking and skiing, rock climbing, you have chosen the right destination for recreation, sport and entertainment. Peja is one of the most popular tourist destinations in Kosovo, and not by chance, today Peja is part of the world tourist map as a tourist destination to be crowned with the first world prize as Destination Stewardship of " Tourism for Tomorrow" by WTTC "World Travel and Tourism Council."

Name and describe networks (e.g., PLaTO-Net) that support and promote OP.

Reference- http://www.rugovahiking.com/wp-content/uploads/2015/07/ExploreKosovo-Rugova-outdoors-brochure-s.pdf

"Sharri" National Park is the first National Park announced in 1986 by the Assembly of Kosovo. The operation of the National Park began in 1995, while it was interrupted during the period 1999 - 2002 and was reactivated again in May 2002. With the new Law No. 03/L-087 (2013), the area of the Park has increased to 53,469 ha, which is spread over the territory of five municipalities: Kaçanik (4.1%), Suharekë (4.7%), Prizren (22.2%). Shtrpce (23.9%) and Dragash (45.1%). This Park is managed by the Directorate for the Administration of Sharri National Park with office in Prizren, which operates within the Environmental Protection Agency of Kosovo, namely the Ministry of Environment, Spatial Planning and Infrastructure.

Name and describe networks (e.g., PLaTO-Net) that support and promote OP.

Reference- Themelimi dhe veprimtaria.pdf (ammk-rks.net)

POLICY

Name and describe national policy documents that support OP.

Manual for non-formal education of young people through sports and physical activities in nature

Non-Formal Education through outdoor education is an organized process that takes place in parallel with the basic education and training system and is directly and indirectly related to outdoor activities. Participation is voluntary and as a result, the individual takes an active role in the learning/training process. Non-formal education offers opportunities to young people to develop their values, skills and competences, unlike the basic educational system. These skills acquired include a wide range of interpersonal, team, conflict management competencies, cross-cultural awareness, leadership, planning, organization, coordination and practical problem-solving skills, self-confidence, discipline and responsibility. The special feature of outdoor education is the fact that participants are actively involved in the entire process and activities that take place. The methods which are used and have the goal of giving young people the right tools for developing their skills and increasing their competence. Learning is a continuous process, while one of its basic characteristics is "learning by doing". This whole dynamic and hopeful process is filled with various activities that do not contradict the standard learning system, but on the contrary, as a process, it is designed in such a way as to create the environment in which the participant is the architect of development own.

Name and describe fundings bodies that support OP.

Reference- Manual for non-formal education of young people through sports and physical activities in nature
https://www.kap-ks.com/wp-content/uploads/2019/11/1.-MANUAL-ALB-v1.pdf

OTHER

Kosovo Government

Organization of sports activities for children

- National level the Government of Kosovo-Ministry of Culture, Youth and Sports-MKRS, Ministry of Education, Science and Technology and the Federation of School Sports of Kosovo., the “Towards the Mountain” project, will be realized before 2022.

Drejt Malit

On 08.02.2024 in Brezovica, the Federation of School Sports has opened the project “Towards the Mountain”. This project is about learning to ski. The first group of 1200 seventh graders from 29 municipalities of Kosovo are teaching this. Program this year, this program which continues during the summer with mountain hiking. “Drejt Malit”; has the goal of children`s health and non-formal education through physical activities. The implementation of the program is being done in partnership between the Ministry of Sports, the Ministry of Education and the Federation of School Sports of Kosovo.
Reference- https://www.mkrs-ks.org/?page=1,6,3752

Country: MONTENEGRO

Name of assessor: STEVO POPOVIC

Date of assessment: 13 OCTOBER 2024

Data form for outdoor play research, practice, and policy in Europe

This data form on outdoor play (OP) is to be completed in reference to the past 10 years and the country whom the assessor is representing.

OP is defined as a form of play that takes place outdoors [1]. Play is defined as voluntary engagement in an activity that is fun and/or rewarding and usually driven by intrinsic motivation. Please observe that this can refer to both children and adults. This document should not exceed two pages in total.

TERMINOLOGY

The term OP might not be directly or perfectly translated to the language/languages spoken in the country in question. Please name and describe terms that are used in country in question that could be conceptualised as or include outdoor play. Please also comment on how physical activity relates to these terms.

Montenegro is considered an ecological country firmly committed to environmental protection and sustainability. In this country, outdoor play (OP) can be broadly understood within the context of cultural and recreational activities in natural or open spaces. The following terms are relevant to outdoor play in the Montenegrin context. The first term, ***"igra na otvorenom (Outdoor Play),"*** refers to outdoor play activities for children and adults alike. It encompasses various playful activities in parks, beaches, mountains, and rural landscapes, which are plentiful in Montenegro. The term ***"fizička aktivnost (Physical Activity),"*** which refers to any bodily movement produced by skeletal muscles that results in energy expenditure, should also occur in this paragraph. Still, it needs to be highlighted that this term is often associated with sports, exercise, and recreational activities in the local language, many of which are done outdoors, such as hiking, swimming, and playing team sports like football or basketball in open spaces. Another term, ***"igra na livadi (Play in the Meadow),"*** must be accompanied as it is commonly used to describe unstructured outdoor play, particularly in natural settings like meadows and fields. Children often engage in this type of play, which includes running, chasing, ball games, and other forms of active physical play. It is rooted in rural traditions and is still seen in more suburban and countryside areas. Furthermore, the term ***"izlet (Outing/Excursion)"*** often involves families or groups going on a day trip to natural areas, such as national parks, mountains, or lakes. While it might not be labeled explicitly as play, these outings often involve hiking, playing games, swimming, or other recreational activities that align with the concept of outdoor play. Another term very similar to the first one is ***"sport na otvorenom (Outdoor Sports)"*** but I decided to add it as this term directly refers to organized physical activities or sports conducted outdoors. Activities like football (soccer), basketball, volleyball, hiking, mountain biking, and swimming in the Adriatic Sea are every day in Montenegro. While these are often structured forms of physical activity, they are also popular recreational activities for children and adults. The terms listed reflect how outdoor play is conceptualized in Montenegro, emphasizing a connection to nature, physical movement, and traditional social and community interaction values. Therefore, physical activity is encouraged and often an inherent part of the cultural perception of outdoor play.

[1] Lee, Eun-Young, Louise de Lannoy, Lucy Li, Maria Isabel Amando de Barros, Peter Bentsen, Mariana Brussoni, Lindsay Crompton, et al. "Play, Learn, and Teach Outdoors—Network (PLaTO-Net): Terminology, Taxonomy, and Ontology." *International Journal of Behavioral Nutrition and Physical Activity* 19, no. 1 (June 15, 2022): 66. https://doi.org/10.1186/s12966-022-01294-0.

RESEARCH

Name active OP researchers and describe their research focus.

Stevo Popovic is a full professor at University of Montenegro who has 10+ years' experience with particular focus on planning, conducting, and evaluating research studies dealing with health and exercise, which also include clinical trials. As a sports and exercise scientist he uses knowledge of how the body works to help people improve their health and sporting ability at large. However, he has also profound insight into physical anthropology, and understands the complexity of how physical activity affect the human body and its composition; but, also into social anthropology that helps to understand the social side of the same issues. With a background as a Ph.D. from the University of Novi Sad and postdoc from the University of Ljubljana (ranked 1st in Slovenia, 326th in the global 2024 rating, and scored in the top 50% across 228 research topics), as well as a teacher and research at the University of Montenegro, he has achieved the following key competencies: knowledge of teaching and the ability to design courses, project and data management, study design expertise, excellent communication skills, and dissemination skills in both written and oral etc. He currently holds several leading positions in the national and international projects, as well as leading roles and memberships in the governing bodies of professional and scientific organizations. He is a former Dean of Faculty of Sport and Physical Education and Editor-in-Chief of University of Montenegro Press, both in two mandates, former member of HEPA Europe Steering Committee, FIEPS Board of Directors member and member of Montenegrin Academy of Science and Art (Centre for Young Scientists and Artists). On the other hand, among several other positions, he is currently a Co-Director of Balkan Institute of Science and Innovation and Associate Editor in British Journal of Sports Medicine (Physical Activity and Population Health section). Authored 83 articles in peer-reviewed journals indexed by Scopus database (17% as the first, 19% as the last, 55% as the co-author and 9% as the single author; 25 documents in top citation percentiles), several books, book chapters and conference papers and abstracts. Cited >7,200 times; H-index = 22; Field Weighted Citation Impact (FWCI) = 5.16. As a supervisor or methodological consultant contributed to four PhD dissertations as well as many bachelor and master research theses. In 2020, on the Stanford/Elsevier's list of top 2% researchers globally.
Scopus profile: https://www.scopus.com/authid/detail.uri?authorId=55369012200

Describe scope of scientific publications and reports on OP stemming from country in question (please include references of seminal publications and publications that relate to gender, race, class, or climate).

Research on outdoor play (OP) in Montenegro is still developing, with limited systematic studies directly focused on this topic. However, some key themes related to outdoor activities and sports participation have been examined, including gender disparities and environmental factors like climate. A critical area of research is gender inequality in sports, where it has been found that women in Montenegro face significant barriers to participation. Studies conducted in collaboration with NGOs and international organizations have highlighted the underrepresentation of women in sports and the need for gender-sensitive policies to encourage female participation in outdoor activities. This includes barriers related to income, access to facilities, and societal norms. Climate change has also become a topic of interest in physical activity and outdoor play. As part of the Mediterranean region, Montenegro faces specific challenges related to environmental sustainability and climate resilience. Research is emerging on how climate risks, such as extreme weather, could impact outdoor play opportunities and physical activity, particularly in coastal areas like Kotor Bay. While there is no singular comprehensive body of work on OP in Montenegro, these studies contribute to understanding the broader context in which outdoor play occurs, particularly regarding gender, climate, and environmental factors. To develop this area further, more targeted research would be needed to address specific intersections of race, class, and other social factors.

PRACTICE

Name and describe organizations (e.g., Outdoor Play Canada) that support and promote OP.

Several organizations in Montenegro promote outdoor play (OP) through nature conservation, physical activity, and youth engagement, though only sometimes explicitly labeled as OP. The **National Parks of Montenegro** manage five parks (Durmitor, Biogradska Gora, Lovćen, Skadar Lake, and Prokletije), offering hiking, camping, and educational activities that promote outdoor experiences. **Mountaineering and Alpine Clubs** (e.g., Komovi, Durmitor) organize outdoor education and excursions, fostering a love for nature. The NGO **Green Home** focuses on environmental sustainability, running camps and workshops emphasizing outdoor play and conservation. **Montenegro Outdoor** promotes recreational activities like hiking and cycling, while **Youth Outdoor Montenegro** encourages youth participation in sports and environmental education. Lastly, the **Ecological Society of Montenegro** promotes sustainable living through outdoor activities and nature walks. Collectively, these organizations support outdoor play by creating environments conducive to nature exploration and physical activity.

Name and describe networks (e.g., PLaTO-Net) that support and promote OP.

While Montenegro lacks networks equivalent to PLaTO-Net, several networks indirectly promote outdoor play by focusing on environmental education, youth engagement, and sports. The **Montenegro Outdoor Coalition** is an informal network of sports clubs and nature organizations that encourage outdoor recreational activities, such as hiking and cycling, helping to foster outdoor play. The **Montenegro Youth Network (Mreža mladih Crne Gore)** works with NGOs and government bodies to engage youth in outdoor learning and recreational programs, promoting physical activity and environmental awareness. The **Adriatic Ionian Initiative (AII)** is a regional collaboration that supports sustainable tourism and outdoor activities, including youth engagement projects, often emphasizing cross-border cooperation. The **Balkan Green Network** connects environmental organizations across the region, promoting outdoor education and sustainability initiatives that involve outdoor play. **SEEYN (South East European Youth Network)** engages youth in non-formal education, including outdoor activities and play. At the same time, the **Eco-Schools Network Montenegro** integrates environmental education with outdoor activities in schools, fostering a love for nature and outdoor learning.

POLICY

Name and describe national policy documents that support OP.

Montenegro has several national policy documents that indirectly support outdoor play (OP) by focusing on physical activity, environmental education, and youth engagement. These are some key policy frameworks: **the National Strategy for Sustainable Development** (2016-2030), which emphasizes the importance of sustainable tourism, environmental protection, and public health, all of which align with promoting outdoor activities; **the National Youth Strategy** (2017-2021), which focuses on enhancing youth well-being through participation in various physical and recreational activities, including sports and outdoor programs; the **National Sports Development Strategy** (2018-2021) which aims to increase physical activity across the population, particularly among children and youth and encourages the development of outdoor sports and recreational facilities, which indirectly promotes outdoor play by making it more accessible and fostering a culture of physical activity in natural settings; **the National Strategy for Environmental Education** (2020-2030) which supports outdoor education as part of a broader effort to foster environmental responsibility. These documents highlight the importance of integrating outdoor activities into national policies on sustainability, youth engagement, and sports development, providing an indirect foundation for promoting outdoor play across Montenegro.

Name and describe fundings bodies that support OP.

There are some funding bodies that support outdoor play initiatives in Montenegro such as **UNICEF Montenegro** that is dedicated to promoting children's rights and well-being, and their programs emphasize early childhood development, social protection, and quality education with aim to address inequalities faced by children, especially those affected by poverty and exclusion, which can include access to safe outdoor play spaces; **Eureka Network** that supports research and development projects, including those that may focus on outdoor play and community development; **Marine Foundation** that promotes socio-economic development and supports educational initiatives, which can include outdoor play projects; and **local government grants** that may offer grants for community development projects, including those that enhance outdoor play facilities. These organizations can provide various forms of support, from direct funding to resources for developing outdoor play spaces.

OTHER

None.

Country: the Netherlands

Name of assessor: Manon Bloemen

Date of assessment: 14-11-2024

Data form for outdoor play research, practice, and policy in Europe

This data form on outdoor play (OP) is to be completed in reference to the past 10 years and the country whom the assessor is representing.

OP is defined as a form of play that takes place outdoors [1]. Play is defined as voluntary engagement in an activity that is fun and/or rewarding and usually driven by intrinsic motivation. Please observe that this can refer to both children and adults. This document should not exceed two pages in total.

TERMINOLOGY

The term OP might not be directly or perfectly translated to the language/languages spoken in the country in question. Please name and describe terms that are used in country in question that could be conceptualised as or include outdoor play. Please also comment on how physical activity relates to these terms.

The Dutch word for outdoor play is "buitenspelen". " Buitenspelen" is used for any type of outdoor play, from voluntary engagement to guided play. It is a very common word/term in the Netherlands. Terms that also may be used are "buitenshuis spelen" (playing outside the house), " naar buiten gaan" (going outside). You can also say " buiten spelen" as 2 words instead of 1 word. This is used in fe sentences like 'we are going to play outside – we gaan buiten spelen'. So " playing outside" is similar to " buiten spelen".<br><br>Outdoor play is part of physical activity, similar to sport or active transport.<br><br>If in the Netherlands we use the terms risky play ("risicovol spelen" or " avontuurlijk spelen", playground or play area ("speeltuin" or " speelplek"), natural playground (Natuurspeeltuin), we mostly refer to playing outside. We do have inside playgrounds, but than we mostly use the term "binnen speeltuin", which means inside playground. We also use "samen spelen" or "inclusief spelen" to refer to inclusive play, and sometimes we also mean outside inclusive play with this.

RESEARCH

Name active OP researchers and describe their research focus.

Gerben Helleman (University of applied sciences De Hague, PhD student outside play)<br>Sannne de Vries (University of applied sciences De Hague, Healthy Lifestyle in a Supporting Environment)<br>Rianne Janssen (PhD student Inclusive play, P4Play)<br>Kirsten Visser (Utrecht University. Urban Geography at the Faculty of Geosciences. In her research, Visser looks at the situation and development of children and young people from a geographical perspective)<br>Heidi Lesscher (Utrecht University) neurobiology of Behaviour research group at the Faculty of Veterinary Medicine at Utrecht University. the role of play in the development of brain and behavior)<br>Ryan Beekhuizen (HU University of Applied Sciences Utrecht, PhD student inclusive play)

[1] Lee, Eun-Young, Louise de Lannoy, Lucy Li, Maria Isabel Amando de Barros, Peter Bentsen, Mariana Brussoni, Lindsay Crompton, et al. "Play, Learn, and Teach Outdoors—Network (PLaTO-Net): Terminology, Taxonomy, and Ontology." *International Journal of Behavioral Nutrition and Physical Activity* 19, no. 1 (June 15, 2022): 66. https://doi.org/10.1186/s12966-022-01294-0.

Eline Bolster (HU University of Applied Sciences Utrecht. Inclusive play, inclusive outside play, behavrioual change, pediatric physical therapy, physical fitness and physical activity, children with disabilities and participation)
Manon Bloemen (HU University of Applied Sciences Utrecht. Inclusive play, inclusive outside play, behavrioual change, pediatric physical therapy, physical fitness and physical activity, disabilities and participation)

Describe scope of scientific publications and reports on OP stemming from country in question (please include references of seminal publications and publications that relate to gender, race, class, or climate).

Barriers, facilitators and solutions for active inclusive play for children with a physical disability in the Netherlands: a qualitative study.
van Engelen L, Ebbers M, Boonzaaijer M, Bolster EAM, van der Put EAH, Bloemen MAT.BMC Pediatr. 2021 Aug 28;21(1):369. doi: 10.1186/s12887-021-02827-5.PMID: 34454470

Buitenspelen in Nederland, Factsheet. 2023. Mullier Instituut. Buitenspelen in Nederland

Kantar Public. Onderzoek buitenspelen 2022. Een onderzoek onder ouders en kinderen. Onderzoek buitenspelen 2022 Een onderzoek onder ouders en kinderen

Onderzoek buitenspelen 2024. Een onderzoek onder ouders en kinderen. Conceptrapport - Buitenspelen kinderen 2024 - Jantje Beton

PRACTICE

Name and describe organizations (e.g., Outdoor Play Canada) that support and promote OP.

Jantje Beton. Jantje Beton | Elk kind moet kunnen buitenspelen, elke dag!
Platform Ruimte voor de Jeugd Ruimte voor de Jeugd | Voor spelen, participatie, zelfstandige mobiliteit en een gezonde ontwikkeling
Stichting het Gehandicapte Kind. Geen kind zonder vriendjes | Stichting het Gehandicapte Kind
Samenspeelnetwerk. SamenSpeelNetwerk | Onbeperkt spelen.
LOS, Brancheorganisatie voor buitenspelen, vrije tijd en natuur. Homepage - Vereniging LOS
Branchevereniging spelen en bewegen. Brancheverenigingі Spelen & Bewegen
Buitenonderwijs Nederland. Buitenonderwijs Nederland

Name and describe networks (e.g., PLaTO-Net) that support and promote OP.

Platform Ruimte voor de Jeugd Ruimte voor de Jeugd | Voor spelen, participatie, zelfstandige mobiliteit en een gezonde ontwikkeling

Samenspeelnetwerk. SamenSpeelNetwerk | Onbeperkt spelen.

POLICY

Name and describe national policy documents that support OP.

Beleid over buitenspelen bij Nederlandse gemeenten<br><br>https://allesinbeweging.net/storage/2265/2020-Mulier---Verkenning-Buitenspelen-EU-en-NED.pdf<br><br>Sporten en bewegen voor kinderen | Sport en bewegen | Rijksoverheid.nl

Name and describe fundings bodies that support OP.

Samenspeelfonds SamenSpeelFonds | SamenSpeelNetwerk

OTHER

Country: Norway

Name of assessor: Ellen Beate Hansen Sandseter

Date of assessment: 29th October 2024

Data form for outdoor play research, practice, and policy in Europe

This data form on outdoor play (OP) is to be completed in reference to the past 10 years and the country whom the assessor is representing.

OP is defined as a form of play that takes place outdoors [1]. Play is defined as voluntary engagement in an activity that is fun and/or rewarding and usually driven by intrinsic motivation. Please observe that this can refer to both children and adults. This document should not exceed two pages in total.

TERMINOLOGY

The term OP might not be directly or perfectly translated to the language/languages spoken in the country in question. Please name and describe terms that are used in country in question that could be conceptualised as or include outdoor play. Please also comment on how physical activity relates to these terms.

Outdoor play (*utelek*) in the Norwegian language is something that is usually associated with childhood/adolescence. Among children, (outdoor) play can be both physically active or more sedentary, but especially for the younger ages, play usually includes being in movement (young children shift between play types quite often). Outdoor play is strongly emphasized in the curriculum/educational framework of the kindergarten/preschool and school, and outdoor play is considered a valuable learning arena for children and youth. All Norwegian kindergartens/preschools spend a considerable amount of time providing outdoor play, and there is a large amount of nature- and outdoor kindergartens (*friluftsbarnehage, utebarnehage, naturbarnehage*) that have outdoor play and activities as their primary focus, spending (almost) all their time outdoors (on playgrounds, in parks, in nature environments) all year through. There are also primary schools practicing outdoor school (*uteskole*), moving parts of the teaching outdoors, primarily into nature environments. Although much of the content in uteskole can be free play, main the focus here would be learning in the outdoors, led by a teacher.

For older ages, outdoor play would typically be outdoor activities or non-competitive outdoor sports that people do just for the fun of it. As such, many of these activities are part of what we call (active) *frilusftliv*. Even though friluftsliv (first named by the novelist Henrik Ibsen in the 1850'ies) also includes *just being* in nature, the Norwegian practice of it traditionally includes activity of some kind, and it is closely connected to "Bildung" and health promotion. Therefore, political documents focusing on friluftsliv, health and education emphasize "outdoor play/activity" for all age groups of Norwegian society.

There is a distinction in the Norwegian language between non-competitive physical activity (*fysisk aktivitet, trening, uteaktivitet* etc.) and competitive physical activity (*idrett, sport*). Non-competitive physical activity is a self-initiated activity that can take place both indoors and outdoors, and for the latter, this would align closely with the concept of outdoor play.

Norway's tradition is that all citizens shall have easy access to spaces for play, physical activity, and recreational activities. Therefore, all playgrounds, even those owned by kindergartens, schools, housing companies, etc., are publicly available for anyone to access and use during afternoons, evenings, and

[1] Lee, Eun-Young, Louise de Lannoy, Lucy Li, Maria Isabel Amando de Barros, Peter Bentsen, Mariana Brussoni, Lindsay Crompton, et al. "Play, Learn, and Teach Outdoors—Network (PLaTO-Net): Terminology, Taxonomy, and Ontology." *International Journal of Behavioral Nutrition and Physical Activity* 19, no. 1 (June 15, 2022): 66. https://doi.org/10.1186/s12966-022-01294-0.

weekends. The law of common access (*allemannsretten*) also secures all citizens the right to access and spend time in any natural areas across the country, even if privately owned.

RESEARCH;

Name active OP researchers and describe their research focus.

Rune Storli (children, outdoor play, friluftsliv, health, learning, play environments), Ole Johan Sando (children, outdoor play, health, learning, development, play environments), Rasmus Kleppe (children, outdoor play, health, learning, development, play environments), Kathrine Bjørgen (children, outdoor play, health, learning, sustainability, environmental awareness/climate), Ellen Beate Hansen Sandseter (children, outdoor play, health, learning, development, play environments), Jostein Rønning Sanderud (children, outdoor play, friluftsliv, learning, exploration, play environments), Vidar Ulset (children, outdoor play, learning, development), Merete Lund Fasting (children and youth, outdoor play, friluftsliv, well-being, life mastery), Vegard Gundersen (children and youth, outdoor play, friluftsliv), Ingunn Fjørtoft (children and youth, outdoor play, friluftsliv, learning, development), Lise Kjønniksen (children and youth, outdoor play, friluftsliv, learning, development), Knut Løndal (children and youth, outdoor play, friluftsliv, learning, development), Eivind Aadland (children and youth, outdoor play, friluftsliv, health, development), Steffen Tangen (children, outdoor play, learning, development), Alexander Olsen (children, outdoor play, learning, development), Kirsti Gurholt Pedersen (children, youth, adults outdoor play, friluftsliv, well-being, gender, ethnicity), Kari Helene Danielsen (children and youth, outdoor play, physical activity), Karen Vibeke Klepsvik (children, outdoor play, friluftsliv, development, sustainability), Jon Anders Græsli (children, outdoor play, learning), Olav Bjarne Lysklett (children, outdoor play, learning, development), Kari-Anne Jørgensen-Vittersø (children, outdoor play, friluftsliv, development, learning), Bjørg Oddrun Hallås (children and youth, outdoor activity, learning).

Describe scope of scientific publications and reports on OP stemming from country in question (please include references of seminal publications and publications that relate to gender, race, class, or climate).

Fjørtoft, I. The Natural Environment as a Playground for Children: The Impact of Outdoor Play Activities in Pre-Primary School Children. Early Childhood Education Journal 29, 111–117 (2001). https://doi.org/10.1023/A:1012576913074

Fasting, M.L. (2013). Vi leker ute! - en fenomenologisk hermeneutisk tilnærming til barns lek og lekesteder ute. Novus Forlag

Fasting, M. L. (2015). Klatring i trær og hyttebygging: – om barns lek og lekesteder ute. Nordisk barnehageforskning, 10. https://doi.org/10.7577/nbf.1431

Ulset, V., Vitaro, F., Brendgen, M., Bekkhus, M., & Borge, A. I. H. (2017). Time spent outdoors during preschool: Links with children's cognitive and behavioral development. Journal of Environmental Psychology, 52, 69-80. https://doi.org/https://doi.org/10.1016/j.jenvp.2017.05.007

Sando, O. J. (2019). The outdoor environment and children's health: a multilevel approach. *International Journal of Play*, *8*(1), 39–52. https://doi.org/10.1080/21594937.2019.1580336

Sandseter, E. B. H., Storli, R., & Sando, O. J. (2020). The dynamic relationship between outdoor environments and children's play. *Education 3-13*, *50*(1), 97–110. https://doi.org/10.1080/03004279.2020.1833063

Wold, Line C., Skår, Margrete, Øian, Hogne (2020). Barn og unges friluftsliv. Norsk institutt for naturforskning (NINA)

Sandseter, E. B. H., & Storli, R. (2020). Barnehagens fysiske inne- og utemiljø. Inspirasjon til lek. Universitetsforlaget.

Line Camilla Wold, Vegard Gundersen, Odd Inge Vistad (2024). Bli med ut og lek! Hva skjedde under koronaperioden? Norsk institutt for naturforskning (NINA)

Online research-based resource for play environments in kindergartens: https://barnehagemiljo.no/

There are several more scientific articles, reports, books etc., but the above is a small selection.

PRACTICE

Name and describe organizations (e.g., Outdoor Play Canada) that support and promote OP.

A selection:

- Friluftsrådenes landsforbund – also with many local departments across the country, *The National Association of Outdoor Councils* (FL) is a nationwide cooperation body for inter-municipal outdoor councils. FL's main task is to work to strengthen general *friuftsliv* through its own initiatives and by supporting the work of affiliated outdoor councils.
- Norsk Friluftsliv - is the joint organization for the 19 large voluntary outdoor recreation organisations, with one million members and close to 5,000 teams and associations. Works to solve tasks and coordinate matters of common interest, promote these to the authorities and other relevant target groups, strengthen the position and spread of outdoor life in society, etc.
- Barnehageforum – an organization that provides material and support for kindergartens on outdoor play, etc.
- Naturfagsenteret (naturfag.no) – works to support schools, kindergartens etc. with knowledge and material to support outdoor play and activities, with an emphasis on nature, sustainability etc.
- Forskerfrø (forskerfrø.no) - a specialized department of Naturfagsenteret that provides outdoor play and nature activities for children/kindergartens.
- Tverga – organization that works actively on influencing the processes of designing an providing outdoor spaces for children and youth to play and be active. Provides resources and have political influence.
- Den Norske Turistforening (DNT) – Norway's largest friluftsliv organization, also with many local departments across the country, provides opportunities for practicing friluftsliv for all age groups.
- Barnas turlag – a specialized department of DNT that provides outdoor play and nature activities for children.

Name and describe networks (e.g., PLaTO-Net) that support and promote OP.

- Nasjonalt nettverk for fysisk fostring – network for practice and research in physical education and health (including outdoor play) for kindergarten teacher training.
- Nettverk for Natur- og gårdsbarnehager – network for all outdoor/nature kindergartens and farm kindergartens in Norway
- Nettverk for forskning på kroppsøving og idrettsfag – networ for research on pgysical education and sport (includning outdoor play/activities) in schools in Norway.

POLICY

Name and describe national policy documents that support OP.

A selection:

- Report to the Storting (White Paper) no. 18: Friluftsliv – Natur som kilde til helse og livskvalitet (Nature as a source of health and quality of life)
- Report to the Storting (White Paper) no.15: Folkehelsemeldinga – Nasjonal strategi for utjamning av sosiale helseforskjellar (People's health report - National strategy for equalizing social health differences)
- Sammen for aktive liv. Handlingsplan for fysisk aktivitet 2020-2029 (Together for active lives. Action plan for physical activity)
- Læreplan i kroppsøving (KRO01-05) (Curriculum for Primary, Lower Seconadry and Upper Secundary Education in Norway)
- Rammeplan for barnehagen (Framework Plan for Kindergartens)

Name and describe funding bodies that support OP.

There are no funding bodies that support only OP, but OP could be one of the themes they fund in these funding bodies e.g.: The Research Council of Norway, Stiftelsen Damm, Utdanningsdirektoratet, NordPlus, NordForsk, Miljødirektoratet, Statsskog, Gjensidigestiftelsen, Olav Thon stiftelsen, Sparebankstiftelsene, and many of the fylkeskommuner (county municipality) in Norway provide funding,

OTHER

Country: POLAND

Name of assessor: KAMIL MACIASZEK

Date of assessment: 14.11.2024

Data form for outdoor play research, practice, and policy in Europe

This data form on outdoor play (OP) is to be completed in reference to the past 10 years and the country whom the assessor is representing.

OP is defined as a form of play that takes place outdoors [1]. Play is defined as voluntary engagement in an activity that is fun and/or rewarding and usually driven by intrinsic motivation. Please observe that this can refer to both children and adults. This document should not exceed two pages in total.

TERMINOLOGY

The term OP might not be directly or perfectly translated to the language/languages spoken in the country in question. Please name and describe terms that are used in country in question that could be conceptualised as or include outdoor play. Please also comment on how physical activity relates to these terms.

In Poland, the term "outdoor play" does not have a direct literal translation. The closest equivalents are "zabawa na świeżym powietrzu" (play in the fresh air) or "aktywność na zewnątrz" (outdoor activity). Frequently used terms also include "aktywność fizyczna na świeżym powietrzu" (physical activity in the fresh air) or "zabawy ruchowe na dworze" (movement-based outdoor play), particularly in the context of education and health promotion.<br><br>Physical activity is a key component of these terms and is regarded as a crucial element for the healthy development of children and adolescents. Outdoor play is actively promoted as a means of fostering physical, mental, and social development while also serving as a preventative measure against overweight and obesity among children.<br><br>A term used in some primary schools and preschools is "zabawa gratami" (play with loose parts), referring to play activities involving loose elements such as recycled or repurposed materials. This concept was pioneered by the GratoSfera Foundation.

RESEARCH

Name active OP researchers and describe their research focus.

Active OP researchers in Poland:<br>- PhD Małgorzata Wróblewska (Academy of Physical Education, Warsaw): Specializes in research on the impact of physical activity, including outdoor play, on children's motor development.<br><br>- PhD Marta Makuch (Adam Mickiewicz University, Poznań): Focuses on the role of play in children's development, including outdoor play, and its impact on social integration.<br>- MA Kamil Maciaszek: A practitioner with extensive experience in implementing free play in primary schools and preschools in Poland. Creator of the GratoSfera project, which promotes outdoor play as a

[1] Lee, Eun-Young, Louise de Lannoy, Lucy Li, Maria Isabel Amando de Barros, Peter Bentsen, Mariana Brussoni, Lindsay Crompton, et al. "Play, Learn, and Teach Outdoors—Network (PLaTO-Net): Terminology, Taxonomy, and Ontology." *International Journal of Behavioral Nutrition and Physical Activity* 19, no. 1 (June 15, 2022): 66. https://doi.org/10.1186/s12966-022-01294-0.

key element of mental health and youth development. The project emphasizes free play as a means to build resilience and self-confidence in children and young people through games and outdoor activities.

- PhD Anna Brzezińska (Adam Mickiewicz University, Poznań): Developmental psychologist whose research includes the role of education and play, including outdoor activities, in children's development.

- PhD Marta Bogdanowicz (University of Gdańsk): Developmental and educational psychologist specializing in the effects of play on children's psychomotor development.

- PhD Ewa Pisula (University of Warsaw): Psychologist focusing on children's development, particularly the significance of play in their lives.

- PhD Małgorzata Żytko (University of Warsaw): Pedagogue researching preschool and early childhood education methods, with a particular emphasis on the role of outdoor play.

- PhD Anna Odrowąż-Coates (Maria Grzegorzewska University, Warsaw): Sociologist investigating the impact of the external environment on children's development, including the importance of free outdoor play.

Describe scope of scientific publications and reports on OP stemming from country in question (please include references of seminal publications and publications that relate to gender, race, class, or climate).

Scope of Scientific Publications: Publications on outdoor play (OP) in Poland include studies on the impact of physical activity on children's physical and mental health, as well as the factors influencing outdoor play participation, such as the availability of green spaces, support from parents and teachers, and economic and environmental considerations.

Sample Publications:

1. Loose Parts Play Erasmus+ Project Research Report: "Making the Case for Loose Parts Play in Primary Schools Across Europe." A study focusing on the impact of loose parts play on school activity. [Link](https://www.loosepartsplayproject.eu/our-research)
2. Kowalik, I., Wróblewska, M. (2018). "The Importance of Outdoor Play for Children's Health and Development" – A study on the role of outdoor play in the context of physical and mental health.
3. Makuch, M. (2020). "Play as the Foundation for Children's Social Integration" – An article analyzing how differences in access to green spaces affect children's participation in outdoor activities.

PRACTICE

Name and describe organizations (e.g., Outdoor Play Canada) that support and promote OP.

Organizations Supporting and Promoting Outdoor Play (OP):

• GratoSfera Foundation – GratoSfera is an innovative educational project implemented in Polish schools, particularly in the Gdańsk region, aimed at supporting children's development through free, active play. The project was designed to introduce "loose parts play" into school spaces, using simple, recycled materials such as wood, fabrics, and other materials that children can use in any way they wish. This approach enables creative play, the development of social and physical skills, and enhances mental

well-being. GratoSfera also provides training and resources for educators and organizes community events to promote outdoor play as essential for healthy child development.

GratoSfera was developed by Kamil Maciaszek in collaboration with local partners, including the Department of Social Development of the Gdańsk City Hall and the Gdańsk Academy of Physical Education and Sport. Supported by the city of Gdańsk as a priority project, it currently operates in at least 33 primary schools, reaching over 10,000 students. GratoSfera also offers support and training for teachers, as well as workshops and events promoting free play as an essential element of children's healthy development.

The initiative is gaining support due to its impact on improving social integration, reducing stress, and supporting mental health following the COVID-19 pandemic.

• Fundacja Dzieci i Natura – An organization dedicated to promoting outdoor play and organizing educational programs that bring children closer to the values associated with nature and outdoor activities.

• Fundacja "Czas na Las" – The foundation runs the "Mysikrólik" Forest Kindergarten, which follows the curriculum of preschool education (as per the Polish Ministry of Education), focusing on spontaneous movement and free outdoor play.

• Rezerwat Dzikich Dzieci – The first adventure playground in Poland promoting free play, children's autonomy during play, and their independence in choosing play materials and methods.

• Ministry of Climate and Environment – In collaboration with the Polish Athletics Association and National Parks, the ministry carried out the "EKOatleci Parki Narodowe" project, promoting outdoor activities for students, teachers, and families.

• Institute of Sport – National Research Institute – The institute acts as the national operator of the School Sports Club Program, engaging children and youth in regular sports activities, developing their physical fitness, and promoting outdoor activities.

• Fundacja "SEMAFOR" – An organization that supports children's development through play, emphasizing its significance in physical, emotional, intellectual, and social growth.

• Outdoor Education Po Polsku – An organization promoting experiential education, offering comprehensive planning for outdoor activities for children aged 3 to 18, with a focus on safety and development through outdoor play.

Name and describe networks (e.g., PLaTO-Net) that support and promote OP.

Networks Supporting and Promoting Outdoor Play (OP):

- Project Space for Children – A collaborative network between schools, non-governmental organizations, and local authorities aimed at promoting outdoor activities and the development of playgrounds.
- Coalition for Environmental Education – A network focused on environmental education and promoting outdoor play as an essential part of children's development.
- Polish Association for Physical Education (PSWF) – Promotes physical activity among children and youth, including outdoor play, by organizing conferences, meetings, and workshops for educators.

POLICY

Name and describe national policy documents that support OP.

- National Health Program (NHP) 2021-2025 – mentions the importance of outdoor physical activities as part of preventive health actions, highlighting their impact on children's physical and mental health.
- Rural Development Program (RDP) for 2014-2020 – promotes the development of outdoor play areas in rural areas, emphasizing the health and social benefits of these activities.
- National Strategy for Regional Development (NSRD) 2030 – focuses on creating child-friendly public spaces that encourage outdoor play and physical activities.

Name and describe fundings bodies that support OP.

- Ministry of Sport and Tourism – offers funding for projects that promote physical activity, including outdoor play, as a method of preventing health problems among children and youth.
- National Fund for Environmental Protection and Water Management (NFOŚiGW) – supports ecological projects, including the development of green public spaces and playgrounds, which indirectly contribute to promoting outdoor play.
- Civic Initiatives Fund (FIO) – provides funding for NGOs implementing projects that encourage children's outdoor activities as a means of community integration and development.
- Local government budgets - for example, the Department of Social Development of the City of Gdansk.

OTHER

Additional information: There is growing interest in Poland in OP as a preventive measure to combat mental health problems and overweight and obesity among children.

Country: **Scotland**

Name of assessor: **Dr Avril Johnstone**

Date of assessment: **09/10/2024 – 18/10/2024**

Data form for outdoor play research, practice, and policy in Europe

This data form on outdoor play (OP) is to be completed in reference to the past 10 years and the country whom the assessor is representing.

OP is defined as a form of play that takes place outdoors [1]. Play is defined as voluntary engagement in an activity that is fun and/or rewarding and usually driven by intrinsic motivation. Please observe that this can refer to both children and adults. This document should not exceed two pages in total.

TERMINOLOGY

The term OP might not be directly or perfectly translated to the language/languages spoken in the country in question. Please name and describe terms that are used in country in question that could be conceptualised as or include outdoor play. Please also comment on how physical activity relates to these terms.

In Scotland, we use similar terms to those adopted in Canada with active play and outdoor play commonly used, and often interchangeably. Nature play and risky are used less frequently in Scottish research policy or practice.

A term more specific to the Scottish context is outdoor play and learning, which is increasingly used in an educational context (early learning and childcare and primary school settings). It is the belief that children and young people should have the opportunity to play and learn outside regularly. The emergence of this term is tied closely to recent Scottish Government policies, particularly the expansion of the number of free childcare hours parents are entitled to. This policy could only be achieved by encouraging more children to play outdoors when in childcare. Increasing the number of outdoor play and learning opportunities in early learning and childcare and primary schools has been the most significant way we have promoted outdoor play in Scotland. More recently, the UNCRC Rights of the Child have been enshrined in Scot's law meaning our Government and other public bodies have a duty to uphold the right of the child including Article 31, the Child's Right to Play.

Outdoor play (/active play) is seen as a type of physical activity distinct of sport, active transport, PE etc. These terms would usually be reserved for children and young people. The term recreational physical activity would be used for adults, however, it is often difficult to differentiate leisure or recreational physical activity to sport.

RESEARCH

Name active OP researchers and describe their research focus.

University of Glasgow:

1. Dr Avril Johnstone's research focuses on the role of active outdoor play, nature and/ or physical activity on children's health and wellbeing.
2. Dr Paul McCrorie is co-lead of a nature play and learning research programme. His research interests also focus on the built, natural, and social environmental determinants of physical activity.
3. Dr Anne Martin is co-lead of a nature play and learning research programme. Her research interests also include child and adolescent healthy weight interventions and improving sleep and mental health.
4. Dr Oliver Traynor's research focuses on nature-based play and learning on children's health and development.
5. Dr Arlene McGarty's research focuses on active play in children with neurodevelopmental conditions.

University of Strathclyde:

[1] Lee, Eun-Young, Louise de Lannoy, Lucy Li, Maria Isabel Amando de Barros, Peter Bentsen, Mariana Brussoni, Lindsay Crompton, et al. "Play, Learn, and Teach Outdoors—Network (PLaTO-Net): Terminology, Taxonomy, and Ontology." *International Journal of Behavioral Nutrition and Physical Activity* 19, no. 1 (June 15, 2022): 66. https://doi.org/10.1186/s12966-022-01294-0.

6. Professor John J Reilly is lead of the Active Healthy Kids Scotland Report Card, which includes an indicator on active play. His broader research interests relate to physical activity and obesity in children and adolescents.

Glasgow Caledonian University:

7. Professor John McKendrick's research interests intersect poverty, play and children's environment.

University of Stirling:

8. Dr Lily Fitzgibbon's research focuses on the behavioural, cognitive, and emotional processes involved in adventurous and risky play.
9. Professor Gregory Mannion's research focuses on outdoor play and learning and learning for sustainably.

Queen Margaret University:

10. Stacey Marko's PhD research explores how urban and rural contexts influence children's outdoor play opportunities and experiences.

Describe scope of scientific publications and reports on OP stemming from country in question (please include references of seminal publications and publications that relate to gender, race, class, or climate).

Currently no national survey provides evidence of how much time children are spending in active play for more than 2 hours per day. The last data source was from the 2016 Scottish Health Survey, which was re-analysed for Scotland's Active Health Kids Report Card in 2018. It reported 26% of 2–15-year-olds participated in active play for ≥2 hrs/day on a weekday (28% for boys, 24% for girls), rising to 40% at the weekend (42% for boys, 38% for girls).

Most other studies have aimed to understand the impact of specific outdoor play interventions (e.g. nature-based childcare, Active Play programme) or how outdoor play could be promoted.

The Active Healthy Kids Report Card has reported (Bardid et al, 2022) on sex and socioeconomic inequalities and secular trends. McGarty has done important work on children with neurodevelopmental conditions. Generally, in Scotland, we try to understand differences in socioeconomic status and sex as a minimum. We have not had the data to understand differences by race or climate.

Seminal publications (in no particular order):

1. Zucca C, McCrorie P, Johnstone A, Chambers S, Chng NR, Traynor O, Martin A. Outdoor nature-based play in early learning and childcare centres: Identifying the determinants of implementation using causal loop diagrams and social network analysis. *Health & Place*. 2023 Jan 1;79:102955.
2. Martin A, Clarke J, Johnstone A, McCrorie P, Langford R, Simpson SA, Kipping R. A qualitative study of parental strategies to enable pre-school children's outdoor and nature experiences during COVID-19 restrictions. *Health & Place*. 2023 Jan 1;79:102967.
3. Traynor O, Martin A, Johnstone A, Chng NR, Kenny J, McCrorie P. A Low-Cost Method for Understanding How Nature-Based Early Learning and Childcare Impacts Children's Health and Wellbeing. *Frontiers in Psychology*. 2022 Jun 23;13:889828.
4. Johnstone A, Martin A, Cordovil R, Fjørtoft I, Iivonen S, Jidovtseff B, Lopes F, Reilly JJ, Thomson H, Wells V, McCrorie P. Nature-based early childhood education and children's social, emotional and cognitive development: A mixed-methods systematic review. *International journal of environmental research and public health*. 2022 May 13;19(10):5967.
5. Johnstone A, McCrorie P, Cordovil R, Fjørtoft I, Iivonen S, Jidovtseff B, Lopes F, Reilly JJ, Thomson H, Wells V, Martin A. Nature-based early childhood education and children's physical activity, sedentary behavior, motor competence, and other physical health outcomes: a mixed-methods systematic review. *Journal of Physical Activity and Health.* 2022 May 10;19(6):456-72.
6. Bardid F, Tomaz SA, Johnstone A, Robertson J, Craig LC, Reilly JJ. Results from Scotland's 2021 report card on physical activity and health for children and youth: Grades, secular trends, and socio-economic inequalities. *Journal of Exercise Science & Fitness.* 2022 Oct 1;20(4):317-22.
7. McGarty A, Jones N, Rutherford K, Westrop S, Sutherland L, Jahoda A, Melville C. Feasibility of the Go2Play Active Play intervention for increasing physical and social development in children with intellectual disabilities. *Pilot and Feasibility Studies*. 2021 Dec;7:1-9.

8. Johnstone A, Hughes AR, Bonnar L, Booth JN, Reilly JJ. An active play intervention to improve physical activity and fundamental movement skills in children of low socio-economic status: feasibility cluster randomised controlled trial. *Pilot and feasibility studies*. 2019 Dec;5:1-3.

9. Johnstone A, Hughes AR, Martin A, Reilly JJ. Utilising active play interventions to promote physical activity and improve fundamental movement skills in children: a systematic review and meta-analysis. *BMC Public Health*. 2018 Dec;18:1-2.

Under Review

1. McCrorie P, Johnstone A, Nicholls N, Keime M, Jidovtseff B, Martin A. Risky outdoor play in the early years: How are parental and practitioner perceptions of danger and benefits associated with young children's outdoor play experiences? *International Journal of Play* (Under Review).

Seminal reports (in no particular order):

1. FitzGibbon L., & Dodd, H The State of Play in Scotland 2023. Play Scotland. 2023. Retrieved from https://www.playscotland.org/resources/print/PS00105-PS-State-of-Play-in-Scotland-2023-Digital-links.pdf?plsctml_id=24133
2. Mannion, G., Ramjan, C., McNicol, S., Sowerby, M. and Lambert, P. (2023) Teaching, Learning and Play in the Outdoors: a survey of provision in 2022. NatureScot Research Report 1313. Retrieved from https://www.nature.scot/doc/naturescot-research-report-1313-teaching-learning-and-play-outdoors-survey-provision-scotland-2022
3. Watson M, Sarica S, Parkinson J, Mitchell R, Wason D. COVID-19 Early Years Resilience and Impact Survey (CEYRIS). Report 2 – Play and learning, outdoors and social interactions in children in Scotland aged 2–7 during COVID-19. Edinburgh: Public Health Scotland; 2020. Retrieved from https://publichealthscotland.scot/media/3105/report-2_play-and-learning-outdoors-and-social-interaction_ceyris.pdf

PRACTICE

Name and describe organizations (e.g., Outdoor Play Canada) that support and promote OP.

Organisations with National Remit:

- The Scottish Government has several departments that promote active and outdoor play. Notably, Active Scotland are responsible for Scotland's Physical Activity Strategy and the Child and Families directorate has led much of Scotland's Outdoor Play and Learning work
- Play Scotland is the lead organisation for the development and promotion of play in Scotland.
- Inspiring Scotland is a national charity focused on improving the lives of people and communities across Scotland. The Thrive Outdoors programme sits within Inspiring Scotland's priority areas. Thrive Outdoors' vision is for a sustainable Scotland where our children and young people can play, learn, and thrive outdoors.
- Education Scotland is the national body for supporting quality and improvement of learning and teaching in Scottish education. They have published national guidance relevant to outdoor play.
- Learning Through Landscapes Scotland is a dedicated charity to enhancing outdoor learning and play for children.
- Public Health Scotland lead and support work across Scotland to prevent disease, prolong healthy life and promote health and wellbeing. They continue to promote active and outdoor play.
- Early Years Scotland is Scotland's leading national specialist organisation that supports our youngest children from pre-birth to 5 years of age. Much of their work centres on promoting play.
- The Care Inspectorate are the national regulator for care services in Scotland. They have played an important role in promoting outdoor play across Scotland's Early Learning and Childcare Settings.
- Actify CIC provide national active play training to teachers, playworkers and other practitioners.
- NatureScot aim to enhance Scotland's nature and inspire the people of Scotland to care more about it. They have published toolkits, guidance and research on outdoor play and learning.

Organisations with Local Remit:

In addition, there are also local organisations from the public, private and charity sectors who are promoting outdoor play. This includes our Early Learning and Childcare settings and primary schools. These organisations are integral to delivering the policies and agendas set out by our Government and other national organisations.

Name and describe networks (e.g., PLaTO-Net) that support and promote OP.

Scotland's National Outdoor Play and Learning Position Statement is a broad coalition of researchers, policymakers and practitioners who are committed to working together to embed playing and learning outdoors as an everyday activity and a fundamental part of growing up in Scotland.

POLICY

Name and describe national policy documents that support OP.

- Scotland's Play Strategy, Play Scotland aims to promote play (including outdoor play) as a life-enhancing daily experience for all children and young people: in their homes, nurseries, schools and communities
- United Nations Convention on the Rights of the Child (UNCRC) have been incorporated into Scot's Law meaning our Government and other public bodies have a duty to uphold the right of the child including Article 31, the Child's Right to Play.
- Play Sufficiency Assessment, Scottish Government requires planning authorities to assess the sufficiency of outdoor play opportunities for children in their local communities. The play sufficiency assessments inform the preparation of local development plans. This is closely tied to the incorporation of the UNCRC into Scot's Law and Scotland's new National Planning Framework (NPF4) which has a section to encourage, promote and facilitate spaces and opportunities for outdoor play, recreation and sport.
- Scotland's National Physical Activity Policies, Scottish Government include The Active Scotland Outcomes Framework, which describes Scotland's ambitions for sport and physical activity. This is supported by Scotland's new National Delivery Plan for Physical Activity and Outcomes Framework. Active play and recreational activity are referenced several times a way of increasing population level physical activity.
- The National practice guidance for early years in Scotland, Education Scotland outlines the importance of play and play pedagogy. They also have a play pedagogy toolkit to be used alongside the national guidance.
- Out to Play, Scottish Government provides guidance for early learning and childcare settings and practitioners on how to access outdoor spaces to create safe, nurturing and inspiring outdoor learning experiences
- My World Outdoors, Care Inspectorate highlights the benefits of outdoor play for children attending early learning and childcare.
- NatureScot has a range of toolkits, guidance documents and research related to outdoor play.

Name and describe fundings bodies that support OP.

Funding to support the delivery of outdoor play is largely supported by the Scottish Government and local authorities. Scottish Government has an Outdoor Community Play fund managed by Inspiring Scotland. There are other funding bodies and trusts, however, these have a broader remit than just outdoor play. For example, The Robertson Trust, National Lottery Community Funding, Corra Foundation.

Outdoor play research would tend to be supported by traditional Scottish or UK research bodies, including UKRI, NIHR or CSO. Glasgow City Council has also funded research and The Baily Thomas Charitable Fund supports research with a focus on those with a disability.

OTHER

Country: Slovakia

Name of assessor: Peter Bakalár based on the data provided by 17 stakeholders via online data form.

Date of assessment: 15.11.2024

Data form for outdoor play research, practice, and policy in Europe

This data form on outdoor play (OP) is to be completed in reference to the past 10 years and the country whom the assessor is representing.

OP is defined as a form of play that takes place outdoors [1]. Play is defined as voluntary engagement in an activity that is fun and/or rewarding and usually driven by intrinsic motivation. Please observe that this can refer to both children and adults. This document should not exceed two pages in total.

TERMINOLOGY

The term OP might not be directly or perfectly translated to the language/languages spoken in the country in question. Please name and describe terms that are used in country in question that could be conceptualised as or include outdoor play. Please also comment on how physical activity relates to these terms.

There is no consensus in Slovakia in literal translation of the term 'outdoor play' among relevant stakeholders. Based on the analysis of the translation proposals from 17 relevant stakeholders, the most common translation was 'hra vonku' (singular form) or 'hry vonku' (plural form) and the second most used translation was 'outdoorové aktivity' followed by 'hry v prírode' and 13 other possible but less frequent translations with only one most direct translation as 'outdoorová hra'. This situation may lead to different understanding of the term 'outdoor play' in Slovak context. And, finding the consensus may seems to be difficult task since the incorporation of the loanword 'outdoor/outdoorový' from English word 'outdoor' is accepted by some and rejected by others.

In Slovak, the term 'von/vonku' can be translated by using word 'outside' rather than 'outdoor/outdoors' and refers to the close surroundings rather than being in the remote forest, mountains or water bodies environment. On the other hand, the loan word 'outdoor/outdoorový' has more broader meaning including all natural surroundings with its emphasis on the term 'nature'.

Therefore, in the Slovak context, the backtranslation of the term 'outdoor play' would bring more apt terms such 'outside play' or 'playing outside' rather than 'outdoor play'.

At the same time, there is tendency in education academic sphere to incorporate the English loan word 'outdoor/outdoorový' for all outdoor activities including playing outside. In this case, the term 'outdoorové aktivity' serves as a superordinate term including concepts such as 'hra(y) vonku' [outside play/playing outside].

In general, Slovakia has long tradition in promoting outdoor activities. The term 'hra(y) vonku' [outside play/playing outside] is usually used for children and adolescents. The terms 'turistika' and more recently the loan word 'outdoorové aktivity' are used for all age groups. Interestingly, as it was pointed out by one stakeholder, the term 'outdoor sports' is officially translated as 'športy v prírode' which can be back translated to English as 'sports in nature'. Using this logic, the term 'outdoor play' could be translated in

[1] Lee, Eun-Young, Louise de Lannoy, Lucy Li, Maria Isabel Amando de Barros, Peter Bentsen, Mariana Brussoni, Lindsay Crompton, et al. "Play, Learn, and Teach Outdoors—Network (PLaTO-Net): Terminology, Taxonomy, and Ontology." *International Journal of Behavioral Nutrition and Physical Activity* 19, no. 1 (June 15, 2022): 66. https://doi.org/10.1186/s12966-022-01294-0.

Slovak as ‘hry v prírode’ [play in nature] which was suggested by three stakeholders as a possible translation.

To conlude, there is a need for reaching the terminological consensus regarding the translation of the term ‘outdoor play’ in Slovak language.

RESEARCH

Name active OP researchers and describe their research focus.

Peter Bakalár (adolescents, outdoor activities, outdoor play, and health), Vladimír Fedorko (pre-school children, outdoor education), Milica Sabol (pre-school children, outdoor education), Janka Sýkorová (pre-school children, outdoor education), Karol Görner (adolescents, outdoor activities, and health), Stanislava Straňavská (adolescents, outdoor activities, and health), Jaroslav Kompán (children, adolescents, outdoor activities, and health)

Describe scope of scientific publications and reports on OP stemming from country in question (please include references of seminal publications and publications that relate to gender, race, class, or climate).

Bakalár P. First report card on physical activity for children and adolescents in Slovakia: a comprehensive analysis, international comparison, and identification of surveillance gaps. Archives of Public Health, 2024 (outcome of the project Global Matrix 4.0 within which the literature for Active Play indicator was searched and evaluated and substantial research gap in this indicator was identified, https://doi.org/10.1186/s13690-024-01241-4, https://www.activehealthykids.org/4-0/).

Fedorko V. Outdoorová edukácia v materskej škole. Prešov: Prešovská univerzita v Prešove, 2020 (outcome of the Erasmus+ project ‘Taking learning outdoors – supporting the skills of pre-school managers in outdoor education and care - TAKE ME OUT II. - Happy childhood happens outside - STEP HIGHER’, https://www.pulib.sk/web/kniznica/elpub/dokument/FedorkoV5, https://erasmus-plus.ec.europa.eu/projects/search/details/2019-1-SK01-KA201-060775)

Stráňavská, S, Görner, K. Vzťah adolescentov k outdoorovým aktivitám. Banská Bystrica: Belianum, 2017 (outcome of the VEGA project ‘Miesto turistiky a športovo-pohybových aktivít v prírodnom prostredí v spôsobe života seniorov’ focused on the topic of outdoor activities, https://www.ftvsz.umb.sk/fakulta-telesnej-vychovy-sportu-a-zdravia/veda-a-vyskum/publikacie/book-19044/vztah-adolescentov-k-outdoorovym-aktivitam.html)

Kompán J, Adamčák, Š, Belás, M, Babiar, M, Görner, K, Pačuta, S. Outdoorové aktivity, športy a špecifiká pobytu v prírode. Banská Bystrica: Belianum, (outcome of the project KEGA 044UMB-4/2016 ‘Outdoorové aktivity, športy a špecifiká pobytu v prírode’ focused on the outdoor activities of adolescents, https://sclib.svkk.sk/sck01/Record/000585645#description)

PRACTICE

Name and describe organizations (e.g., Outdoor Play Canada) that support and promote OP.

Outdoor Institute – organization operating in the field of free time animation and important player in the field of development of methods of experiential pedagogy and outdoor activities. Working with all age groups.

Živica – civic organization that uses experiential education, the goal of which is a deep inner experience supporting people in discovering their own potential and sensitive perception of their surroundings. The organization organizes educational festival Hurá von! [Hooray out!] focused on the outdoor education. Working with all age groups.

Plusko – civic organization that uses experiential pedagogy. Working with children, adolescents and young adults.

Slovenský skauting (Slovak Scouting) - is one of the largest educational organizations for children and adolescents in Slovakia using modern and attractive forms of a quality scouting program for all age groups. Main focus is on outdoor activities.

Domka – Združenie saleziánskej mládeže (Domka - Salesian Youth Association) - is a civic association with a nationwide scope. With more than 8,000 members, mainly children and adolescents and young adults between the ages of 6 and 30, it is one of the largest youth organizations in Slovakia. Using outdoor activities in their programs.

eRKo (Movement of Children's Christian Communities) – operates throughout Slovakia and has more than 6,000 members. Its mission is to create gatherings for children to grow together in joy. The main activities are: creating meetings for children, summer camps, training animators and all-Slovak educational and charitable children's projects such as Dobrá novina, DOBROčiny and MINIdigi.

Klub Pathfinder (The Pathfinder Club) – is a civic association of children, adolescents and young adults of the scout type, which are organized in sections led by adult leaders or young leaders, who prepare for them various interesting activities aimed at meaningful use of free time.

Wachumba – is a mindset community that brings together children and adolescents, children, seasoned experiential pedagogy instructors and professionals from various outdoor activities and sports. They organize experience camps, adaptation stays, outdoor schools, ski courses and school trips.

INAK – organization focused on introduction of innovative approaches using activation methods and ICT in teaching, creation of didactic materials, project management, organization of training and methodological training, as well as other activities in the field of innovative education, supporting the development of human resources. Realising projects focusing on kindergartens: https://takemeoutproject.eu/ and https://www.selforschools.eu/sk/

Strom života (The Tree of Life) – as an environmental and non-profit organization they try to educate children from an early age, develop their personality and lead to the protection and improvement of the environment.

Name and describe networks (e.g., PLaTO-Net) that support and promote OP.

Klub slovenských turistov (The Club of Slovak Tourists) – the umbrella organization for organizations and individual members active in outdoor activities. In Slovakia, the term 'turistika' has mainly meaning as outdoor activities. The organization is not specifically devoted to support 'outdoor play' but through the support of outdoor activities they do indirectly support the outdoor play.

Asociácia detských lesných klubov (Association of Children's Forest Clubs) – is a nationwide organization covering entities focused on raising and educating children in regular contact with nature.

TAKE ME OUT network through the Week Out campaign (https://takemeoutproject.eu/sk/o-kampani-tyzden-vonku/) implemented twice a year since 2021, over 10,000 children from several countries have been involved so far.

Špirála (The Spiral) – is a national network of organizations dedicated to environmental education and training. It is a non-governmental, non-profit organization established as an interest association of legal entities.

Sosna (The Pine Tree) – organization with the mission to connect and activate people with the aim of spreading ecological solutions at the local and regional level. They contributed to the shift of several rural regions towards sustainability through the development plans. They have run several successful campaigns.

POLICY

Name and describe national policy documents that support OP.

Štátny vzdelávací program pre predprimárne vzdelávanie v materských školách (The State Educational Program For Pre-School Education in Kindergartens) – https://www.minedu.sk/data/att/96d/24534.b6f65c.pdf

Vzdelávacie štandardy pre vzdelávaciu oblasť Zdravie a pohyb (Educational Standards for the Educational Field Health and Movement) – https://www.minedu.sk/data/files/11834_zdravie-a-pohyb.pdf

Koncepcia športu 2022 – 2026 (The Sport Concept for Years 2022 – 2026) - chapter 2.2 Projects to increase physical activity of population, chapter 5.1 Development of local, regional and school sports grounds - the concept here mainly deals with the support of physical activities in nature, the development of public sports grounds in nature, cities and villages, but it is not precisely defined as outdoor play (https://www.minedu.sk/data/files/11170_koncepciasportu2022.pdf)

Outdoorové aktivity vo výchove mimo vyučovania (The Outdoor Activities in After School Education Activities) – https://archiv.mpc-edu.sk/sites/default/files/publikacie/t_volkova_outdoorove_aktivity_vo_vychove_mimo_vyucovania.pdf

Štandardy outdoorovej edukácie (The Outdoor Education Standards) – outcome of the Erasmus+ project 'Taking learning outdoors – supporting the skills of pre-school managers in outdoor education and care - TAKE ME OUT II. - Happy childhood happens outside - STEP HIGHER', https://takemeoutproject.eu/sk/materialy/)

Name and describe fundings bodies that support OP.

Ministerstvo školstva, výskumu, vývoja a mládeže Slovenskej republiky (The Ministry of Education, Research, Development and Youth of the Slovak Republic) – provides funds for schools.

Ministerstvo cestovného ruchu a športu Slovenskej republiky (Ministry of Tourism and Sports of the Slovak Republic) – newly established in 2024. Provides funds for sports organizations.
NIVAM - Národný inštitút vzdelávania a mládeže (National Institute of Education and Youth) – organization funded by the The Ministry of Education, Research, Development and Youth of the Slovak Republic.

European Commission grant schemes (e.g. Erasmus+).

Grant schemes of the private companies.

OTHER

Country: Republic of Slovenia

Name of assessor: Shawnda Morrison and Nika Bezjak

Date of assessment: Week of Oct 14 – 21, 2024

Data form for outdoor play research, practice, and policy in Europe

This data form on outdoor play (OP) is to be completed in reference to the past 10 years and the country whom the assessor is representing.

OP is defined as a form of play that takes place outdoors [1]. Play is defined as voluntary engagement in an activity that is fun and/or rewarding and usually driven by intrinsic motivation. Please observe that this can refer to both children and adults. This document should not exceed two pages in total.

TERMINOLOGY

The term OP might not be directly or perfectly translated to the language/languages spoken in the country in question. Please name and describe terms that are used in country in question that could be conceptualised as or include outdoor play. Please also comment on how physical activity relates to these terms.

The word for outdoor play in Slovenia is ”igra na prostem”, which means any type of play taking place outdoors. Most of outdoor play is taking place in the urban environment, such as playgrounds (”igrišče”). These are divided into school playground (”šolsko igrišče”) or school courtyard (”šolsko dvorošče”), children's playgrounds (”otroško igrišče”) and outdoor playground (”zunanje igrišče”). Outdoor play also takes place in the natural environment (”naravno okolje”) or in a forest (”gozd”). The concept of outdoor play is simultaneously associated with risky play (“tvegana igra”) and free play (“prosta igra”) in the outdoor environment and nature play (“igra v naravi”).

Slovenia emphasizes outdoor play to a greater extent in the early stages of child development, while in later years (primary school) outdoor education (”izobraževanje na prostem”), outdoor teaching (”pouk na prostem”) and forest pedagogy (”gozdna pedagogika”) become more prominent. Most of these approaches involve regular and repetitive access to nature and encourage self-directed learning. The organization of a school in nature ("šola v naravi") is also mandatory in primary schools, i.e. a special organizational form of education which takes place at least three days away from the pupil's place of residence and is usually happening in a natural environment. Although the nature school has an educational purpose, pupils also engage in various physical activities outdoors (skiing, hiking, swimming, biking, etc..) and spend most of their free time playing outside. At the same time, it provides an opportunity to develop authentic relationships, nature skills, and environmental awareness.

Recreation and leisure activities in nature (“zunanja rekreacija” or “rekreacija v naravi”) are highly developed in Slovenia, which encourages movement and physical activity (“telesna dejavnost”) in nature or on green areas (“zelene površine”), such as parks, walking paths, meadows, forests, sports islands (“otoki športa”) and trim tracks (“trim steze”) etc. Although recreation plays an important role in promoting health and general well-being, we excluded it as it lacks the basic elements of play and is more identified with outdoor sport than with outdoor play in Slovenia.

RESEARCH

Name active OP researchers and describe their research focus.

1. Marjanca Kos (Educational Science and Biology) University of Ljubljana, Faculty of Education
2. Irena Hergan (Educational Science and Biology, Special Didactics and Geography) University of Ljubljana, Faculty of Education

[1] Lee, Eun-Young, Louise de Lannoy, Lucy Li, Maria Isabel Amando de Barros, Peter Bentsen, Mariana Brussoni, Lindsay Crompton, et al. “Play, Learn, and Teach Outdoors—Network (PLaTO-Net): Terminology, Taxonomy, and Ontology.” *International Journal of Behavioral Nutrition and Physical Activity* 19, no. 1 (June 15, 2022): 66. https://doi.org/10.1186/s12966-022-01294-0.

3. Darja Dimec Skribe (Educational Science, Special Didactics) University of Ljubljana, Faculty of Education
4. Mateja Videmšek and Marjeta Kovač (Educational Science, Sport) University of Ljubljana, Faculty of Sport
5. Miha Marinšek (Sport, Kinesology – pedagogical aspect (sports education, training, motor learning) University of Maribor, Faculty of Education
6. Vita Žlender and Ina Škulje Erjavec (Urbanism and Landscape Architecture), UIRS - Urban planning institute of Republic of Slovenia
7. Jana Kozamernik adn Simon Koblar (Urbanism), UIRS - Urban planning institute of Republic of Slovenia

Describe scope of scientific publications and reports on OP stemming from country in question (please include references of seminal publications and publications that relate to gender, race, class, or climate).

See attached papers. We have also included some student thesis work. Slovenia does not have a long history researching in the outdoor play area, so we found several students' works which reference key papers in the area within Slovenia. Titles are translated to English in Brackets below:

Bell, K. (2023). Igralnica v gozdu kot alternativa gibanju na prostem (Playgrounds in the woods as an alternative to outdoor activities). *Realka*, *28*, 102–106. http://www.realka.si/wp-content/uploads/2023/07/28.%C5%A1tevilka.pdf

Gomboc, K. (2016). Back to nature: exploring the potential for implementing the Norwegian idea of outdoor days in the Slovenian school system. *Revija za elementarno izobraževanje*, *9*(4), 123–138. http://www.dlib.si/details/URN:NBN:SI:doc-JBOUVY8M

Gomboc, K. (2018). Prosta igra: norveška obveza - slovenska priložnost. (Free Play: Norwegian Obligation - Slovenian Opportunity) *Razredni pouk*, *20*(1), 33–38. http://www.dlib.si/details/URN:NBN:SI:doc-7L7NEBE5

Hočevar, N. (2021). *Možnosti otrok za tvegano igro v Sloveniji = Opportunities for risky play in Slovenia* [Magistrsko delo – Master's thesis]. http://pefprints.pef.uni-lj.si/7068/

Ocepek, E. (2024). *Analiza igre in učenja na prostem z elementi tvegane igre (Analysis of outdoor play and outdoor learning with elements of Risky play): diplomsko delo (Bachelor's thesis)*. [E. Ocepek]. https://dk.um.si/IzpisGradiva.php?id=87058

Pirc, S. (2017). *Pomen preživljanja prostega časa otrok na igrišču za njihov gibalni razvoj (Importance of children's free time on a playground for their movement development): diplomsko delo (Bachelor's thesis)* [[S. Pirc]]. https://dk.um.si/IzpisGradiva.php?id=68854

Plevnik, M. (2021). Benefits at outdoor play and exercise for children and youth = Prednosti gibanja in igre v naravnem okolju za otroke in mladostnike. *Premagajmo posledice ukrepov omejitve gibanja: zbornik povzetkov*, 50–51. http://ovg.si/wp-content/uploads/2021/10/Zbornik-OVG-2021_PRELIMINARNI.pdf

Mohorič, M. (2017). *Pomen igre predšolskih otrok v naravnem okolju za učenje začetnega naravoslovja (The Importance of Play in Natural Environments for Preschool Children's Early Science Learning): magistrsko delo (*Master's thesis) [[M. Mohorič]]. http://pefprints.pef.uni-lj.si/id/eprint/4428

Kos, M., Mohorič, M., Praprotnik, L., Tunnicliffe, S. D., & Torkar, G. (2022). Nature play as a way of learning biology in early years. *ERIDOB 2022: 13th Conference of European Researchers in Didactics of Biology*, 44. https://2022.eridob.org/images/pdfs/abstracts.pdf

Mednarodna znanstvena in strokovna konferenca z mednarodno udeležbo „Otrok v gibanju" (Child in motion), 11. (2021). *Premagajmo posledice ukrepov omejitve gibanja: zbornik povzetkov: 11. Mednarodna znanstvena in strokovna konferenca z mednarodno udeležbo „Otrok v gibanju"(Overcoming the Consequences of Movement Restrictions: Book of Abstracts: 11th International Scientific and Professional Conference with International Participation 'Children in Motion), [Portorož, 4. - 6. oktober 2021] = The lockdown aftermath*. Znanstveno-raziskovalno središče, Annales ZRS; = Science and Research Centre, Annales ZRS. https://www.zrs-kp.si/wp-content/uploads/2021/11/Zbornik-povzetkov-OVG-2021_spletna-izdaja.pdf

PRACTICE

Name and describe organizations (e.g., Outdoor Play Canada) that support and promote OP.

1. *The Olympic Committee of Slovenia* (OKS) promotes not only elite sports but also a 'Sports for All' section, where everyone can find a suitable event for themselves. For younger audiences, it particularly promotes outdoor events that encourage active play and non-competitive sports activities
2. *Slovenia Outdoor* is an organization that promotes physical activity and sports tourism in Slovenia (active holidays in Slovenia). It primarily promotes recreational physical activities that people enjoy during their leisure time, such as water adventures, adventure parks, and more.
3. *The CŠOD* - Center for School and Extracurricular Activities – this organisation was established to support education, sports, science, and culture. It offers various programs designed with a holistic approach, promoting socialization and spending free time in nature
4. *ŠUS* – Sports Union of Slovenia is the largest national sports for all organization which connects different sports organizations, associations and clubs working in the field of sports recreation and physical activity for all. The common goal is to promote healthy lifestyle and active use of leisure time.
5. *ZŠRS Planica* (Institute of Sport of the Republic of Slovenia Planica) - is a public institution with a mission to support sports for children and youth

Name and describe networks (e.g., PLaTO-Net) that support and promote OP.

1. *Active Healthy Kids Slovenia*- Established in 2015, this research network includes expertise from diverse backgrounds, including the Play Indicator.
2. *Prezneca* project: The project is conducted by the Urban Planning Institute of the Republic of Slovenia and spans the period from 2020 to 2024. The project aims to expand the availability of green spaces within urban areas and to promote outdoor physical activities for all generations. (*http://prezenca.uirs.si/en-us/*)
3. *APOLE* (*Adventurous Play and Outdoor LEarning*) is a three-year international project that began in Slovenia in the 2022/2023 period. The project aims to incorporate elements of risky play and outdoor learning. (*https://apole.eu/)*
4. Program *Going out for Health* ("Ven za zdravje") is an extension of the *Going out for Health 2* and *1* programs, established to support the planning of green spaces that encourage physical activity among residents. It focuses on raising awareness, providing information, and educating municipalities and regions on the importance of adequate access to green spaces for regular and everyday physical activity. (*https://venzazdravje.uirs.si/en-us/)*

POLICY

Name and describe national policy documents that support OP.

1. *Uradni List*- this is a national database which documents every law or declaration passed at the National Assembly, and available for public searching from their online portal. We searched this database and found some examples of laws which help facilitate outdoor play at the local community level, in terms of safe playground, out for health, the green system in towns and villages (can be things like laws requiring every school to have a secure play area, or bicycle racks, or greenspace, or walking paths, etc).
2. Most of documents are supported by *Ministry of Education, Science and Sports, Ministry of Health, Ministry of Environment and Spatial Planning*

Name and describe funding bodies that support OP.

1. *Foundation for Sport*: The Sports Foundation is an organization responsible for securing continuous funding sources, managing public funds, and coordinating various interests in sports

(This includes funding the construction of outdoor (otoki športa, trim steze) and children's playgrounds and organizing sports events that promote outdoor play<br>2. APOLE project is co-funded by the *Erasmus KA220 program* (Cooperation partnerships in school education)<br>3. ARIS (Public Agency for Research and Innovation of the Republic of Slovenia) is founding project PREZENCA (Project: V5-2232)

OTHER

Slovenia has several 'Regions' which may have their own regional or local experts in the "outdoor play" space, but we have not included that level of detail here. Please let us know if that would be of interest to the team if needed.

Country: Spain

Name of assessor: Silvia Veiga-Seijo

Date of assessment:01-11 to 17-11

Data form for outdoor play research, practice, and policy in Europe

This data form on outdoor play (OP) is to be completed in reference to the past 10 years and the country whom the assessor is representing.

OP is defined as a form of play that takes place outdoors [1]. Play is defined as voluntary engagement in an activity that is fun and/or rewarding and usually driven by intrinsic motivation. Please observe that this can refer to both children and adults. This document should not exceed two pages in total.

TERMINOLOGY

The term OP might not be directly or perfectly translated to the language/languages spoken in the country in question. Please name and describe terms that are used in country in question that could be conceptualised as or include outdoor play. Please also comment on how physical activity relates to these terms.

The literal translation of outdoor play is 'juego fuera', although Spanish would probably use more words to get that meaning, such as 'juego al aire libre' or 'jugar fuera de casa' (playing outside home). It includes the types of play that happens outside the home of children. Usually, it is associated with playing in playgrounds, play parks, nature, streets, etc. Playing outdoors is also associated with free play, that is, play that is led by the children without structure or rules. Play for the sake of play would also represent this terminology of outdoor play.
It is also important to highlight that while play in English is both a name and a verb, in Spanish the verb is "jugar" and the name is "juego". This is an important element to consider for searching for articles or projects.
In Spain, the idea of play or outdoor play is also different to the meaning of physical activity. This would also depend on the onto-epistemology adopted by the researcher, practitioner or organization. The idea of outdoor play highlights the play activities that are intrinsic and motivated by the child.
Depending on the context and philosophy, outdoor play is utilized from an instrumental perspective, such as, for learning, development or health.

RESEARCH

Name active OP researchers and describe their research focus.

Silvia Veiga-Seijo (play, community), Laura Camas Garrido, José Luis Linaza (play, development, right), Inma Marín (play, learning), Silvia Sánchez Serrano, Mª del Rosario González, Rosario Ortega, Petra Mª Pérez
Andres Paya Rico (play, right, popular/traditional play) and Bantulà Janot have important research in the right to play analyzing play for the sake of play and analysis in policies.
Group of researchers (Natalia Rivas, Inés Viana, Marta Canabal, Silvia Veiga Seijo): play for the sake of playing in schoolyards.

[1] Lee, Eun-Young, Louise de Lannoy, Lucy Li, Maria Isabel Amando de Barros, Peter Bentsen, Mariana Brussoni, Lindsay Crompton, et al. "Play, Learn, and Teach Outdoors—Network (PLaTO-Net): Terminology, Taxonomy, and Ontology." *International Journal of Behavioral Nutrition and Physical Activity* 19, no. 1 (June 15, 2022): 66. https://doi.org/10.1186/s12966-022-01294-0.

Amanda Fernades is located at the Barcelona Institute of Global Health and I have identified some research on the topic.

Describe scope of scientific publications and reports on OP stemming from country in question (please include references of seminal publications and publications that relate to gender, race, class, or climate).

Researcher expert in involving children for creating play-friendly communities:
Veiga-Seijo, S., Kantartzis, S., & Jackson, J. (2023). *Creating a Play-Friendly Community with Children: A Report on a Participatory-Ethnographic Research*. Queen Margaret University.

Research project transforming playgrounds in schoolyards: https://www.transformandopatios.org/en
Rivas-Quarneti, N., Viana-Moldes, I., Veiga-Seijo, S., Canabal-López, M., & Magalhaes, L. (2024). Politicizing Children's Play: A Community Photovoice Process to Transform a School Playground. *The American Journal of Occupational Therapy*, *78*(4), 7804185100.

Zych, I., Ortega-Ruiz, R., & Sibaja, S. (2016). Children´ s play and affective development: affect, school adjustment and learning in preschoolers/El juego infantil y el desarrollo afectivo: afecto, ajuste escolar y aprendizaje en la etapa preescolar. *Journal for the Study of Education and Development*, *39*(2), 380-400.

Romera, E. M., Ortega, R., & Monks, C. (2008). Impacto de la actividad lúdica en el desarrollo de la competencia social. *International Journal of Psychology and Psychological Therapy*, *8*(2), 193-202.

Jover Olmeda, G., Camas Garrido, L., Martín-Ondarza Santos, M. D. P., & Sánchez Serrano, S. (2019). La contribución del juego infantil al desarrollo de habilidades para el cambio social activo.

Camas, L., del Prado Martín-Ondarza, M., & Sánchez-Serrano, S. (2022). Game over? Perceptions of children's and adolescents' play and leisure during the COVID-19 lockdown. *International Journal of Play*, *11*(3), 311-326.

Payà Rico, A., & Bantulà Janot, J. (2021). Children's right to play and its implementation: A comparative, international perspective. *Journal of new approaches in educational research*, *10*(2), 279-294.

Janot, J. B., & Rico, A. P. (2020). The right of the child to play in the national reports submitted to the Committee on the Rights of the Child. *International Journal of Play*, *9*(4), 400-413.

RICO, A. P. (2013). Juego, educación y aprendizaje. La actividad lúdica en la pedagogía infantil. *Bordón*, 13-18.

Payá Rico, A. (2020). El juego popular y tradicional en la historia de la educación española contemporánea.

Payà Rico, A., & Bantulà Janot, J. (2019). Building a System of Indicators to Evaluate the Right of a Child to Play. *Children & Society*, *33*(1), 13-23.

Fernandes A, Krog NH, McEachan R, Nieuwenhuijsen M, Julvez J, Márquez S, de Castro M, Urquiza J, Heude B, Vafeiadi M, Gražulevičienė R, Slama R, Dedele A, Aasvang GM, Evandt J, Andrusaityte S, Kampouri M, Vrijheid M. Availability, accessibility, and use of green spaces and cognitive development in primary school children. Environ Pollut. 2023 Oct 1;334:122143. doi: 10.1016/j.envpol.2023.122143. Epub 2023 Jul 7. PMID: 37423460.

An European review that could be useful:
Fernandes A, Avraam D, Cadman T, Dadvand P, Guxens M, Binter AC, Pinot de Moira A, Nieuwenhuijsen M, Duijts L, Julvez J, De Castro M, Fossati S, Márquez S, Vrijkotte T, Elhakeem A, McEachan R, Yang T, Pedersen M, Vinther J, Lepeule J, Heude B, Jaddoe VWV, Santos S, Welten M, El Marroun H, Mian A, Andrušaitytė S, Lertxundi A, Ibarluzea J, Ballester F, Esplugues A, Torres Toda M, Harris JR, Lucia Thorbjørnsrud Nader J, Moirano G, Maritano S, Catherine Wilson R, Vrijheid M. Green spaces and

respiratory, cardiometabolic, and neurodevelopmental outcomes: An individual-participant data meta-analysis of >35.000 European children. Environ Int. 2024 Aug;190:108853. doi: 10.1016/j.envint.2024.108853. Epub 2024 Jun 28. PMID: 38963986.

PRACTICE

Name and describe organizations (e.g., Outdoor Play Canada) that support and promote OP.

These can be both networks and organisations.

Observatorio del Juego Infantil de España: https://observatoriodeljuego.es

Asociación Internacional Juego España: https://www.jugaresunderecho.org/

Fundación Educación y Desarrollo: https://www.fundacioneducacionydesarrollo.org/
AIJU: https://www.aiju.es/en/

Fundación Meniños, somos a nosa infancia: https://meninos.org/es/inicio/

Grupo ABD: https://abd.ong/causas/infancia-y-familia/

Fundación INJUCAM: https://injucam.org/

Fundación Crecer Jugando: https://crecerjugando.org/nosotros/

Asociación Española de Fabricante de Juguetes: https://www.aefj.es/

National Center of Ambiental Education (Ministry for the ecological transition and demographic challenge): https://www.miteco.gob.es/es/ceneam.html

Name and describe networks (e.g., PLaTO-Net) that support and promote OP.

Spanish Network for Outdoor Physical Education: https://www.educacionynaturaleza.com/

Clean cities Spain: https://spain.cleancitiescampaign.org/

Red Española por una Infancia Activa y Saludable (Active Healthy Kids Spanish Network): https://retokids.org/

POLICY

Name and describe national policy documents that support OP.

There is not a specific policy about play in Spain.

Protecting the school break through play: Important manifest created by Observatorio del Juego Infantil de España
https://observatoriodeljuego.es/manifiesto-para-proteger-los-recreos/

Name and describe fundings bodies that support OP.

OTHER

Country: Sweden

Name of assessor: Professor Marie Löf, Associate Professor Christine Delisle Nyström

Date of assessment: 30th October 2024

Data form for outdoor play research, practice, and policy in Europe

This data form on outdoor play (OP) is to be completed in reference to the past 10 years and the country whom the assessor is representing.

OP is defined as a form of play that takes place outdoors [1]. Play is defined as voluntary engagement in an activity that is fun and/or rewarding and usually driven by intrinsic motivation. Please observe that this can refer to both children and adults. This document should not exceed two pages in total.

TERMINOLOGY

The term OP might not be directly or perfectly translated to the language/languages spoken in the country in question. Please name and describe terms that are used in country in question that could be conceptualised as or include outdoor play. Please also comment on how physical activity relates to these terms.

Outdoor play is possible to translate directly into Swedish. In Swedish it would be "utelek" or "aktiv utelek". However, even though it is a term that is easy to translate and understand in Swedish they are not commonly used. In the Swedish context, it is common to emphasize being outdoors both in the home and preschool environments.

RESEARCH

Name active OP researchers and describe their research focus.

To the best of our knowledge, there are no well-defined OP researchers in Sweden that have conducted research in this field for a long time that we know of; however, there are related projects. Here is a list:

1. Professor Anders Raustorp, University of Gothenburg. His research covers physical activity and health in children and includes projects related to physical activity, outdoor environment and schoolyards for preschool-aged children.
2. Dr Susan Jane Waite, Jönköping University. We do not know her, but she is the only researcher with a Swedish affiliation on the publication by Lee et al Lee EY, Play, Learn, and Teach Outdoors-Network (PLaTO-Net): terminology, taxonomy, and ontology. Int J Behav Nutr Phys Act. 2022 Jun 15;19(1):66. Her research seems to be focused on outdoor learning, thus related to outdoor play.
3. Associate Professor Anna Karin Lindqvist and Stina Rutberg, Luleå Technical University. They have long and extensive expertise from research in active transportation in school-aged children and developed programs for promoting biking during the winter season. Thus, they have research in health promotion outdoors, but not outdoor play.
4. Professor Marie Löf and Associate Professor Christine Delisle Nyström are leading the Swedish arms of ongoing international projects (e.g., SUNRISE, GAC-PAQ) where the questionnaires include questions on time outdoors and reasons for not allowing children to play outdoors; however, we have not published any data from these studies to date. We also have

[1] Lee, Eun-Young, Louise de Lannoy, Lucy Li, Maria Isabel Amando de Barros, Peter Bentsen, Mariana Brussoni, Lindsay Crompton, et al. "Play, Learn, and Teach Outdoors—Network (PLaTO-Net): Terminology, Taxonomy, and Ontology." *International Journal of Behavioral Nutrition and Physical Activity* 19, no. 1 (June 15, 2022): 66. https://doi.org/10.1186/s12966-022-01294-0.

questionnaire data in some previous studies in children aged 4-17 years; however, there are no publications on outdoor play to date.

Describe scope of scientific publications and reports on OP stemming from country in question (please include references of seminal publications and publications that relate to gender, race, class, or climate).

Wallenberg N, Lindberg F, Thorsson S, Jungmalm J, Fröberg A, Raustorp A, Rayner D. The effects of warm weather on children's outdoor heat stress and physical activity in a preschool yard in Gothenburg, Sweden. Int J Biometeorol. 2023 Dec;67(12):1927-1940.<br><br>Richardson, T., Waite, S., Askerlund, P., Almers, E., Hvit Lindstrand, S. (2023). How does nature support early language learning?: A systematic literature review.<br><br>Waite, S., Prince, H. (2022). Editorial: Child, place, and others: interactions that support outdoor learning Journal of Adventure Education and Outdoor Learning, 22(4), 275-277.<br><br>Savolainen E, Lindqvist AK, Mikaelsson K, Nyberg L, Rutberg S. Children's active school transportation: an international scoping review of psychosocial factors. Syst Rev. 2024 Jan 30;13(1):47.<br><br>Rutberg S, Henriksson M, Andersson M, Palstam A, Lindqvist AK. My Way to School Through a Camera Lens: Involving Children to Inform a Policy Recommendation on Active School Travel. Health Promot Pract. 2024 Jun 6:15248399241255376.

PRACTICE

Name and describe organizations (e.g., Outdoor Play Canada) that support and promote OP.

Friluftsfrämjandet (Swedish Outdoor Association). It is a non-profit association promoting an outdoor lifestyle (https://www.friluftsframjandet.se/om-oss/in-english/).

Name and describe networks (e.g., PLaTO-Net) that support and promote OP.

None

POLICY

Name and describe national policy documents that support OP.

National level

- ***Rekommendationer för fysisk aktivitet och stillasittande*** (Recommnedations for physical activity ande sedentary behaviour)
    - (https://www.folkhalsomyndigheten.se/contentassets/106a679e1f6047eca88262bfdcbeb145/riktlinjer-fysisk-aktivitet-stillasittande.pdf)
    - Here it is mentioned through the guidelines that being outdoors contributes to more physical activity and children’s development.
- ***Varje rörelse räknas – hur skapar vi ett samhälle som främjar fysisk aktivitet*** (Every movement counts – how do we create a society that promotes physical activity)
    - Swedish Government 2023
    - https://www.regeringen.se/contentassets/8ab754ea529f4fa5a6caaa00581948bd/sou-2023_29.pdf

- Here there is mention of the importance of being outdoors contributes to children's physical activity.

- ***Sveriges friluftslivsmål*** (Sweden's outdoor lifestyle goal)
    - Swedish Environmental Protection Agency
    - https://www.naturvardsverket.se/amnesomraden/friluftsliv/sveriges-friluftlivsmal/#:~:text=M%C3%A5let%20specificeras%20i%20propositionen%20Framtidens,lokal%2C%20regional%20och%20nationell%20niv%C3%A5.
    - The overall goal is to support people's opportunities to stay out in nature and practice outdoor life where the right of the public basis for outdoor life.

Municipality level

- Program med riktlinjer för utomhuslek (Program and guidelines for outdoor play)
    - https://kommun.falkenberg.se/media/evolution/4ce8393f-fcbc-4585-a2a0-55a2de007de6/592d53ee-0ef6-4787-b85e-4a9786501e2a.pdf
    - This is a municipal document from Falkenberg's municipality

Name and describe fundings bodies that support OP.

None in particular; however, our national funders may fund OP research e.g., if it is linked to living conditions or health implications.

OTHER

In Sweden there is definitively a need for more research in this area and the concept of outdoor play needs to be discussed and emphasized more on the policy level.

Country: Wales

Name of assessor: Marianne Mannello

Date of assessment: 19 November 2024

Data form for outdoor play research, practice, and policy in Europe

This data form on outdoor play (OP) is to be completed in reference to the past 10 years and the country whom the assessor is representing.

OP is defined as a form of play that takes place outdoors [1]. Play is defined as voluntary engagement in an activity that is fun and/or rewarding and usually driven by intrinsic motivation. Please observe that this can refer to both children and adults. This document should not exceed two pages in total.

TERMINOLOGY

The term OP might not be directly or perfectly translated to the language/languages spoken in the country in question. Please name and describe terms that are used in country in question that could be conceptualised as or include outdoor play. Please also comment on how physical activity relates to these terms.

Outdoor play is term that is used widely in the UK. There is sometimes a tendency to equate outdoor play with play in nature and there is also sometimes a focus on early years.<br><br>There has been some work in Wales focused on making better use of community assets for play, for example—developing play streets and use of school grounds for play when the school day ends.<br><br>It is assumed that outdoor play is related to physical activity. The Active Healthy Kids Report Card Wales uses data from surveys with children relating their opportunities to play outdoors.<br><br>Risky play and adventurous play are used interchangeably and are accepted as linked to outdoor play, primarily because concerns about risk are a clear barrier to outdoor play.

RESEARCH

Name active OP researchers and describe their research focus.

**Many researchers in Wales are interested in play. It is rare for a researcher to focus on outdoor play specifically. Instead, researchers tend to focus on play in education, play and humour, play and wellbeing, physical literacy, outdoor learning, play in public spaces/use of space.**<br><br>**National Centre for Population Health & Wellbeing Research—**brings together researchers, data analysts and statisticians to understand and support health and wellbeing. Projects include:

[1] Lee, Eun-Young, Louise de Lannoy, Lucy Li, Maria Isabel Amando de Barros, Peter Bentsen, Mariana Brussoni, Lindsay Crompton, et al. "Play, Learn, and Teach Outdoors—Network (PLaTO-Net): Terminology, Taxonomy, and Ontology." *International Journal of Behavioral Nutrition and Physical Activity* 19, no. 1 (June 15, 2022): 66. https://doi.org/10.1186/s12966-022-01294-0.

- HAPPEN is an established school network that brings together education, health and research to help schools have a better understanding of pupil's physical, psychological, emotional and social health.
- RPlace-a mobile app/web based project that enables children to feedback and make suggestions about places where they live and play.

Making Space for Nature -Professor Merideth Gattis (School of Psychology) and Dr. Matluba Khan (School of Geography and Planning) at Cardiff University collaborate on an academic and enterprise partnership centred around the aim of working with local communities to co-produce plans for making space for nature.

Public Map Platform- a two-year research initiative led by Cambridge University which is piloting a public map to help local authorities and their communities' picture what is happening in a place as a basis for informed decision making and local action on climate change. The Welsh local authority Isle of Anglesey is the pilot area.

Describe scope of scientific publications and reports on OP stemming from country in question (please include references of seminal publications and publications that relate to gender, race, class, or climate).

Not related exclusively to OP, but worth noting:

Dallimore, D. (2023) *What children say about play in Wales.* Cardiff: Play Wales

Mannello, et al (2019) *Opening the school gates: Facilitating after-school play in school grounds* in *Thinking about Pedagogy in Early Education,* Alma Fleet and Michael Reed. eds.*)* London and New York: Routledge.

Play sufficiency research studies: https://play.wales/play-policy-legislation/our-research/

Richards, Amie B.; Mackintosh, Kelly A.; Swindell, Nils; Ward, Malcolm; Marchant, Emily; James, Michaela; et al. (2022). WALES 2021 Active Healthy Kids (AHK) Report Card: The Fourth Pandemic of Childhood Inactivity. Cardiff Metropolitan University. Journal contribution. https://hdl.handle.net/10779/cardiffmet.20227182.v1

Russell, W., Barclay, M. and Tawil, Ben (2024). *Playing and being well A review of recent research into children's play, social policy and practice, with a focus on Wales.* Cardiff: Play Wales

PRACTICE

Name and describe organizations (e.g., Outdoor Play Canada) that support and promote OP.

Most of these organisations advocate for children's play or represent stakeholders within the play sector. Play and playwork provision is delivered at a local level through public play spaces, playschemes, holiday and before/after school clubs, schools.

Play Wales https://play.wales
Play Wales, the national charity for play in Wales advocates for the right and need for all children to play. Our key areas:

- raising awareness
- promoting good practice
- providing advice and guidance across all sectors

Our work includes:

- Policy: Working with others to inform the development of policy and other children's play issues
- Information service: Promoting the value of children's play by providing timely and current information
- Advice and support: Providing specialist knowledge about all issues that affect children's play
- Workforce development: Contributing to the professional development of the playwork and play workforces

The Playwork Education and Training Council for Wales (PETC Wales) is a group that discusses issues of strategic importance to all aspects of playwork education, training and qualifications in Wales. The group also makes recommendations to PETC UK. Play Wales supports the running of PETC Wales.

International Play Association IPA Cymru Wales
IPA Cymru Wales is the Welsh branch the International Play Association, the global child rights advocacy network dedicated to promoting a child's right to play. We recognise the importance of ensuring that children are supported in their fundamental right to play. The work, values and principles of IPA Cymru Wales are underpinned by the United Nations Convention on the Rights of the Child (UNCRC) and in particular Article 31.

Name and describe networks (e.g., PLaTO-Net) that support and promote OP.

Playday https://www.playday.org.uk/
Playday is a campaign that highlights the importance of play in children's lives and focuses on a different issue each year. Playday is coordinated by Play England, Play Scotland, Play Wales, and PlayBoard Northern Ireland.

Playful Childhoods- a Play Wales campaign that aims to help parents and community groups to provide more opportunities for children to play at home and in their neighbourhoods. Playful Childhood aims to support:

- Parents in giving their children opportunities to play.
- Parents so they feel confident about letting their children play outside in the community.
- The development of playful communities for children across Wales.

- A shared understanding of the importance of play for children and teenagers by all adults across Wales.

A central part of the campaign is the website: **https://playfulchildhoods.wales**

Children's Play Policy Forum- **https://childrensplaypolicyforum.wordpress.com**

This UK wide forum works to champion, promote and increase people's understanding of the importance of children's play and quality, inclusive play provision.

UK Play Safety Forum- **https://playsafetyforum.wordpress.com**

This forum promotes a balance between safety, risk and challenge within play and leisure provision. It identifies, develops and provides advice and guidance that:

- determines and promotes a balanced and thoughtful approach to risk, challenge, benefits and safety
- advises on policy and practice relating to risk benefit and safety in the places where children play
- makes advice available to government departments, agencies and regulators.

POLICY

Name and describe national policy documents that support OP.

Legislation

Children and Families (Wales) Measure 2010 - addresses the commitment to tackle child poverty. Importantly, this measure also covers play and participation.

Part of the measure places a legal duty on every local authority in Wales to assess and secure sufficient play opportunities for children in their area, in accordance with the Play Sufficiency Assessment (Wales) Regulations.

The Well-being of Future Generations (Wales) Act 2015 places a requirement on public bodies in Wales to:

- think about the long-term impact of their decisions
- work better with people, communities and each other
- prevent problems
- take a more joined-up approach.

Rights of Children and Young Persons (Wales) Measure 2011 embeds consideration of the UNCRC and the optional protocols into Welsh law. The aim of this was to provide an effective way of ensure that greater emphasis would be placed on helping children in Wales to access the rights set out in the UNCRC, as a step to them all contributing to their wellbeing.

The Well-being of Future Generations (Wales) Act 2015 places a requirement on public bodies in Wales to:

- think about the long-term impact of their decisions
- work better with people, communities and each other
- prevent problems
- take a more joined-up approach.

Welsh Government Ministerial Review of Play- between 2019 and 2022, the Welsh Government undertook an in-depth and collaborative review of its play policy work**.** The review included a theme of spatial justice. In the context of the review, spatial justice is about children's fair and just access to – and participation in – what public space has to offer. The focus here is on general public space and particularly neighbourhoods. Spatial justice is therefore about children's everyday freedoms[i] to move around their neighbourhoods and play or meet up with friends.

Public Health Wales

- *Every Child Wales* lists outdoor play as one of the evidenced 10 steps to a health weight
- Public Health Wales has issued a resource and a template for supplementary planning guidance on *Planning and Enabling Healthy Environments*, aimed at supporting planning authorities in their Local Development Planss. This document explicitly talks about child-friendly neighbourhoods that can support play, in terms of specific play areas, streets that are safe and playable, and safe, active travel routes.

The Welsh Government's Framework on embedding a whole school approach to emotional and mental well-being-includes a section titled 'The importance of play'.

Curriculum for Wales, builds on the approach and principles developed through the Foundation Phase. The new curriculum provides a good opportunity for creativity-led learning. The purpose of every school's curriculum will be to support children of all ages to be:

- "ambitious, capable learners, ready to learn throughout their lives;
- enterprising, creative contributors, ready to play a full part in life and work;
- ethical, informed citizens of Wales and the world;
- healthy, confident individuals, ready to lead fulfilling lives as valued members of society".

To support the workforce to embed play within learning structures particularly as part of the recovery from the pandemic, the Welsh Government has developed national professional development training modules. An online module about play has been developed that explores the relationship between play and learning. This is intended to support teaching staff with embedding play as part of the Curriculum for Wales.

*Enabling Pathways***,** additional guidance for all learners in the period of learning leading to Progression Step 1, stresses that play and playful learning must be considered when schools or settings are designing their new curriculum. The guidance notes that free play is important and should be one of the pillars of delivery within the early years. A curriculum for non-maintained settings that delivers education for three to four year olds has also been developed to support the sector to implement the Curriculum for Wales. Throughout this curriculum, play is recognised as the main vehicle for learning for our youngest children. The guidance notes that the outdoor environment is important in supporting a young child's development.

Name and describe fundings bodies that support OP.

There are no funding bodies in Wales that specifically and uniquely support OP.

The National Lottery Community Fund (UK-wide)

- This fund provides grants for community-led projects that bring people together and improve community cohesion. It operates under different names across the UK: the National Lottery Community Fund England, the National Lottery Community Fund Wales, and the National Lottery Community Fund Northern Ireland.
- The fund supports projects that create or improve spaces for outdoor play, promote children's well-being through outdoor activities, and increase access to nature. Examples include playground renovations, forest school programs, and community gardens.

https://www.tnlcommunityfund.org.uk/

The People's Postcode Lottery (UK-wide)

- The People's Postcode Lottery distributes funds raised by lottery players to a variety of charities and community groups across the UK, including those focused on environmental sustainability, community cohesion, and youth development.
- Funds projects that enhance children's experiences in the outdoors, support outdoor education, and develop community-based outdoor play initiatives. This can include creating natural play spaces or supporting outdoor learning programs.

https://www.postcodelottery.co.uk/

Children in Need (UK-wide)

- A charity that provides grants to projects helping children and young people facing disadvantage.
- Funds projects that use outdoor play and activities to promote physical and mental well-being. This can include adventure play, outdoor learning, and therapeutic play for vulnerable children.

https://www.bbcchildreninneed.co.uk/

OTHER

[i] This is a term used by Tim Gill (2021) cited above. It refers to children's ability to both move freely around their neighbourhoods and also to what neighbourhood public space offers/affords for playing.
